# Exploring the Mechanism of Action of Berberine on Arrhythmia After Myocardial Infarction: A Network Pharmacology, Molecular Docking, and Cellular Experimental Study

**DOI:** 10.1155/cdr/5632985

**Published:** 2025-06-02

**Authors:** Haitian-Li, Zhijie-Zhang, Yang-Yang, Xiaohan-Yu, Jinmao-Li, Hongsheng-Cui, Ping-Li, Buxing-Chen

**Affiliations:** ^1^Department of Cardiovascular, Beijing University of Chinese Medicine, Beijing, China; ^2^Department of Cardiovascular, Beijing University of Chinese Medicine, Third Affiliated Hospital, Beijing, China; ^3^Department of Cardiovascular, The Second Hospital of Shandong University, Jinan, Shandong, China; ^4^Department of Cardiovascular, Tsinghua University Hospital of Integrated Traditional Chinese and Western Medicine, Beijing, China

## Abstract

**Background:** Arrhythmia after myocardial infarction, a common disease, has a high incidence and lethality in clinical practice, which seriously affects patients' quality of life and survival time. Based on our previous study and available evidence, berberine plays a role in the treatment and prevention of arrhythmia after myocardial infarction. Thus, in order to clarify the specific mechanism and provide new clinical treatments, we conducted this study.

**Method:** Firstly, we used bioinformatics analysis and system pharmacology to analyze the physicochemical properties and biological activities of berberine in the Molinspiration server. Secondly, we explored the potential molecular mechanism of arrhythmia after myocardial infarction treated with berberine by using network pharmacology technology: (1) obtaining common genes among berberine, myocardial infarction, and arrhythmia through TCMSP, TTD databases, and so forth; (2) constructing protein–protein interaction by using STRING database; (3) using g:Profiler database to conduct GO enrichment analysis of hub genes and pathways; and (4) performing molecular docking and visualization by using AutoDock and Pymol software. Finally, we applied Western blotting analysis and real-time quantitative polymerase chain reaction to validate the expression of relevant proteins in the TGF-*β*1-induced cell models.

**Results:** The results of bioinformatics analysis and system pharmacology of berberine indicated that it had wonderful bioavailability and high biological activities. The results of network pharmacology showed that (1) 70 genes related to berberine against arrhythmia after myocardial infarction were obtained, (2) 31 hub genes were obtained by constructing PPI network, and (3) GO enrichment analysis showed that hub genes were associated with mechanisms such as stimulus and cell death. The analysis of KEGG pathways, Wiki pathways, and Reactome pathways showed that the HIF-1 signaling pathway and interleukin-4 and interleukin-13 signaling pathways were the most likely to exert therapeutic effects. (4) The results of molecular docking indicated that berberine most likely exerted therapeutic effects through acting on NGF. Western blotting analysis and real-time quantitative polymerase chain reaction techniques showed that berberine could reduce the expression of NGF and *α*-SMA in TGF-*β*1-induced cell models, which confirmed the accuracy of the above findings.

**Conclusion:** Berberine can reduce NGF secretion not only by inhibiting the conversion of cardiac fibroblasts to myofibroblasts but also by acting directly on myofibroblasts. Thus, the sympathetic nerve remodeling was inhibited, which can reduce the occurrence of arrhythmia after myocardial infarction. Considering its wonderful bioavailability and high biological activities, we believe that berberine can be a novel potential therapeutic agent with potential for the treatment of arrhythmia after myocardial infarction.

**Summary**

• Our previous cellular experiments have shown that berberine (BBR) can inhibit the postinfarction inflammatory response and transforming growth factor-*β*1 (TGF-*β*1) expression [1].
• When our preliminary study is combined with the results of network pharmacology, bioinformatics analysis, molecular docking, and cellular experiments, we find that BBR can reduce the conversion of cardiac fibroblasts (CFS) to myofibroblasts and the secretion of NGF either by inhibiting the postinfarction inflammatory response and TGF-*β*1 expression or directly inhibiting NGF secretion by myofibroblasts. Through the abovementioned pathways, it reduces sympathetic remodeling after infarction and inhibits the occurrence of arrhythmias.
• BBR has good physicochemical properties and biological activities and can be better developed and applied in clinical practice for the treatment of arrhythmia after myocardial infarction.

## 1. Introduction

Acute myocardial infarction (AMI) is a type of acute coronary syndrome (ACS), which is traditionally classified into ST-segment elevation and non-ST-segment elevation, and its diagnosis is mainly based on clinical symptoms, electrocardiogram, and laboratory tests [2]. With the improvement of emergency thrombolysis, percutaneous coronary intervention (PCI), and antithrombotic therapy, the mortality rate of AMI has decreased [3–6]. However, the malignant arrhythmias caused by the infarction also seriously affect the quality of life of human beings, and even the occurrence of sudden cardiac death seriously endangers life. A study on the characteristics of patients with myocardial infarction showed that the incidence of atrial fibrillation in patients with myocardial infarction increased from 4.1%~6.1% to 9.4%~11.7% in the past 10 years [7]. Another retrospective analysis of more than 19,000 patients with ST-segment elevation myocardial infarction reported that early application of antiarrhythmic drugs reduced mortality. On the other hand, patients with ventricular tachycardia (VT) and ventricular fibrillation (VF) caused by ischemia had higher long-term mortality than patients with myocardial infarction [8, 9]. Therefore, prevention and control of arrhythmias is an indispensable step in the treatment of myocardial infarction.

Accumulating evidence has firmly established a profound relationship between TGF-*β*1 and arrhythmia after myocardial infarction. The expression of TGF-*β*1 increases significantly after myocardial infarction. It can promote the proliferation and differentiation of CFS, enabling them to synthesize and secrete a large amount of extracellular matrix proteins, such as collagen, resulting in myocardial fibrosis. Myocardial fibrosis disrupts the normal structure and function of the myocardial tissue, alters the electrophysiological properties of cardiomyocytes, and thus increases the risk of arrhythmia [10, 11].

Moreover, TGF-*β*1 can regulate the expression and function of ion channels in cardiomyocytes through multiple signaling pathways. For example, it can affect L-type calcium channels and other ion channels, thereby triggering arrhythmia [12]. Gap junction proteins between cardiomyocytes are crucial for maintaining the electrical coupling and normal electrophysiological activities of cardiomyocytes. TGF-*β*1 can downregulate the expression and distribution of connexin 43, leading to abnormal electrical signal conduction between cardiomyocytes, an increase in the heterogeneity of myocardial electrical activity, and the easy formation of reentrant excitation, which in turn induces arrhythmia [13, 14].

**Figure 1:**
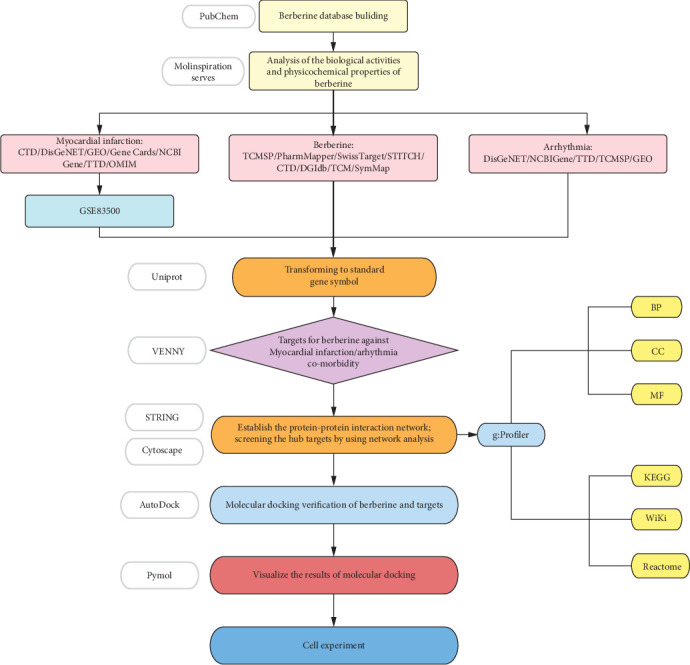
The flow diagram of this research showing a pragmatic strategy for identifying the pharmacological mechanisms of berberine against arrhythmia after myocardial infarction based on system pharmacology and bioinformatics analysis.

BBR is an extract of the traditional Chinese medicine Huanglian, which is widely used in the treatment of heart disease, especially in the treatment of arrhythmias. It can be used to treat different types of arrhythmias through multiple channels and multiple targets [15, 16]. It has been shown that BBR can prevent the occurrence of atrial fibrillation by prolonging the action potential and effective refractory period of atrial myocytes. Apart from this, it can also target different types of K+ channels to achieve Class III antiarrhythmic effects [17–20]. In addition, some studies have shown that BBR can prevent arrhythmias after myocardial infarction in rats, but its mechanism is not fully studied [21]. Therefore, we used the Molinspiration server to simulate and analyze the molecular properties and bioactivity scores of BBR. Then, we used network pharmacology, molecular docking, and cellular experiments to investigate the potential mechanisms of BBR against arrhythmias after myocardial infarction. The specific process is shown in Figure 1.

## 2. Materials and Methods

### 2.1. Data Source Statement

All data in the text are taken from open databases and no interests are involved in this study.

### 2.2. Establishment of the BBR Database

PubChem (https://pubchem.ncbi.nlm.nih.gov/), which involves 750 data sources of chemical information, was used to obtain BBR's 2D structure, 3D structure, and Canonical SMILES expressions [22], as seen in Table 1.

### 2.3. Analysis of the Biological Activities and Physicochemical Properties of BBR

We obtained BBR-related information from the Molinspiration server (https://www.molinspiration.com/). Molecular properties and bioactivity scores were calculated by the Molinspiration server. Based on Lipinski's rule of five [23], which includes *n*-violations, miLogP, MW, *n*-OHNH, and *n*-rotb, we evaluated the solubility and absorption of BBR. Subsequently, to obtain the biological activities of BBR, we calculated the following items: GPCR ligand, ion channel modulator, kinase inhibitor, nuclear receptor ligand, protease inhibitor, and enzyme inhibitor [24].

### 2.4. Acquisition of BBR-Related Target Genes

Firstly, BBR-related target genes were gathered from the following different databases: (1) Traditional Chinese Medicine Systems Pharmacology Database and Analysis Platform (TCMSP) (https://old.tcmsp-e.com/tcmsp.php); (2) PharmMapper (http://lilab-ecust.cn/pharmmapper/index.html) [25–27]; (3) Swiss Target Prediction (http://swisstargetprediction.ch/) [28]; (4) Chemical Association Networks (STITCH, http://stitch.embl.de/) [29]; (5) Comparative Toxicogenomics Database (CTD) (http://ctdbase.org/) [30]; (6) Drug–Gene Interaction Database (DGIdb) (https://dgidb.genome.wustl.edu/downloads) [31]; (7) Encyclopedia of Traditional Chinese Medicine (ETCM) (http://www.tcmip.cn/ETCM/index.php/Home/Index/All) [32]; and (8) Symptom Mapping (http://www.symmap.org/). Then, by leveraging the UniProt database (https://www.uniprot.org/uploadlists/), BBR-related target genes taken from this series of origins were converted into standard gene symbols.

### 2.5. Acquisition of Target Genes Related to Arrhythmia and Myocardial Infarction

To acquire the target genes of myocardial infarction, we reviewed the following different databases: (1) CTD (http://ctdbase.org/) [30]; (2) DisGeNET (https://www.disgenet.org/) [33, 34]; (3) Gene Expression Omnibus (GEO) database (https://www.ncbi.nlm.nih.gov/geo/) [35]; (4) Gene Cards (https://www.genecards.org/); (5) NCBI Gene (https://www.ncbi.nlm.nih.gov/); (6) Therapeutic Target Database (TTD) (http://db.idrblab.net/ttd/) [36]; and (7) Online Mendelian Inheritance in Man (OMIM) (https://www.omim.org/) [37]. Besides, we used the analyze with GEO2R function carried in the GEO database, setting *p* value < 0.05, |log2FC| > 1, to obtain the additional target genes of myocardial infarction from a GEO dataset GSE83500. We selected the GEO2R analysis function in the GEO database and adopted the Benjamini and Hochberg (false discovery rate) option to control the false positive rate, ensuring that the genes obtained through the screening are statistically significant.

Target genes related to arrhythmia were acquired from the following databases: (1) DisGeNET (https://www.disgenet.org/); (2) NCBIGene (https://www.ncbi.nlm.nih.gov/); (3) TTD (http://db.idrblab.net/ttd/) [36]; (4) GEO database (https://www.ncbi.nlm.nih.gov/geo/); and (5) TCMSP.

### 2.6. Obtaining Target Genes of BBR for Arrhythmia After Myocardial Infarction

The approach to obtain the target genes for BBR, myocardial infarction, and arrhythmia was carried out through VENNY 2.1 (https://bioinfogp.cnb.csic.es/tools/venny/), and the crossover part obtained above was regarded as target genes of BBR for the treatment of arrhythmia after myocardial infarction.

### 2.7. Building PPI (Protein–Protein Interaction) Network to Identify the Hub Targets

We used STRING 11.5 (https://cn.string-db.org/cgi/) to create a PPI network. The option of “meanings of network edges” was set to evidence, and the option of “minimum required interaction score” was set to medium confidence (0.400). The options of network display was set as follows: disable structure previews inside network bubbles and hide disconnected nodes in the network and then exported the TSV format file [38]. To visualize the information in the TSV file, we used Cytoscape V3.9.1 to calculate nodes' scores. Eventually, according to the degree values obtained from the calculation and ranked from highest to lowest, target genes with values higher than the average were selected as hub genes for further analysis [39].

### 2.8. GO Enrichment Analysis and the Search of Biological Pathways for Hub Genes

In order to obtain the comorbidity of BBR, arrhythmia, and myocardial infarction, hub targets were transferred into g:Profiler (https://biit.cs.ut.ee/gprofiler/gost) for GO enrichment analysis [40], which contained three parts: (1) molecular function (MF); (2) cellular component (CC); and (3) biological process (BP). Then, the next step was to acquire the biological pathways from (1) KEGG pathways, (2) Reactome pathways, and (3) Wiki Pathways, with the organism setting to *Homo sapiens* (human). Determined results were ranked according to the adjusted *p* value from smallest to largest.

### 2.9. Performing Molecular Docking of BBR and Target Proteins

We obtained BBR 3D structure from PubChem [22], and we obtained the protein structures expressed by hub genes from PDB database (https://www.rcsb.org/) [41]. In AutoDock4 software (https://autodock.scripps.edu/), the acquired BBR 3D structure and the protein structures were processed by removing water molecules and adding hydrogen atoms. Normally, the drug molecular structure of BBR and the treated protein structure expressed by hub genes were called the ligand molecule and the receptor protein, respectively. Normally, the binding energy value ≤ −5.0 kcal/mol was considered a more stable connection. AutoDock4 is a wonderful software to calculate the energy of binding energy between the ligand molecule and the receptor protein. In this software, we set the Grid box parameters and calculated and analyzed the binding energy between the ligand molecule and the receptor protein. Finally, we visualized the binding site of the ligand molecule with the highest binding energy value to the receptor protein by using Pymol 2.5 software (https://pymol.org/2/).

### 2.10. Construction of Cellular Models

Clean-grade Sprague–Dawley (SD) rats of 1~3 days old were purchased from Hua-Fukang (Beijing, China). Then, each of the rats' hearts was fully exposed. Holding the lower edge of the sternum with tissue forceps and cut along the lower edge of the sternum with tissue scissors. Then, separate the rat's ventricles with ophthalmic scissors and cut away the excess blood vessels and tissues. Separated ventricles were washed twice with PBS and then transferred to serum vials, and the rat heart tissues were quickly cut with small straight scissors, and 5 mL of 400 KU/L I collagenase (Sigma-Aldrich, St. Louis, MO, United States) digestion solution was added and shaken at 37°C in a water bath at the speed of 150 r/min. The digestion was performed 5–6 times consecutively for 4 min each time and then collected and immediately mixed with 5-mL Dulbecco's modified Eagle's medium (DMEM). All the collected cells were filtered through a sieve and centrifuged at 1200 r/min for 5 min; next, they were placed in a 10-cm flat dish with 5% CO_2_ and left for 2 h at 37°C. The isolation of rat CFS was performed by the differential apposition method. After being cultured, the third-generation CFS were used in this experiment. The approval of this investigation had been granted by the Ethics Committee of Beijing University of Chinese Medicine Third Affiliated Hospital.

### 2.11. Grouping of Cell Models

**Figure 2:**
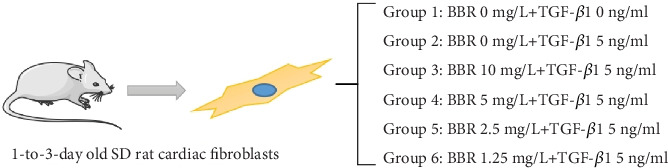
The specific grouping of cell models.

The obtained cells were divided into the blank group, control group, and BBR high, medium, low, and micro groups and then cultured for 72 h. The selected drugs TGF-*β*1 (Sigma-Aldrich, St. Louis, MO, United States) and BBR (Sigma-Aldrich, St. Louis, MO, United States) were grouped and treated as follows: the blank group: CFS + serum-free culture medium; control group: CFS + serum-free culture medium + TGF-*β*1 5 ng/mL; BBR high-dose group: CFS + serum-free culture medium + BBR 10 mg/L + TGF-*β*1 5 ng/mL; BBR medium-dose group: CFS + serum-free culture medium + BBR 5 mg/L + TGF-*β*1 5 ng/mL; BBR low-dose group: CFS + serum-free culture medium + BBR 2.5 mg/L + TGF-*β*1 5 ng/mL; and BBR micro-dose group: CFS + serum-free culture medium + BBR 1.25 mg/L + TGF-*β*1 5 ng/mL. See Figure 2 for the specific grouping, and the number of replicates for each group was 3.

### 2.12. Western Blotting Analysis and Real-Time Quantitative Polymerase Chain Reaction (PCR)

A bicinchoninic acid (BCA) assay kit (Thermo Fisher Scientific, Waltham, MA, United States) was used to measure the protein concentrations. To identify the corresponding *α*-SMA and NGF proteins, we utilized antibodies against *α*-SMA and NGF (Cell Signaling Technology, Danvers, MA, United States). We labeled them by enhanced chemiluminescence (ECL) and then analyzed them using ImageJ (https://imagej.nih.gov/ij/).

Add 200 ng of the extracted and purified RNA, respectively, and then reversely transcribe the extracted RNA into cDNA using Invitrogen SuperScript III. PCR amplification was performed using the ABI 7900HT Real-Time PCR System, with 2 *μ*L cDNA, 10 *μ*L qPCR mix, 1 *μ*L primer F, 1 *μ*L primer R, and 6 *μ*L ddH_2_O of mixed substrate added. The next step was to amplify them according to the sequence of 95°C for 2 min, 94°C for 20 s, 60°C for 20 s, and 72°C for 30 s for 40 cycles. The specific primers were as follows: AGCGTAATGTCCATGTTGTTC (NGF primer F); AGTCCAGTGGGCTTCAGG(NGF primer R); GTTACCAGGGCTGCCTTCTC(GAPDH primer F); and GGGTTTCCCGTTGATGACC(GAPDH primer R). The relative expressions of the genes were calculated using the 2^-*ΔΔ*Ct^ method.

### 2.13. Further Processing of the Obtained Data

Cell experiment data analysis was performed using IBM SPSS Statistics 25.0. One-way ANOVA was used for data that conformed to normal distribution and chi-square, and nonparametric analysis was used for data that did not conform to normal distribution. It would be considered that there was a statistically significant difference between the two groups of data when *p* < 0.05.

## 3. Analysis of Results

### 3.1. Analysis of Physicochemical Properties of BBR

According to Lipinski's rule of five, the comparative results were shown as follows: miLogP = 0.20 < 4.15, MW = 336.37 < 500, *n* − OHNH = 0 < 5, *n* − violations = 0 < 1, and *n* − rotb = 2 < 10, indicating that BBR has wonderful solubility and permeability, as shown in Table 2.

### 3.2. Analysis of Biological Activities of BBR

BBR bioactivity scores are shown in Table 3. As shown, BBR may act through multiple pathways, including interactions with GPCR ligands, ion channel modulators, kinase inhibitors, nuclear receptor ligands, protease inhibitors, and enzyme inhibitors. It is generally considered that a substance has considerable activity when its biological activity score is > 0, moderate activity when the score is between −0.5 and 0, and no activity when the score is < −0.5 [19]. The biological activity scores of BBR are ranked in the following order: enzyme inhibitor > ion channel modulator > 0 > GPCR ligand> kinase inhibitor>protease inhibitor>−0.5. From this, it can be seen that BBR exerts its physiological effects through various pathways, and among them, enzyme inhibitors and ion channel modulators are the most likely to interact.

### 3.3. BBR, Myocardial Infarction, and Arrhythmia Target Genes and Common Genes

**Figure 3:**
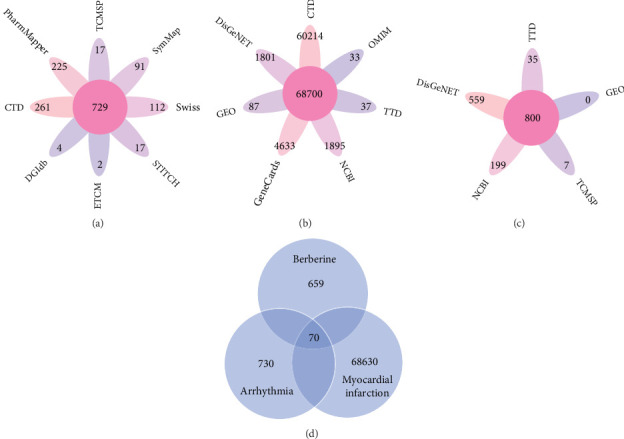
(a) The target genes related to berberine. (b) The target genes related to myocardial infarction. (c) The target genes of arrhythmia. (d) Venn diagram depicting common target genes between berberine, myocardial infarction, and arrhythmia.

Finally, a total of 729 BBR-related target genes were obtained from eight databases, as shown in Figure 3a.

Then, 68,700 myocardial infarction target genes were acquired as shown in Figure 3b. GSE83500, which was from the GEO database, contained RNA data of ascending aortic wall puncture biopsy tissues from 17 myocardial infarction patients and 20 nonmyocardial infarction patients.

A total of 800 arrhythmia target genes were obtained as shown in Figure 3c. By taking intersections, we finally obtained 70 common genes between BBR, myocardial infarction, and arrhythmia, which were displayed by VENNY 2.1, as shown in Figure 3d.

### 3.4. PPI Network Analysis

**Figure 4:**
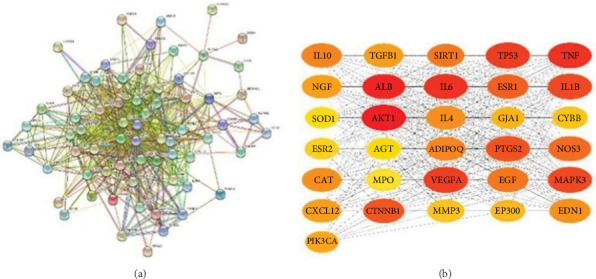
(a) PPl network for hub targets of berberine against arrhythmia/myocardial infarction comorbidity. (b) 31 hub genes network performed by Cytoscape.

To establish the PPI network and obtain the hub genes, STRING 11.5 (https://cn.string-db.org/) was used. In the PPI network, the nodes represented different proteins and the links represented protein–protein associations. Overall, 68 nodes and 699 links were included in the PPI network, as shown in Figure 4a. Then, we exported the TSV format file, visualized the protein interaction information in the TSV file using Cytoscape V3.9.1 software, and calculated nodes' scores using cytoHubb. According to the degree values ranked from highest to lowest, 31 hub genes with values greater than the mean value (20) were selected for further analysis, as shown in Figure 4b.

### 3.5. GO Enrichment Analysis of the Obtained Hub Genes

**Figure 5:**
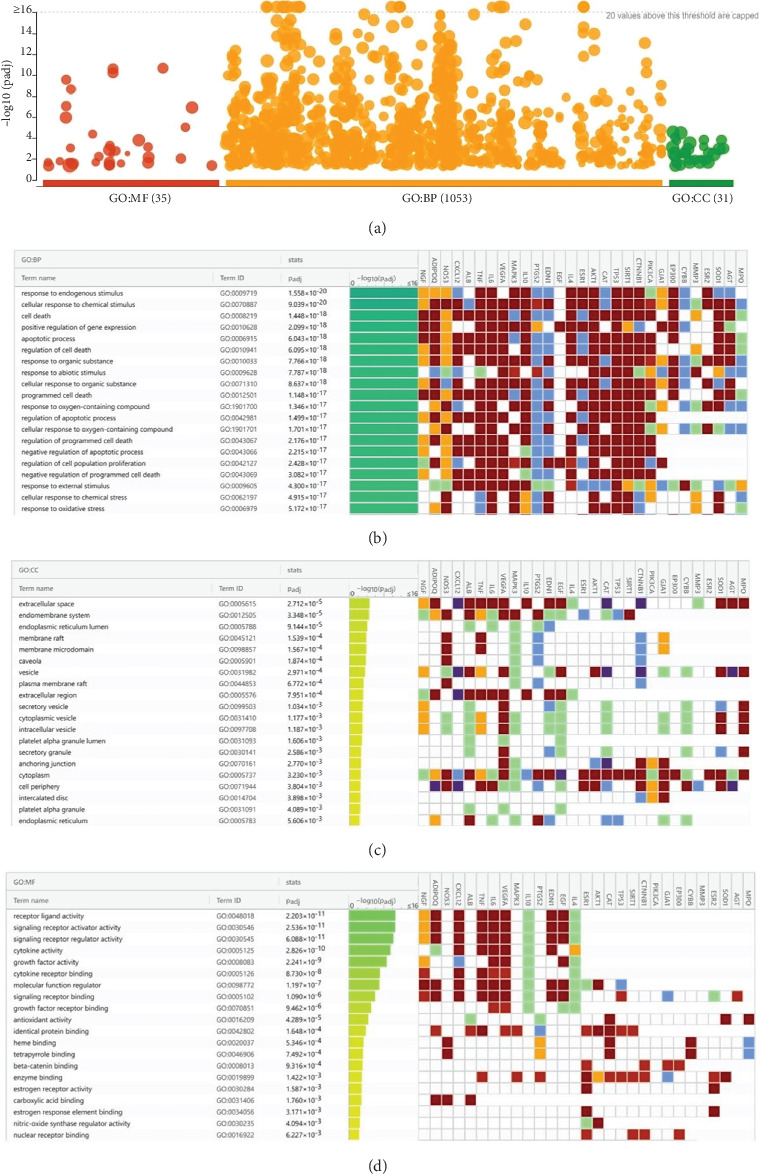
(a) The results of biological process (BP), cellular component (CC), and molecular function (MF) enrichment analysis. (b) ldentification result of BP terms. (c) ldentification result of CC terms. (d) Identification result of MF terms.

A total of 35 MF terms, 1053 BP terms, and 31 CC terms were obtained as shown in Figure 5a. Complete lists are provided in Table S1. The Top 20 MF terms, BP terms, and CC terms were sorted by the adjusted *p* value in ascending order and displayed in Figures 5b, 5c, and 5d. MF terms mainly included receptor ligand activity, signaling receptor activator activity, and so forth. CC terms mainly included extracellular space, endomembrane system, and so forth. BP terms mainly included response to endogenous stimulus, cellular response to chemical stimulus, and so forth. The results of GO enrichment analysis suggested that BBR may act on arrhythmia after myocardial infarction by binding to receptor ligands on the surface of target cells in response to stimuli that may lead to apoptosis, endogenous or exogenous stimuli, and so forth.

### 3.6. Pathway Enrichment Analysis

**Figure 6:**
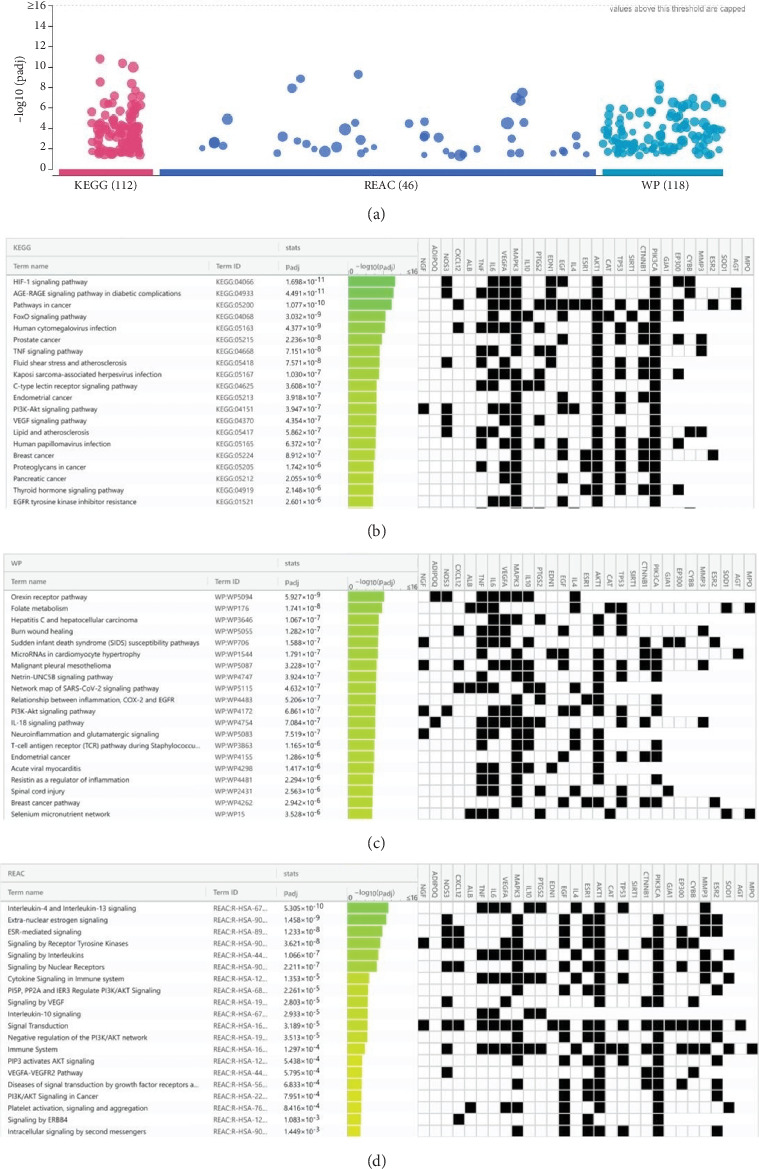
Biological pathway enrichment analysis results. (a) The results of KEGG, Wiki, and Reactome pathway enrichment analyses. (b) KEGG pathway enrichment analysis identification result. (c) Wiki pathway enrichment analysis identification result. (d) Reactome pathway enrichment analysis identification result.

Using g:Profiler to analyze the selected hub genes' pathways, we obtained 112 KEGG pathways, 46 REAC pathways, and 118 Wiki pathways, as shown in Figure 6a. Complete lists are provided in Table S2. The Top 20 KEGG pathways, REAC pathways, and Wiki pathways were ranked in descending order of adjusted *p* value, as shown separately in Figures 6b, 6c, and 6d. The KEGG pathways included the HIF-1 signaling pathway, AGE-RAGE signaling pathway in diabetic complications, and so forth. REAC pathways included interleukin-4 and interleukin-13 signaling, extranuclear estrogen signaling, and so forth. The Wiki pathways included orexin receptor pathway, folate metabolism, and so forth. The results of the pathway analysis indicated that BBR may play a role in arrhythmia after myocardial infarction through leukocyte-mediated signaling pathways and so forth.

### 3.7. Molecular Docking Analysis

**Figure 7:**
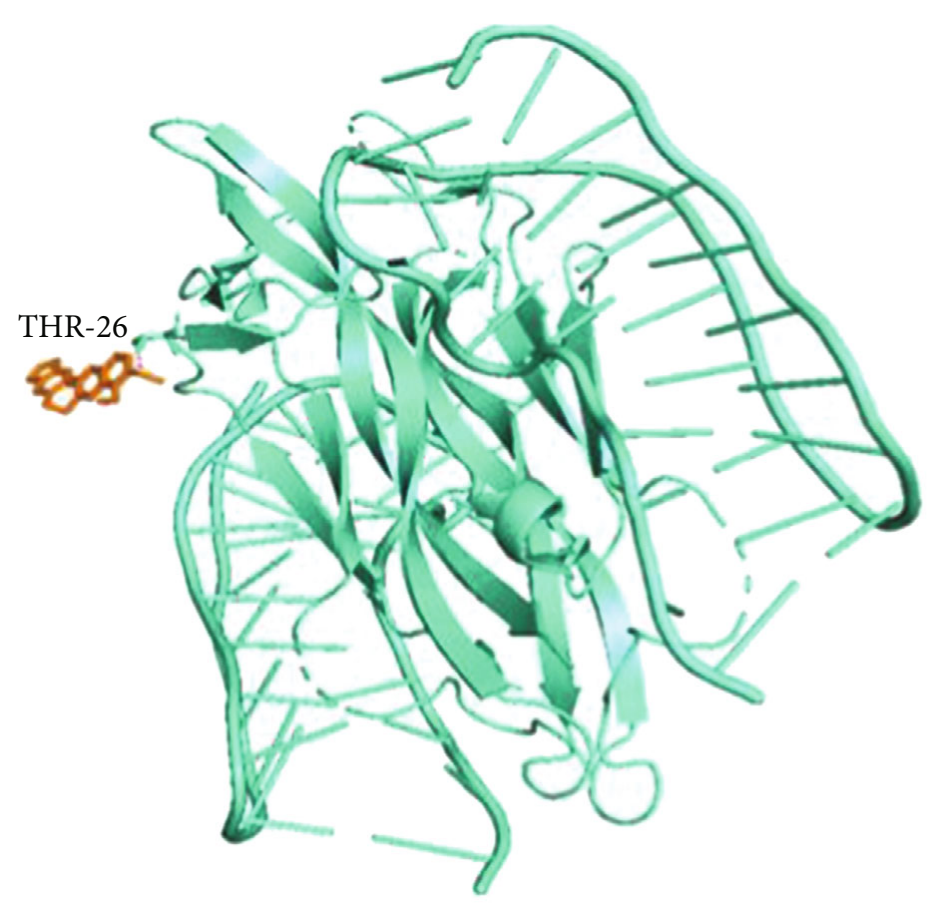
The docking model of berberine with 4ZBN.

According to hub genes' degree values of hub genes ranked from highest to lowest, the Top 20 receptor proteins (containing 22 receptor proteins in total) with the highest degree value were selected for molecular docking with BBR ligand molecules. The results showed that BBR had good binding activities with the 22 receptor proteins (all binding energies were ≤ −5 kcal/mol), among which BBR ligand molecules required the highest binding energy with NGF receptor proteins, as shown in Table 4. To make the results clearer, the binding of BBR ligand molecules to NGF receptor proteins was visualized by Pymol software, and it was found that BBR had higher stability by interacting with THR-26 at the end of amino acid residues of NGF receptor proteins through hydrogen bonds as shown in Figure 7. The above results suggested that BBR may play a role in arrhythmia after myocardial infarction by acting on NGF proteins.

### 3.8. Analysis of Cellular Experiment Results

**Figure 8:**
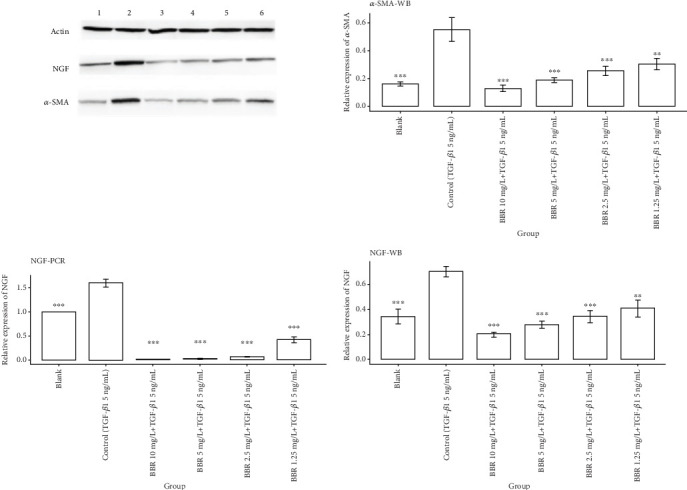
Cellular experiment results. Note: ⁣^∗^ represents *p* < 0.05, ⁣^∗∗^ represents *p* < 0.01, and ⁣^∗∗∗^represents *p* < 0.001. 1: blank group; 2: control group; 3: berberine 10 mg/L + TGF-*β*1 5 ng/mL; 4: berberine 5 mg/L + TGF-*β*1 5 ng/mL; 5: berberine 2.5 mg/L + TGF-*β*1 5 ng/mL; and 6: berberine 1.25 mg/L + TGF-*β*1 5 ng/mL.

We first observed *α*-SMA by Western blotting staining, and the staining results showed that *α*-SMA expression was significantly increased in the control group compared with the blank group, and there was a statistical difference between the two groups (*p* < 0.001). Compared with the control group, *α*-SMA protein was significantly decreased in the BBR high-dose group, BBR medium-dose group, and BBR low-dose group, and statistical differences also existed between each group (*p* < 0.001). *α*-SMA expression in the BBR micro dose group was lower than that in the control group, and there was a statistical difference between the two groups (*p* < 0.01). The above results indicated that the model of the TGF-*β*1-induced transformation of CFS into myofibroblasts was successfully constructed [42]. The results of molecular docking showed that the binding energy between BBR and NGF protein was the highest among the tested proteins, so we further observed the NGF expressed in myofibroblasts by Western blotting staining as well as real-time PCR. The Western blotting staining results showed that the NGF expression was significantly lower in all BBR dose groups compared with the control group, and there was a statistical difference between all the dose groups of BBR and the control group (*p* < 0.01). The results of real-time PCR showed that the NGF expression was lower in all the BBR dose groups than in the control group, and there was a statistical difference between the different BBR dose groups and the control group (*p* < 0.001), as shown in Figure 8.

## 4. Discussion

Among the survivors of AMI, approximately 10% experience a decline in left ventricular function and a reduction in ejection fraction. Among these patients, 40% die from arrhythmias [42–44]. Currently, commonly used treatments such as aldosterone receptor blockers or implantable defibrillators have certain effects, but the morbidity and mortality rates remain high. Therefore, we introduced the LianXia Ningxin Formula Granule, a traditional Chinese medicine, for therapeutic intervention [45, 46]. Previous animal experiments have demonstrated that it can inhibit arrhythmias in rats through the NGF/TrKA/PI3K/AKT signaling pathway [47]. Since LianXia Ningxin Formula Granule has more drug components, we analyzed the main effective components of LianXia Ningxin Formula by establishing high-performance liquid chromatography–electrospray ionization–triple quadrupole mass spectrometry (HPLC-ESI-MS/MS). The results indicated that BBR exerted the most extensive biological effects. Consequently, we conducted further investigations on BBR to elucidate its role in patients with arrhythmias following myocardial infarction.

The analysis of the physicochemical properties and biological activities of BBR showed that it complies with Lipinski's rule of five. Its TPSA value is 40.82 Å, which is less than 140 Å, indicating good absorption, distribution, and metabolism in vivo [23, 48]. The biological activity analysis indicated that BBR has high activities in aspects such as enzyme inhibition and ion channel modulation, showing potential for development as a new drug for treating arrhythmias after myocardial infarction.

Through the PPI network analysis, 31 key genes were obtained, including AKT1, PTGS2, TGF-*β*, EGF, NGF, and interleukin-related factors. These genes are involved in multiple cellular processes and signaling pathways and are closely associated with the pathological processes of myocardial infarction and arrhythmias [49–67]. GO enrichment analysis and signaling pathway enrichment analysis suggested that BBR may exert its effects by interfering with processes such as ischemia, hypoxia, and inflammation [68–78].

To further explore the role of BBR in arrhythmias after myocardial infarction, we used the molecular docking technique and found that BBR has the highest binding energy with NGF, resulting in a more stable binding [79]. NGF plays a crucial role in sympathetic nerve remodeling [80]. The sympathetic neurons in the heart innervate the myocardium in a dense and nonrandom topological structure, establish structured connections with cardiomyocytes, and regulate intersynaptic cell communication through neural factors released by cardiomyocytes [1, 81]. Multiple studies have shown that NGF is a key cytokine involved in the sympathetic activity of arrhythmias after myocardial infarction [82–84]. It is closely related to the degree of postinfarction arrhythmias and has received increasing attention as a biomarker for arrhythmias after myocardial infarction.

Further research using TGF-*β*1-induced cell experiments revealed that the expression of NGF in all BBR-treated groups was significantly lower than that in the control group, and this effect was dose-dependent. That is, the higher the dose of BBR, the more obvious the inhibitory effect on NGF expression. This indicates that BBR may reduce sympathetic nerve remodeling by inhibiting the secretion of NGF from myofibroblasts, thereby further reducing the incidence of arrhythmias after myocardial infarction. This result is consistent with existing studies. Meanwhile, the expression of *α*-SMA (a marker of myofibroblasts) in all BBR-treated groups was significantly lower than that in the control group, suggesting that BBR may inhibit the transformation of CFS into myofibroblasts induced by TGF-*β*1. This is the first study to confirm that BBR can reduce the risk of arrhythmia attacks after myocardial infarction by inhibiting the TGF-*β*1-induced transformation of CFS into myofibroblasts and directly inhibiting the secretion of NGF by myofibroblast cells. In the follow-up, we will further explore the specific signaling pathways through which BBR exerts this effect, aiming to provide more evidence for the treatment of arrhythmias after myocardial infarction with BBR.

## Figures and Tables

**Table 1:** Basic information of berberine.

**Name**	**Canonical SMILES**	**2D structure**	**3D structure**
Berberine	(COC1=C(C2=C[N+]3=C(C=C2C=C1)C4=CC5=C(C=C4CC3)OCO5)OC)	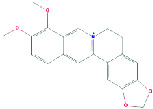	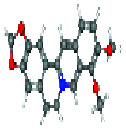

**Table 2:** Physicochemical properties of berberine.

**Name**	**miLogP**	**TPSA (Å)**	**n** **-atoms**	**MW**	**n** **-ON**	**n** **-OHNH**	**n** **-violations**	**n** **-rotb**	**MV**
Lipinski's rule of five	≤ 4.15			≤ 500		≤ 5	≤ 1	≤ 10	
Berberine	0.20	40.82	25	336.37	5	0	0	2	296.30

**Table 3:** Analysis of biological activities of berberine.

**Name**	**GPCR ligand**	**Io channel modulator**	**Kinase inhibitor**	**Nuclear receptor ligand**	**Protease inhibitor**	**Enzyme inhibitor**
Berberine	−0.11	0.71	−0.27	−0.78	−0.35	0.82

**Table 4:** Molecular docking results.

**Rank**	**Ligand**	**Receptor proteins**	**PDB entry**	**Binding energy (kcal/mol)**
1	Berberine	NGF	4ZBN	−8.9
2		ADIPOQ	5LWY	−8.8
3		NOS3	3EAH	−8.7
4		CXCL12	3OE8	−8.5
5		ALB	2BX8	−8.4
6		TGF-*β*	6MC1	−8.3
7		TNF	7KP8	−8.3
8		IL6	7P11	−8.2
9		VEGFA	6BFT	−8.1
10		MAPK3	2ZOQ	−8
11		IL10	3OOJ	−7.9
12		PTGS2	6F2U	−7.8
13		EDN1	5GlH	−7.7
14		EGF	1DQB	−7.6
15		IL4	3BPO	−7.6
16		ESR1	4XI3	−7.4
17		AKT1	3O96	−7.3
18		CAT	5 V84	−7.2
19		TP53	5YJ8	−6.9
20		SIRT1	2ZFU	−6.8
21		CTNNB1	7AFW	−6.8
22		IL1	5TOZ	−6.6

## Data Availability

The data that support the findings of this study are available from the corresponding author upon reasonable request.

## Supporting Information

Supporting Information 1 Table S1: The results of GO enrichment analysis including BP, CC, and MF.

Supporting Information 2 Table S2: The results of pathway enrichment analysis including KEGG, REAC, and Wiki.
