# Supplementary material for: Exploring the Mechanism of Action of Berberine on Arrhythmia After Myocardial Infarction: A Network Pharmacology, Molecular Docking, and Cellular Experimental Study
Source: Cardiovasc Ther. 2025 Jun 2;2025:5632985. doi: 10.1155/cdr/5632985 (PMC12149515; doi:10.1155/cdr/5632985)
Supplement: Supporting Information 1 — Table S1: The results of GO enrichment analysis including BP, CC, and MF. [file 5632985.f1.docx]

| source | term_name | term_id | adjusted_p_value | negative_log10_of_adjusted_p_value | intersections |
| --- | --- | --- | --- | --- | --- |
| GO:MF | receptor ligand activity | GO:0048018 | 2.20E-11 | 10.65689959 | NGF,ADIPOQ,CXCL12,TNF,IL6,VEGFA,IL10,EDN1,EGF,IL4 |
| GO:MF | signaling receptor activator activity | GO:0030546 | 2.54E-11 | 10.59591731 | NGF,ADIPOQ,CXCL12,TNF,IL6,VEGFA,IL10,EDN1,EGF,IL4 |
| GO:MF | signaling receptor regulator activity | GO:0030545 | 6.09E-11 | 10.21552882 | NGF,ADIPOQ,CXCL12,TNF,IL6,VEGFA,IL10,EDN1,EGF,IL4 |
| GO:MF | cytokine activity | GO:0005125 | 2.83E-10 | 9.54877811 | ADIPOQ,CXCL12,TNF,IL6,VEGFA,IL10,EDN1,IL4 |
| GO:MF | growth factor activity | GO:0008083 | 2.24E-09 | 8.649582722 | NGF,CXCL12,IL6,VEGFA,IL10,EGF,IL4 |
| GO:MF | cytokine receptor binding | GO:0005126 | 8.73E-08 | 7.0589776 | NGF,CXCL12,TNF,IL6,VEGFA,IL10,IL4 |
| GO:MF | molecular function regulator | GO:0098772 | 1.20E-07 | 6.921841239 | NGF,ADIPOQ,CXCL12,TNF,IL6,VEGFA,IL10,EDN1,EGF,IL4,ESR1,AKT1,TP53 |
| GO:MF | signaling receptor binding | GO:0005102 | 1.09E-06 | 5.962658295 | NGF,ADIPOQ,CXCL12,TNF,IL6,VEGFA,IL10,EDN1,EGF,IL4,TP53,GJA1,ESR2,AGT |
| GO:MF | growth factor receptor binding | GO:0070851 | 9.46E-06 | 5.02400619 | IL6,VEGFA,IL10,EGF,IL4 |
| GO:MF | antioxidant activity | GO:0016209 | 4.29E-05 | 4.367665485 | ALB,PTGS2,CAT,SOD1,MPO |
| GO:MF | identical protein binding | GO:0042802 | 0.000164759 | 3.783149722 | ADIPOQ,ALB,TNF,VEGFA,MAPK3,PTGS2,ESR1,AKT1,CAT,TP53,SIRT1 |
| GO:MF | heme binding | GO:0020037 | 0.000534622 | 3.271952817 | NOS3,PTGS2,CAT,CYBB,MPO |
| GO:MF | tetrapyrrole binding | GO:0046906 | 0.000749205 | 3.125399504 | NOS3,PTGS2,CAT,CYBB,MPO |
| GO:MF | beta-catenin binding | GO:0008013 | 0.000931595 | 3.030772902 | ESR1,CTNNB1,GJA1,EP300 |
| GO:MF | enzyme binding | GO:0019899 | 0.001422059 | 2.847082241 | TNF,MAPK3,PTGS2,ESR1,AKT1,CAT,TP53,SIRT1,CTNNB1,GJA1,ESR2,SOD1 |
| GO:MF | estrogen receptor activity | GO:0030284 | 0.001586591 | 2.799535067 | ESR1,ESR2 |
| GO:MF | carboxylic acid binding | GO:0031406 | 0.001759806 | 2.754535119 | ADIPOQ,NOS3,ALB |
| GO:MF | estrogen response element binding | GO:0034056 | 0.003170664 | 2.498849797 | ESR1,ESR2 |
| GO:MF | nitric-oxide synthase regulator activity | GO:0030235 | 0.004094495 | 2.387799699 | ESR1,AKT1 |
| GO:MF | nuclear receptor binding | GO:0016922 | 0.006226626 | 2.205747236 | ESR1,SIRT1,CTNNB1,EP300 |
| GO:MF | protein dimerization activity | GO:0046983 | 0.007288335 | 2.137371694 | ADIPOQ,VEGFA,IL10,PTGS2,AKT1,CAT,TP53,CYBB |
| GO:MF | RNA polymerase II-specific DNA-binding transcription factor binding | GO:0061629 | 0.009669269 | 2.014606362 | ESR1,TP53,SIRT1,CTNNB1,EP300 |
| GO:MF | peroxidase activity | GO:0004601 | 0.017601299 | 1.754455279 | PTGS2,CAT,MPO |
| GO:MF | protein kinase binding | GO:0019901 | 0.021830753 | 1.660931292 | ESR1,AKT1,TP53,SIRT1,CTNNB1,GJA1 |
| GO:MF | oxidoreductase activity, acting on peroxide as acceptor | GO:0016684 | 0.022088287 | 1.655837972 | PTGS2,CAT,MPO |
| GO:MF | p53 binding | GO:0002039 | 0.022347318 | 1.650774595 | TP53,SIRT1,EP300 |
| GO:MF | copper ion binding | GO:0005507 | 0.024219959 | 1.61582659 | ALB,TP53,SOD1 |
| GO:MF | transition metal ion binding | GO:0046914 | 0.024985013 | 1.602320414 | NOS3,ALB,ESR1,TP53,EP300,MMP3,ESR2,SOD1 |
| GO:MF | tumor necrosis factor receptor superfamily binding | GO:0032813 | 0.028549778 | 1.54439726 | NGF,TNF |
| GO:MF | zinc ion binding | GO:0008270 | 0.02980376 | 1.525728945 | ALB,ESR1,TP53,EP300,MMP3,ESR2,SOD1 |
| GO:MF | kinase binding | GO:0019900 | 0.040752923 | 1.389841232 | ESR1,AKT1,TP53,SIRT1,CTNNB1,GJA1 |
| GO:MF | phosphatase binding | GO:0019902 | 0.041386784 | 1.383138321 | MAPK3,TP53,CTNNB1,SOD1 |
| GO:MF | protein binding | GO:0005515 | 0.041647044 | 1.380415817 | NGF,ADIPOQ,NOS3,CXCL12,ALB,TNF,IL6,VEGFA,MAPK3,IL10,PTGS2,EDN1,EGF,IL4,ESR1,AKT1,CAT,TP53,SIRT1,CTNNB1,PIK3CA,GJA1,EP300,CYBB,MMP3,ESR2,SOD1,AGT,MPO |
| GO:MF | DNA-binding transcription factor binding | GO:0140297 | 0.045077477 | 1.346040401 | ESR1,TP53,SIRT1,CTNNB1,EP300 |
| GO:MF | chromatin binding | GO:0003682 | 0.049337318 | 1.306824464 | ESR1,TP53,SIRT1,CTNNB1,EP300,MPO |
| GO:BP | response to endogenous stimulus | GO:0009719 | 1.56E-20 | 19.80756125 | NGF,ADIPOQ,NOS3,CXCL12,TNF,IL6,MAPK3,IL10,PTGS2,EDN1,IL4,ESR1,AKT1,CAT,TP53,SIRT1,CTNNB1,PIK3CA,GJA1,EP300,CYBB,ESR2,SOD1,AGT |
| GO:BP | cellular response to chemical stimulus | GO:0070887 | 9.04E-20 | 19.04385655 | NGF,ADIPOQ,NOS3,CXCL12,ALB,TNF,IL6,VEGFA,MAPK3,IL10,PTGS2,EDN1,IL4,ESR1,AKT1,CAT,TP53,SIRT1,CTNNB1,PIK3CA,GJA1,EP300,CYBB,MMP3,ESR2,SOD1,AGT,MPO |
| GO:BP | cell death | GO:0008219 | 1.45E-18 | 17.83909377 | NGF,ADIPOQ,NOS3,CXCL12,ALB,TNF,IL6,VEGFA,MAPK3,IL10,PTGS2,EDN1,IL4,ESR1,AKT1,CAT,TP53,SIRT1,CTNNB1,PIK3CA,EP300,MMP3,SOD1,AGT,MPO |
| GO:BP | positive regulation of gene expression | GO:0010628 | 2.10E-18 | 17.67797249 | NGF,ADIPOQ,NOS3,TNF,IL6,VEGFA,MAPK3,IL10,PTGS2,EGF,IL4,ESR1,AKT1,TP53,SIRT1,CTNNB1,GJA1,EP300,CYBB,SOD1,AGT |
| GO:BP | apoptotic process | GO:0006915 | 6.04E-18 | 17.21876431 | NGF,ADIPOQ,NOS3,CXCL12,ALB,TNF,IL6,VEGFA,MAPK3,IL10,PTGS2,EDN1,IL4,ESR1,AKT1,CAT,TP53,SIRT1,CTNNB1,PIK3CA,EP300,SOD1,AGT,MPO |
| GO:BP | regulation of cell death | GO:0010941 | 6.09E-18 | 17.21503777 | NGF,ADIPOQ,NOS3,CXCL12,ALB,TNF,IL6,VEGFA,IL10,PTGS2,EDN1,IL4,ESR1,AKT1,CAT,TP53,SIRT1,CTNNB1,PIK3CA,MMP3,SOD1,AGT,MPO |
| GO:BP | response to organic substance | GO:0010033 | 7.77E-18 | 17.10980617 | NGF,ADIPOQ,NOS3,CXCL12,TNF,IL6,VEGFA,MAPK3,IL10,PTGS2,EDN1,IL4,ESR1,AKT1,CAT,TP53,SIRT1,CTNNB1,PIK3CA,GJA1,EP300,CYBB,MMP3,ESR2,SOD1,AGT,MPO |
| GO:BP | response to abiotic stimulus | GO:0009628 | 7.79E-18 | 17.10860682 | ADIPOQ,NOS3,CXCL12,TNF,VEGFA,MAPK3,PTGS2,EDN1,AKT1,CAT,TP53,SIRT1,CTNNB1,PIK3CA,GJA1,EP300,CYBB,MMP3,SOD1,AGT,MPO |
| GO:BP | cellular response to organic substance | GO:0071310 | 8.64E-18 | 17.06365806 | NGF,ADIPOQ,NOS3,CXCL12,TNF,IL6,VEGFA,MAPK3,IL10,PTGS2,EDN1,IL4,ESR1,AKT1,CAT,TP53,SIRT1,CTNNB1,PIK3CA,GJA1,EP300,CYBB,ESR2,SOD1,AGT |
| GO:BP | programmed cell death | GO:0012501 | 1.15E-17 | 16.94015745 | NGF,ADIPOQ,NOS3,CXCL12,ALB,TNF,IL6,VEGFA,MAPK3,IL10,PTGS2,EDN1,IL4,ESR1,AKT1,CAT,TP53,SIRT1,CTNNB1,PIK3CA,EP300,SOD1,AGT,MPO |
| GO:BP | response to oxygen-containing compound | GO:1901700 | 1.35E-17 | 16.87080158 | ADIPOQ,NOS3,CXCL12,TNF,IL6,MAPK3,IL10,PTGS2,EDN1,ESR1,AKT1,CAT,TP53,SIRT1,CTNNB1,PIK3CA,GJA1,CYBB,MMP3,ESR2,SOD1,AGT,MPO |
| GO:BP | regulation of apoptotic process | GO:0042981 | 1.50E-17 | 16.82430364 | NGF,ADIPOQ,NOS3,CXCL12,ALB,TNF,IL6,VEGFA,IL10,PTGS2,EDN1,IL4,ESR1,AKT1,CAT,TP53,SIRT1,CTNNB1,PIK3CA |
| GO:BP | cellular response to oxygen-containing compound | GO:1901701 | 1.70E-17 | 16.76941811 | ADIPOQ,NOS3,TNF,IL6,MAPK3,IL10,PTGS2,EDN1,ESR1,AKT1,TP53,SIRT1,CTNNB1,PIK3CA,GJA1,CYBB,MMP3,ESR2,SOD1,AGT,MPO |
| GO:BP | regulation of programmed cell death | GO:0043067 | 2.18E-17 | 16.66230291 | NGF,ADIPOQ,NOS3,CXCL12,ALB,TNF,IL6,VEGFA,IL10,PTGS2,EDN1,IL4,ESR1,AKT1,CAT,TP53,SIRT1,CTNNB1,PIK3CA |
| GO:BP | negative regulation of apoptotic process | GO:0043066 | 2.21E-17 | 16.65464936 | NGF,NOS3,CXCL12,ALB,TNF,IL6,VEGFA,IL10,PTGS2,EDN1,IL4,AKT1,CAT,TP53,SIRT1,CTNNB1,PIK3CA |
| GO:BP | regulation of cell population proliferation | GO:0042127 | 2.43E-17 | 16.6147345 | NGF,ADIPOQ,NOS3,CXCL12,TNF,IL6,VEGFA,MAPK3,IL10,PTGS2,EDN1,EGF,IL4,ESR1,AKT1,TP53,SIRT1,CTNNB1,PIK3CA,GJA1 |
| GO:BP | negative regulation of programmed cell death | GO:0043069 | 3.08E-17 | 16.5111719 | NGF,NOS3,CXCL12,ALB,TNF,IL6,VEGFA,IL10,PTGS2,EDN1,IL4,AKT1,CAT,TP53,SIRT1,CTNNB1,PIK3CA |
| GO:BP | response to external stimulus | GO:0009605 | 4.30E-17 | 16.3665011 | ADIPOQ,NOS3,CXCL12,ALB,TNF,IL6,VEGFA,MAPK3,IL10,PTGS2,EDN1,IL4,ESR1,AKT1,CAT,TP53,SIRT1,CTNNB1,PIK3CA,GJA1,EP300,CYBB,MMP3,SOD1,AGT,MPO |
| GO:BP | cellular response to chemical stress | GO:0062197 | 4.92E-17 | 16.30846682 | NOS3,TNF,IL6,MAPK3,IL10,PTGS2,EDN1,AKT1,TP53,SIRT1,CTNNB1,PIK3CA,MMP3,SOD1,MPO |
| GO:BP | response to oxidative stress | GO:0006979 | 5.17E-17 | 16.28631774 | ADIPOQ,NOS3,TNF,IL6,MAPK3,IL10,PTGS2,EDN1,AKT1,CAT,TP53,SIRT1,CTNNB1,MMP3,SOD1,MPO |
| GO:BP | response to lipid | GO:0033993 | 1.50E-16 | 15.82413334 | ADIPOQ,NOS3,TNF,IL6,MAPK3,IL10,PTGS2,EDN1,ESR1,AKT1,CAT,SIRT1,CTNNB1,PIK3CA,EP300,CYBB,ESR2,AGT,MPO |
| GO:BP | negative regulation of cell death | GO:0060548 | 1.55E-16 | 15.81080357 | NGF,NOS3,CXCL12,ALB,TNF,IL6,VEGFA,IL10,PTGS2,EDN1,IL4,AKT1,CAT,TP53,SIRT1,CTNNB1,PIK3CA |
| GO:BP | regulation of smooth muscle cell proliferation | GO:0048660 | 1.62E-16 | 15.78991262 | ADIPOQ,NOS3,TNF,IL6,IL10,PTGS2,EDN1,AKT1,CTNNB1,PIK3CA,GJA1,AGT |
| GO:BP | smooth muscle cell proliferation | GO:0048659 | 2.13E-16 | 15.67230294 | ADIPOQ,NOS3,TNF,IL6,IL10,PTGS2,EDN1,AKT1,CTNNB1,PIK3CA,GJA1,AGT |
| GO:BP | response to organonitrogen compound | GO:0010243 | 5.21E-16 | 15.28280315 | ADIPOQ,CXCL12,TNF,IL6,MAPK3,IL10,PTGS2,EDN1,AKT1,CAT,TP53,SIRT1,CTNNB1,PIK3CA,GJA1,CYBB,MMP3,SOD1,AGT |
| GO:BP | tube development | GO:0035295 | 5.56E-16 | 15.25527579 | NOS3,TNF,IL6,VEGFA,MAPK3,IL10,PTGS2,EDN1,EGF,ESR1,AKT1,CAT,SIRT1,CTNNB1,PIK3CA,GJA1,EP300,CYBB |
| GO:BP | cell population proliferation | GO:0008283 | 6.27E-16 | 15.20275302 | NGF,ADIPOQ,NOS3,CXCL12,TNF,IL6,VEGFA,MAPK3,IL10,PTGS2,EDN1,EGF,IL4,ESR1,AKT1,TP53,SIRT1,CTNNB1,PIK3CA,GJA1 |
| GO:BP | positive regulation of molecular function | GO:0044093 | 8.35E-16 | 15.07817949 | NGF,ADIPOQ,NOS3,TNF,IL6,VEGFA,MAPK3,IL10,EDN1,EGF,IL4,ESR1,AKT1,CAT,SIRT1,CTNNB1,PIK3CA,EP300,ESR2,SOD1,AGT |
| GO:BP | regulation of response to stress | GO:0080134 | 1.32E-15 | 14.87983061 | ADIPOQ,NOS3,CXCL12,TNF,IL6,VEGFA,MAPK3,IL10,PTGS2,EDN1,IL4,ESR1,AKT1,TP53,SIRT1,CTNNB1,EP300,MMP3,SOD1,AGT |
| GO:BP | regulation of signal transduction | GO:0009966 | 1.38E-15 | 14.85955273 | NGF,ADIPOQ,NOS3,CXCL12,TNF,IL6,VEGFA,MAPK3,IL10,PTGS2,EDN1,EGF,IL4,ESR1,AKT1,CAT,TP53,SIRT1,CTNNB1,PIK3CA,GJA1,EP300,ESR2,SOD1,AGT |
| GO:BP | anatomical structure formation involved in morphogenesis | GO:0048646 | 1.94E-15 | 14.71251346 | NOS3,TNF,IL6,VEGFA,MAPK3,IL10,PTGS2,EDN1,EGF,IL4,AKT1,TP53,SIRT1,CTNNB1,PIK3CA,GJA1,EP300,CYBB |
| GO:BP | response to chemical | GO:0042221 | 2.15E-15 | 14.66809851 | NGF,ADIPOQ,NOS3,CXCL12,ALB,TNF,IL6,VEGFA,MAPK3,IL10,PTGS2,EDN1,IL4,ESR1,AKT1,CAT,TP53,SIRT1,CTNNB1,PIK3CA,GJA1,EP300,CYBB,MMP3,ESR2,SOD1,AGT,MPO |
| GO:BP | response to nitrogen compound | GO:1901698 | 2.48E-15 | 14.605208 | ADIPOQ,CXCL12,TNF,IL6,MAPK3,IL10,PTGS2,EDN1,AKT1,CAT,TP53,SIRT1,CTNNB1,PIK3CA,GJA1,CYBB,MMP3,SOD1,AGT |
| GO:BP | anatomical structure morphogenesis | GO:0009653 | 7.01E-15 | 14.15421898 | NGF,ADIPOQ,NOS3,CXCL12,TNF,IL6,VEGFA,MAPK3,IL10,PTGS2,EDN1,EGF,IL4,ESR1,AKT1,TP53,SIRT1,CTNNB1,PIK3CA,GJA1,EP300,CYBB,SOD1,AGT |
| GO:BP | response to peptide | GO:1901652 | 9.17E-15 | 14.03771109 | ADIPOQ,CXCL12,TNF,IL10,PTGS2,EDN1,AKT1,CAT,TP53,SIRT1,PIK3CA,GJA1,CYBB,MMP3,AGT |
| GO:BP | muscle cell proliferation | GO:0033002 | 9.98E-15 | 14.00095618 | ADIPOQ,NOS3,TNF,IL6,IL10,PTGS2,EDN1,AKT1,CTNNB1,PIK3CA,GJA1,AGT |
| GO:BP | regulation of intracellular signal transduction | GO:1902531 | 1.08E-14 | 13.96846191 | NGF,ADIPOQ,CXCL12,TNF,IL6,VEGFA,MAPK3,PTGS2,EDN1,EGF,ESR1,AKT1,CAT,TP53,SIRT1,CTNNB1,PIK3CA,GJA1,EP300,SOD1,AGT |
| GO:BP | positive regulation of cell communication | GO:0010647 | 1.09E-14 | 13.96317762 | NGF,ADIPOQ,NOS3,TNF,IL6,VEGFA,MAPK3,IL10,PTGS2,EDN1,EGF,IL4,CAT,TP53,SIRT1,CTNNB1,PIK3CA,GJA1,EP300,SOD1,AGT |
| GO:BP | positive regulation of signaling | GO:0023056 | 1.14E-14 | 13.94207283 | NGF,ADIPOQ,NOS3,TNF,IL6,VEGFA,MAPK3,IL10,PTGS2,EDN1,EGF,IL4,CAT,TP53,SIRT1,CTNNB1,PIK3CA,GJA1,EP300,SOD1,AGT |
| GO:BP | positive regulation of developmental process | GO:0051094 | 1.75E-14 | 13.75612317 | NGF,ADIPOQ,NOS3,CXCL12,TNF,IL6,VEGFA,IL10,PTGS2,EDN1,EGF,IL4,AKT1,TP53,SIRT1,CTNNB1,GJA1,CYBB |
| GO:BP | positive regulation of cell population proliferation | GO:0008284 | 1.80E-14 | 13.74466669 | CXCL12,TNF,IL6,VEGFA,MAPK3,IL10,PTGS2,EDN1,EGF,IL4,ESR1,AKT1,SIRT1,CTNNB1,PIK3CA,GJA1 |
| GO:BP | cellular response to oxidative stress | GO:0034599 | 2.25E-14 | 13.64776638 | NOS3,TNF,IL6,MAPK3,IL10,EDN1,AKT1,TP53,SIRT1,CTNNB1,MMP3,SOD1,MPO |
| GO:BP | positive regulation of response to stimulus | GO:0048584 | 2.34E-14 | 13.63158615 | NGF,ADIPOQ,NOS3,CXCL12,TNF,IL6,VEGFA,MAPK3,IL10,PTGS2,EDN1,EGF,IL4,CAT,TP53,SIRT1,CTNNB1,PIK3CA,GJA1,EP300 |
| GO:BP | regulation of response to stimulus | GO:0048583 | 2.72E-14 | 13.56552063 | NGF,ADIPOQ,NOS3,CXCL12,TNF,IL6,VEGFA,MAPK3,IL10,PTGS2,EDN1,EGF,IL4,ESR1,AKT1,CAT,TP53,SIRT1,CTNNB1,PIK3CA,GJA1,EP300,MMP3,ESR2,SOD1,AGT |
| GO:BP | regulation of cell communication | GO:0010646 | 2.86E-14 | 13.54349078 | NGF,ADIPOQ,NOS3,CXCL12,TNF,IL6,VEGFA,MAPK3,IL10,PTGS2,EDN1,EGF,IL4,ESR1,AKT1,CAT,TP53,SIRT1,CTNNB1,PIK3CA,GJA1,EP300,ESR2,SOD1,AGT |
| GO:BP | positive regulation of signal transduction | GO:0009967 | 3.08E-14 | 13.51109104 | NGF,ADIPOQ,NOS3,TNF,IL6,VEGFA,MAPK3,IL10,EDN1,EGF,IL4,CAT,TP53,SIRT1,CTNNB1,PIK3CA,GJA1,EP300,SOD1,AGT |
| GO:BP | regulation of signaling | GO:0023051 | 3.17E-14 | 13.49852314 | NGF,ADIPOQ,NOS3,CXCL12,TNF,IL6,VEGFA,MAPK3,IL10,PTGS2,EDN1,EGF,IL4,ESR1,AKT1,CAT,TP53,SIRT1,CTNNB1,PIK3CA,GJA1,EP300,ESR2,SOD1,AGT |
| GO:BP | response to hormone | GO:0009725 | 3.71E-14 | 13.43076647 | ADIPOQ,NOS3,CXCL12,TNF,IL6,IL10,PTGS2,EDN1,ESR1,AKT1,CAT,SIRT1,PIK3CA,EP300,CYBB,ESR2,AGT |
| GO:BP | circulatory system development | GO:0072359 | 5.38E-14 | 13.26920468 | NOS3,TNF,IL6,VEGFA,MAPK3,IL10,PTGS2,EDN1,EGF,AKT1,TP53,SIRT1,CTNNB1,PIK3CA,GJA1,EP300,CYBB |
| GO:BP | cellular response to endogenous stimulus | GO:0071495 | 7.89E-14 | 13.10271827 | NGF,ADIPOQ,TNF,MAPK3,PTGS2,EDN1,IL4,ESR1,AKT1,TP53,SIRT1,CTNNB1,PIK3CA,GJA1,EP300,CYBB,ESR2,SOD1,AGT |
| GO:BP | regulation of multicellular organismal development | GO:2000026 | 8.47E-14 | 13.07200347 | NGF,ADIPOQ,NOS3,CXCL12,TNF,IL6,VEGFA,IL10,EDN1,EGF,IL4,AKT1,TP53,SIRT1,CTNNB1,GJA1,CYBB,SOD1,AGT |
| GO:BP | regulation of DNA-binding transcription factor activity | GO:0051090 | 1.07E-13 | 12.97202886 | TNF,IL6,VEGFA,MAPK3,IL10,EDN1,ESR1,AKT1,CAT,SIRT1,CTNNB1,EP300,ESR2,AGT |
| GO:BP | response to organic cyclic compound | GO:0014070 | 1.17E-13 | 12.9306236 | ADIPOQ,TNF,IL6,MAPK3,IL10,PTGS2,EDN1,ESR1,CAT,SIRT1,CTNNB1,PIK3CA,EP300,CYBB,ESR2,SOD1,AGT |
| GO:BP | positive regulation of phosphorus metabolic process | GO:0010562 | 1.37E-13 | 12.86323504 | NGF,ADIPOQ,NOS3,TNF,IL6,VEGFA,MAPK3,PTGS2,EDN1,EGF,IL4,AKT1,TP53,SIRT1,PIK3CA |
| GO:BP | positive regulation of phosphate metabolic process | GO:0045937 | 1.37E-13 | 12.86323504 | NGF,ADIPOQ,NOS3,TNF,IL6,VEGFA,MAPK3,PTGS2,EDN1,EGF,IL4,AKT1,TP53,SIRT1,PIK3CA |
| GO:BP | regulation of cellular component organization | GO:0051128 | 1.38E-13 | 12.86126842 | NGF,ADIPOQ,CXCL12,TNF,IL6,VEGFA,MAPK3,IL10,EDN1,EGF,IL4,ESR1,AKT1,TP53,SIRT1,CTNNB1,PIK3CA,GJA1,EP300,MMP3,ESR2,AGT |
| GO:BP | tissue development | GO:0009888 | 1.38E-13 | 12.85928624 | ADIPOQ,NOS3,TNF,IL6,VEGFA,MAPK3,IL10,PTGS2,EDN1,EGF,ESR1,AKT1,CAT,TP53,SIRT1,CTNNB1,PIK3CA,GJA1,EP300,SOD1,AGT |
| GO:BP | regulation of multicellular organismal process | GO:0051239 | 1.38E-13 | 12.85903832 | NGF,ADIPOQ,NOS3,CXCL12,TNF,IL6,VEGFA,MAPK3,IL10,PTGS2,EDN1,EGF,IL4,ESR1,AKT1,TP53,SIRT1,CTNNB1,PIK3CA,GJA1,CYBB,SOD1,AGT |
| GO:BP | regulation of DNA metabolic process | GO:0051052 | 2.11E-13 | 12.67589445 | ADIPOQ,TNF,IL6,VEGFA,MAPK3,IL10,IL4,AKT1,TP53,SIRT1,CTNNB1,PIK3CA |
| GO:BP | cellular response to stress | GO:0033554 | 2.39E-13 | 12.62095946 | NOS3,CXCL12,ALB,TNF,IL6,VEGFA,MAPK3,IL10,PTGS2,EDN1,AKT1,TP53,SIRT1,CTNNB1,PIK3CA,EP300,CYBB,MMP3,SOD1,AGT,MPO |
| GO:BP | blood vessel morphogenesis | GO:0048514 | 3.29E-13 | 12.48330564 | NOS3,TNF,IL6,VEGFA,IL10,PTGS2,EDN1,EGF,AKT1,SIRT1,CTNNB1,PIK3CA,GJA1,CYBB,AGT |
| GO:BP | positive regulation of multicellular organismal process | GO:0051240 | 3.56E-13 | 12.44798067 | NGF,ADIPOQ,NOS3,CXCL12,TNF,IL6,VEGFA,IL10,PTGS2,EDN1,EGF,IL4,AKT1,SIRT1,CTNNB1,GJA1,CYBB,SOD1,AGT |
| GO:BP | positive regulation of intracellular signal transduction | GO:1902533 | 4.08E-13 | 12.3890959 | NGF,ADIPOQ,TNF,IL6,VEGFA,MAPK3,EDN1,EGF,CAT,TP53,SIRT1,CTNNB1,PIK3CA,GJA1,EP300,SOD1,AGT |
| GO:BP | response to decreased oxygen levels | GO:0036293 | 4.10E-13 | 12.38745498 | ADIPOQ,CXCL12,TNF,VEGFA,PTGS2,EDN1,AKT1,CAT,TP53,SIRT1,EP300,CYBB |
| GO:BP | positive regulation of protein phosphorylation | GO:0001934 | 4.22E-13 | 12.37486576 | NGF,ADIPOQ,TNF,IL6,VEGFA,MAPK3,PTGS2,EDN1,EGF,IL4,AKT1,TP53,SIRT1,PIK3CA |
| GO:BP | positive regulation of protein modification process | GO:0031401 | 5.76E-13 | 12.23950483 | NGF,ADIPOQ,TNF,IL6,VEGFA,MAPK3,PTGS2,EDN1,EGF,IL4,AKT1,TP53,SIRT1,CTNNB1,PIK3CA |
| GO:BP | cell migration | GO:0016477 | 5.82E-13 | 12.23489878 | ADIPOQ,NOS3,CXCL12,TNF,IL6,VEGFA,MAPK3,IL10,PTGS2,EDN1,EGF,IL4,AKT1,SIRT1,CTNNB1,PIK3CA,GJA1 |
| GO:BP | positive regulation of DNA-binding transcription factor activity | GO:0051091 | 5.94E-13 | 12.22616913 | TNF,IL6,VEGFA,IL10,EDN1,ESR1,AKT1,CAT,CTNNB1,EP300,ESR2,AGT |
| GO:BP | regulation of developmental process | GO:0050793 | 6.85E-13 | 12.16432868 | NGF,ADIPOQ,NOS3,CXCL12,TNF,IL6,VEGFA,IL10,PTGS2,EDN1,EGF,IL4,ESR1,AKT1,TP53,SIRT1,CTNNB1,PIK3CA,GJA1,CYBB,SOD1,AGT |
| GO:BP | response to oxygen levels | GO:0070482 | 9.94E-13 | 12.00253819 | ADIPOQ,CXCL12,TNF,VEGFA,PTGS2,EDN1,AKT1,CAT,TP53,SIRT1,EP300,CYBB |
| GO:BP | angiogenesis | GO:0001525 | 1.23E-12 | 11.91132547 | NOS3,TNF,IL6,VEGFA,IL10,PTGS2,EDN1,EGF,AKT1,SIRT1,CTNNB1,PIK3CA,CYBB,AGT |
| GO:BP | positive regulation of smooth muscle cell proliferation | GO:0048661 | 1.37E-12 | 11.86279187 | TNF,IL6,IL10,PTGS2,EDN1,AKT1,PIK3CA,GJA1,AGT |
| GO:BP | tube morphogenesis | GO:0035239 | 1.50E-12 | 11.8249232 | NOS3,TNF,IL6,VEGFA,IL10,PTGS2,EDN1,EGF,ESR1,AKT1,SIRT1,CTNNB1,PIK3CA,GJA1,CYBB,AGT |
| GO:BP | positive regulation of macromolecule metabolic process | GO:0010604 | 1.53E-12 | 11.81558709 | NGF,ADIPOQ,NOS3,TNF,IL6,VEGFA,MAPK3,IL10,PTGS2,EDN1,EGF,IL4,ESR1,AKT1,TP53,SIRT1,CTNNB1,PIK3CA,GJA1,EP300,CYBB,ESR2,SOD1,AGT |
| GO:BP | cellular response to nitrogen compound | GO:1901699 | 1.61E-12 | 11.79312749 | ADIPOQ,TNF,MAPK3,PTGS2,EDN1,AKT1,TP53,SIRT1,CTNNB1,PIK3CA,GJA1,CYBB,MMP3,SOD1,AGT |
| GO:BP | positive regulation of phosphorylation | GO:0042327 | 1.90E-12 | 11.72219432 | NGF,ADIPOQ,TNF,IL6,VEGFA,MAPK3,PTGS2,EDN1,EGF,IL4,AKT1,TP53,SIRT1,PIK3CA |
| GO:BP | intracellular signal transduction | GO:0035556 | 1.91E-12 | 11.71959322 | NGF,ADIPOQ,NOS3,CXCL12,TNF,IL6,VEGFA,MAPK3,PTGS2,EDN1,EGF,ESR1,AKT1,CAT,TP53,SIRT1,CTNNB1,PIK3CA,GJA1,EP300 |
| GO:BP | blood vessel development | GO:0001568 | 2.27E-12 | 11.64353059 | NOS3,TNF,IL6,VEGFA,IL10,PTGS2,EDN1,EGF,AKT1,SIRT1,CTNNB1,PIK3CA,GJA1,CYBB,AGT |
| GO:BP | locomotion | GO:0040011 | 2.73E-12 | 11.56386159 | ADIPOQ,NOS3,CXCL12,TNF,IL6,VEGFA,MAPK3,IL10,PTGS2,EDN1,EGF,IL4,AKT1,SIRT1,CTNNB1,PIK3CA,GJA1,EP300 |
| GO:BP | response to steroid hormone | GO:0048545 | 2.89E-12 | 11.5398353 | ADIPOQ,TNF,IL6,IL10,PTGS2,EDN1,ESR1,SIRT1,PIK3CA,EP300,CYBB,ESR2 |
| GO:BP | regulation of molecular function | GO:0065009 | 3.16E-12 | 11.50036155 | NGF,ADIPOQ,NOS3,TNF,IL6,VEGFA,MAPK3,IL10,PTGS2,EDN1,EGF,IL4,ESR1,AKT1,CAT,TP53,SIRT1,CTNNB1,PIK3CA,EP300,ESR2,SOD1,AGT |
| GO:BP | gland development | GO:0048732 | 3.29E-12 | 11.48228446 | TNF,IL6,VEGFA,MAPK3,IL10,EDN1,EGF,ESR1,AKT1,CTNNB1,PIK3CA,GJA1,SOD1 |
| GO:BP | response to inorganic substance | GO:0010035 | 3.83E-12 | 11.41734646 | NOS3,IL6,MAPK3,IL10,PTGS2,EDN1,AKT1,CAT,SIRT1,PIK3CA,CYBB,MMP3,SOD1,MPO |
| GO:BP | positive regulation of cell migration | GO:0030335 | 3.89E-12 | 11.41057493 | NOS3,CXCL12,TNF,IL6,VEGFA,MAPK3,PTGS2,EDN1,EGF,IL4,AKT1,SIRT1 |
| GO:BP | vasculature development | GO:0001944 | 4.33E-12 | 11.36364106 | NOS3,TNF,IL6,VEGFA,IL10,PTGS2,EDN1,EGF,AKT1,SIRT1,CTNNB1,PIK3CA,GJA1,CYBB,AGT |
| GO:BP | response to stress | GO:0006950 | 4.35E-12 | 11.36154557 | ADIPOQ,NOS3,CXCL12,ALB,TNF,IL6,VEGFA,MAPK3,IL10,PTGS2,EDN1,IL4,ESR1,AKT1,CAT,TP53,SIRT1,CTNNB1,PIK3CA,EP300,CYBB,MMP3,SOD1,AGT,MPO |
| GO:BP | localization of cell | GO:0051674 | 4.58E-12 | 11.33892634 | ADIPOQ,NOS3,CXCL12,TNF,IL6,VEGFA,MAPK3,IL10,PTGS2,EDN1,EGF,IL4,AKT1,SIRT1,CTNNB1,PIK3CA,GJA1 |
| GO:BP | cell motility | GO:0048870 | 4.58E-12 | 11.33892634 | ADIPOQ,NOS3,CXCL12,TNF,IL6,VEGFA,MAPK3,IL10,PTGS2,EDN1,EGF,IL4,AKT1,SIRT1,CTNNB1,PIK3CA,GJA1 |
| GO:BP | regulation of phosphate metabolic process | GO:0019220 | 4.84E-12 | 11.31507089 | NGF,ADIPOQ,NOS3,TNF,IL6,VEGFA,MAPK3,PTGS2,EDN1,EGF,IL4,AKT1,TP53,SIRT1,PIK3CA,EP300,SOD1,AGT |
| GO:BP | regulation of phosphorus metabolic process | GO:0051174 | 4.97E-12 | 11.30398684 | NGF,ADIPOQ,NOS3,TNF,IL6,VEGFA,MAPK3,PTGS2,EDN1,EGF,IL4,AKT1,TP53,SIRT1,PIK3CA,EP300,SOD1,AGT |
| GO:BP | regulation of response to external stimulus | GO:0032101 | 5.92E-12 | 11.22786528 | ADIPOQ,NOS3,CXCL12,TNF,IL6,VEGFA,MAPK3,IL10,PTGS2,EDN1,IL4,ESR1,EP300,MMP3,SOD1,AGT |
| GO:BP | positive regulation of cell motility | GO:2000147 | 6.64E-12 | 11.17803075 | NOS3,CXCL12,TNF,IL6,VEGFA,MAPK3,PTGS2,EDN1,EGF,IL4,AKT1,SIRT1 |
| GO:BP | positive regulation of cellular component movement | GO:0051272 | 8.78E-12 | 11.0563908 | NOS3,CXCL12,TNF,IL6,VEGFA,MAPK3,PTGS2,EDN1,EGF,IL4,AKT1,SIRT1 |
| GO:BP | positive regulation of locomotion | GO:0040017 | 9.36E-12 | 11.02873052 | NOS3,CXCL12,TNF,IL6,VEGFA,MAPK3,PTGS2,EDN1,EGF,IL4,AKT1,SIRT1 |
| GO:BP | positive regulation of cell differentiation | GO:0045597 | 9.56E-12 | 11.01956224 | NGF,ADIPOQ,CXCL12,TNF,IL6,VEGFA,IL10,PTGS2,EDN1,IL4,AKT1,SIRT1,CTNNB1,GJA1 |
| GO:BP | positive regulation of metabolic process | GO:0009893 | 1.11E-11 | 10.95416169 | NGF,ADIPOQ,NOS3,TNF,IL6,VEGFA,MAPK3,IL10,PTGS2,EDN1,EGF,IL4,ESR1,AKT1,TP53,SIRT1,CTNNB1,PIK3CA,GJA1,EP300,CYBB,ESR2,SOD1,AGT |
| GO:BP | animal organ development | GO:0048513 | 1.30E-11 | 10.88543815 | ADIPOQ,NOS3,CXCL12,TNF,IL6,VEGFA,MAPK3,IL10,PTGS2,EDN1,EGF,IL4,ESR1,AKT1,CAT,TP53,SIRT1,CTNNB1,PIK3CA,GJA1,EP300 |
| GO:BP | cellular response to organonitrogen compound | GO:0071417 | 1.59E-11 | 10.79896764 | ADIPOQ,TNF,MAPK3,PTGS2,EDN1,AKT1,TP53,SIRT1,CTNNB1,PIK3CA,GJA1,CYBB,SOD1,AGT |
| GO:BP | homeostatic process | GO:0042592 | 1.61E-11 | 10.79252099 | ADIPOQ,NOS3,CXCL12,ALB,TNF,IL6,VEGFA,MAPK3,PTGS2,EDN1,IL4,ESR1,AKT1,SIRT1,CTNNB1,PIK3CA,GJA1 |
| GO:BP | response to hypoxia | GO:0001666 | 1.63E-11 | 10.78869412 | ADIPOQ,CXCL12,TNF,VEGFA,PTGS2,EDN1,CAT,TP53,SIRT1,EP300,CYBB |
| GO:BP | regulation of phosphorylation | GO:0042325 | 1.86E-11 | 10.72969986 | NGF,ADIPOQ,TNF,IL6,VEGFA,MAPK3,PTGS2,EDN1,EGF,IL4,AKT1,TP53,SIRT1,PIK3CA,EP300,SOD1,AGT |
| GO:BP | regulation of protein modification process | GO:0031399 | 1.86E-11 | 10.72936353 | NGF,ADIPOQ,TNF,IL6,VEGFA,MAPK3,PTGS2,EDN1,EGF,IL4,AKT1,TP53,SIRT1,CTNNB1,PIK3CA,EP300,SOD1,AGT |
| GO:BP | regulation of cell migration | GO:0030334 | 2.37E-11 | 10.62508195 | ADIPOQ,NOS3,CXCL12,TNF,IL6,VEGFA,MAPK3,PTGS2,EDN1,EGF,IL4,AKT1,SIRT1,GJA1 |
| GO:BP | positive regulation of cellular component organization | GO:0051130 | 2.66E-11 | 10.57438721 | NGF,CXCL12,TNF,IL6,VEGFA,MAPK3,EDN1,EGF,IL4,ESR1,TP53,CTNNB1,PIK3CA,EP300,MMP3,AGT |
| GO:BP | response to activity | GO:0014823 | 3.20E-11 | 10.49429176 | ADIPOQ,TNF,IL6,IL10,EDN1,CAT,PIK3CA,AGT |
| GO:BP | cellular response to growth factor stimulus | GO:0071363 | 3.31E-11 | 10.48043024 | NGF,VEGFA,MAPK3,IL10,EDN1,IL4,AKT1,CAT,TP53,SIRT1,CTNNB1,PIK3CA,EP300,AGT |
| GO:BP | system development | GO:0048731 | 5.23E-11 | 10.28173726 | NGF,ADIPOQ,NOS3,CXCL12,TNF,IL6,VEGFA,MAPK3,IL10,PTGS2,EDN1,EGF,IL4,ESR1,AKT1,CAT,TP53,SIRT1,CTNNB1,PIK3CA,GJA1,EP300,CYBB |
| GO:BP | regulation of biological quality | GO:0065008 | 5.34E-11 | 10.27239442 | NGF,ADIPOQ,NOS3,CXCL12,ALB,TNF,IL6,VEGFA,MAPK3,IL10,PTGS2,EDN1,IL4,ESR1,AKT1,TP53,SIRT1,CTNNB1,PIK3CA,GJA1,EP300 |
| GO:BP | regulation of cell motility | GO:2000145 | 5.71E-11 | 10.24321838 | ADIPOQ,NOS3,CXCL12,TNF,IL6,VEGFA,MAPK3,PTGS2,EDN1,EGF,IL4,AKT1,SIRT1,GJA1 |
| GO:BP | response to growth factor | GO:0070848 | 5.76E-11 | 10.23952787 | NGF,VEGFA,MAPK3,IL10,EDN1,IL4,AKT1,CAT,TP53,SIRT1,CTNNB1,PIK3CA,EP300,AGT |
| GO:BP | regulation of protein phosphorylation | GO:0001932 | 6.02E-11 | 10.22060522 | NGF,ADIPOQ,TNF,IL6,VEGFA,MAPK3,PTGS2,EDN1,EGF,IL4,AKT1,TP53,SIRT1,PIK3CA,SOD1,AGT |
| GO:BP | negative regulation of cellular process | GO:0048523 | 6.76E-11 | 10.16990134 | NGF,ADIPOQ,NOS3,CXCL12,ALB,TNF,IL6,VEGFA,IL10,PTGS2,EDN1,IL4,ESR1,AKT1,CAT,TP53,SIRT1,CTNNB1,PIK3CA,GJA1,EP300,MMP3,ESR2,SOD1,AGT,MPO |
| GO:BP | positive regulation of cellular protein metabolic process | GO:0032270 | 9.10E-11 | 10.04082252 | NGF,ADIPOQ,TNF,IL6,VEGFA,MAPK3,PTGS2,EDN1,EGF,IL4,AKT1,TP53,SIRT1,CTNNB1,PIK3CA |
| GO:BP | epithelial cell migration | GO:0010631 | 9.53E-11 | 10.02073783 | NOS3,TNF,VEGFA,PTGS2,EDN1,EGF,IL4,AKT1,SIRT1,PIK3CA,AGT |
| GO:BP | cellular response to stimulus | GO:0051716 | 9.77E-11 | 10.01009643 | NGF,ADIPOQ,NOS3,CXCL12,ALB,TNF,IL6,VEGFA,MAPK3,IL10,PTGS2,EDN1,EGF,IL4,ESR1,AKT1,CAT,TP53,SIRT1,CTNNB1,PIK3CA,GJA1,EP300,CYBB,MMP3,ESR2,SOD1,AGT,MPO |
| GO:BP | positive regulation of biological process | GO:0048518 | 1.04E-10 | 9.984365236 | NGF,ADIPOQ,NOS3,CXCL12,TNF,IL6,VEGFA,MAPK3,IL10,PTGS2,EDN1,EGF,IL4,ESR1,AKT1,CAT,TP53,SIRT1,CTNNB1,PIK3CA,GJA1,EP300,CYBB,MMP3,ESR2,SOD1,AGT |
| GO:BP | regulation of locomotion | GO:0040012 | 1.06E-10 | 9.974022349 | ADIPOQ,NOS3,CXCL12,TNF,IL6,VEGFA,MAPK3,PTGS2,EDN1,EGF,IL4,AKT1,SIRT1,GJA1 |
| GO:BP | epithelium migration | GO:0090132 | 1.07E-10 | 9.972031736 | NOS3,TNF,VEGFA,PTGS2,EDN1,EGF,IL4,AKT1,SIRT1,PIK3CA,AGT |
| GO:BP | response to reactive oxygen species | GO:0000302 | 1.09E-10 | 9.964086526 | NOS3,IL6,MAPK3,IL10,EDN1,AKT1,CAT,SIRT1,SOD1,MPO |
| GO:BP | ameboidal-type cell migration | GO:0001667 | 1.25E-10 | 9.902233512 | NOS3,TNF,VEGFA,PTGS2,EDN1,EGF,IL4,AKT1,SIRT1,PIK3CA,GJA1,AGT |
| GO:BP | tissue migration | GO:0090130 | 1.33E-10 | 9.876143816 | NOS3,TNF,VEGFA,PTGS2,EDN1,EGF,IL4,AKT1,SIRT1,PIK3CA,AGT |
| GO:BP | regulation of anatomical structure morphogenesis | GO:0022603 | 1.59E-10 | 9.799352485 | NGF,NOS3,CXCL12,TNF,IL6,VEGFA,IL10,EDN1,EGF,ESR1,SIRT1,CTNNB1,GJA1,CYBB,AGT |
| GO:BP | regulation of cellular component movement | GO:0051270 | 1.64E-10 | 9.785855434 | ADIPOQ,NOS3,CXCL12,TNF,IL6,VEGFA,MAPK3,PTGS2,EDN1,EGF,IL4,AKT1,SIRT1,GJA1 |
| GO:BP | endothelial cell migration | GO:0043542 | 1.93E-10 | 9.715237088 | NOS3,TNF,VEGFA,PTGS2,EDN1,EGF,AKT1,SIRT1,PIK3CA,AGT |
| GO:BP | movement of cell or subcellular component | GO:0006928 | 2.16E-10 | 9.666128332 | ADIPOQ,NOS3,CXCL12,TNF,IL6,VEGFA,MAPK3,IL10,PTGS2,EDN1,EGF,IL4,AKT1,SIRT1,CTNNB1,PIK3CA,GJA1 |
| GO:BP | positive regulation of cellular metabolic process | GO:0031325 | 2.17E-10 | 9.663346652 | NGF,ADIPOQ,NOS3,TNF,IL6,VEGFA,MAPK3,IL10,PTGS2,EDN1,EGF,IL4,ESR1,AKT1,TP53,SIRT1,CTNNB1,PIK3CA,EP300,ESR2,SOD1,AGT |
| GO:BP | positive regulation of protein metabolic process | GO:0051247 | 2.26E-10 | 9.645907334 | NGF,ADIPOQ,TNF,IL6,VEGFA,MAPK3,PTGS2,EDN1,EGF,IL4,AKT1,TP53,SIRT1,CTNNB1,PIK3CA |
| GO:BP | negative regulation of cell communication | GO:0010648 | 2.31E-10 | 9.636050037 | ADIPOQ,NOS3,CXCL12,TNF,IL6,IL10,PTGS2,EDN1,IL4,ESR1,AKT1,TP53,SIRT1,CTNNB1,GJA1 |
| GO:BP | growth | GO:0040007 | 2.34E-10 | 9.631056634 | NGF,CXCL12,VEGFA,EDN1,ESR1,AKT1,TP53,SIRT1,CTNNB1,PIK3CA,GJA1,EP300,ESR2,SOD1,AGT |
| GO:BP | multicellular organism development | GO:0007275 | 2.39E-10 | 9.622384981 | NGF,ADIPOQ,NOS3,CXCL12,TNF,IL6,VEGFA,MAPK3,IL10,PTGS2,EDN1,EGF,IL4,ESR1,AKT1,CAT,TP53,SIRT1,CTNNB1,PIK3CA,GJA1,EP300,CYBB |
| GO:BP | negative regulation of signaling | GO:0023057 | 2.39E-10 | 9.621913653 | ADIPOQ,NOS3,CXCL12,TNF,IL6,IL10,PTGS2,EDN1,IL4,ESR1,AKT1,TP53,SIRT1,CTNNB1,GJA1 |
| GO:BP | regulation of epithelial cell migration | GO:0010632 | 2.45E-10 | 9.610861169 | NOS3,TNF,VEGFA,PTGS2,EDN1,EGF,IL4,AKT1,SIRT1 |
| GO:BP | peptidyl-amino acid modification | GO:0018193 | 2.56E-10 | 9.59185995 | NGF,ADIPOQ,TNF,IL6,VEGFA,MAPK3,PTGS2,EGF,IL4,AKT1,TP53,SIRT1,CTNNB1,PIK3CA,EP300 |
| GO:BP | regulation of transport | GO:0051049 | 2.58E-10 | 9.587792966 | ADIPOQ,NOS3,CXCL12,TNF,IL6,VEGFA,MAPK3,PTGS2,EDN1,EGF,IL4,AKT1,SIRT1,CTNNB1,GJA1,CYBB,SOD1,AGT |
| GO:BP | cellular response to reactive oxygen species | GO:0034614 | 2.77E-10 | 9.557045314 | NOS3,IL6,MAPK3,IL10,EDN1,AKT1,SIRT1,SOD1,MPO |
| GO:BP | phosphatidylinositol 3-kinase signaling | GO:0014065 | 2.84E-10 | 9.546868969 | TNF,VEGFA,EDN1,EGF,AKT1,CAT,SIRT1,PIK3CA,AGT |
| GO:BP | apoptotic signaling pathway | GO:0097190 | 2.84E-10 | 9.546686092 | NGF,NOS3,CXCL12,TNF,PTGS2,IL4,AKT1,TP53,SIRT1,CTNNB1,EP300,SOD1,AGT |
| GO:BP | positive regulation of cellular process | GO:0048522 | 3.44E-10 | 9.462906771 | NGF,ADIPOQ,NOS3,CXCL12,TNF,IL6,VEGFA,MAPK3,IL10,PTGS2,EDN1,EGF,IL4,ESR1,AKT1,CAT,TP53,SIRT1,CTNNB1,PIK3CA,GJA1,EP300,MMP3,ESR2,SOD1,AGT |
| GO:BP | negative regulation of biological process | GO:0048519 | 3.60E-10 | 9.44328355 | NGF,ADIPOQ,NOS3,CXCL12,ALB,TNF,IL6,VEGFA,IL10,PTGS2,EDN1,EGF,IL4,ESR1,AKT1,CAT,TP53,SIRT1,CTNNB1,PIK3CA,GJA1,EP300,MMP3,ESR2,SOD1,AGT,MPO |
| GO:BP | negative regulation of molecular function | GO:0044092 | 3.66E-10 | 9.436616175 | NGF,ADIPOQ,NOS3,TNF,VEGFA,MAPK3,IL10,PTGS2,ESR1,AKT1,CAT,TP53,SIRT1 |
| GO:BP | reactive oxygen species metabolic process | GO:0072593 | 4.03E-10 | 9.395193001 | NOS3,TNF,EDN1,CAT,TP53,CYBB,MMP3,SOD1,AGT,MPO |
| GO:BP | defense response | GO:0006952 | 4.55E-10 | 9.341563536 | ADIPOQ,CXCL12,TNF,IL6,MAPK3,IL10,PTGS2,EDN1,IL4,ESR1,AKT1,TP53,EP300,CYBB,MMP3,SOD1,AGT,MPO |
| GO:BP | regulation of cellular response to stress | GO:0080135 | 4.83E-10 | 9.315972502 | CXCL12,TNF,VEGFA,MAPK3,IL10,PTGS2,AKT1,TP53,SIRT1,CTNNB1,EP300,MMP3,SOD1 |
| GO:BP | cell communication | GO:0007154 | 5.01E-10 | 9.3003188 | NGF,ADIPOQ,NOS3,CXCL12,ALB,TNF,IL6,VEGFA,MAPK3,IL10,PTGS2,EDN1,EGF,IL4,ESR1,AKT1,CAT,TP53,SIRT1,CTNNB1,PIK3CA,GJA1,EP300,CYBB,ESR2,SOD1,AGT |
| GO:BP | response to nutrient levels | GO:0031667 | 5.04E-10 | 9.297380086 | ADIPOQ,ALB,TNF,MAPK3,PTGS2,AKT1,CAT,TP53,SIRT1,CYBB,SOD1,MPO |
| GO:BP | developmental growth | GO:0048589 | 6.26E-10 | 9.203743229 | NGF,CXCL12,VEGFA,EDN1,ESR1,AKT1,TP53,CTNNB1,PIK3CA,GJA1,EP300,SOD1,AGT |
| GO:BP | positive regulation of nitrogen compound metabolic process | GO:0051173 | 6.30E-10 | 9.200820761 | NGF,ADIPOQ,NOS3,TNF,IL6,VEGFA,MAPK3,IL10,PTGS2,EDN1,EGF,IL4,ESR1,AKT1,TP53,SIRT1,CTNNB1,PIK3CA,EP300 |
| GO:BP | regulation of cell differentiation | GO:0045595 | 8.21E-10 | 9.08586118 | NGF,ADIPOQ,CXCL12,TNF,IL6,VEGFA,IL10,PTGS2,EDN1,IL4,AKT1,TP53,SIRT1,CTNNB1,GJA1,SOD1,AGT |
| GO:BP | regulation of apoptotic signaling pathway | GO:2001233 | 9.12E-10 | 9.040150072 | NOS3,CXCL12,TNF,PTGS2,IL4,AKT1,TP53,SIRT1,CTNNB1,SOD1,AGT |
| GO:BP | regulation of endothelial cell migration | GO:0010594 | 1.10E-09 | 8.960329337 | NOS3,TNF,VEGFA,PTGS2,EDN1,EGF,AKT1,SIRT1,AGT |
| GO:BP | response to extracellular stimulus | GO:0009991 | 1.14E-09 | 8.944053324 | ADIPOQ,ALB,TNF,MAPK3,PTGS2,AKT1,CAT,TP53,SIRT1,CYBB,SOD1,MPO |
| GO:BP | multicellular organismal homeostasis | GO:0048871 | 1.17E-09 | 8.932216741 | ADIPOQ,NOS3,ALB,TNF,IL6,VEGFA,PTGS2,IL4,CTNNB1,PIK3CA,GJA1,SOD1 |
| GO:BP | regulation of immune system process | GO:0002682 | 1.39E-09 | 8.857122964 | ADIPOQ,CXCL12,TNF,IL6,VEGFA,MAPK3,IL10,EDN1,IL4,ESR1,AKT1,SIRT1,CTNNB1,PIK3CA,EP300 |
| GO:BP | positive regulation of endothelial cell migration | GO:0010595 | 1.50E-09 | 8.822517606 | NOS3,VEGFA,PTGS2,EDN1,EGF,AKT1,SIRT1,AGT |
| GO:BP | epithelial cell proliferation | GO:0050673 | 1.54E-09 | 8.811343219 | CXCL12,TNF,IL6,VEGFA,IL10,EGF,ESR1,AKT1,SIRT1,CTNNB1 |
| GO:BP | regulation of transferase activity | GO:0051338 | 1.57E-09 | 8.803824406 | ADIPOQ,TNF,VEGFA,MAPK3,EDN1,EGF,IL4,AKT1,TP53,SIRT1,CTNNB1,PIK3CA,SOD1,AGT |
| GO:BP | phosphatidylinositol-mediated signaling | GO:0048015 | 1.68E-09 | 8.77585156 | TNF,VEGFA,EDN1,EGF,AKT1,CAT,SIRT1,PIK3CA,AGT |
| GO:BP | signal transduction | GO:0007165 | 1.72E-09 | 8.765054547 | NGF,ADIPOQ,NOS3,CXCL12,TNF,IL6,VEGFA,MAPK3,IL10,PTGS2,EDN1,EGF,IL4,ESR1,AKT1,CAT,TP53,SIRT1,CTNNB1,PIK3CA,GJA1,EP300,CYBB,ESR2,SOD1,AGT |
| GO:BP | positive regulation of transferase activity | GO:0051347 | 1.87E-09 | 8.727958344 | ADIPOQ,TNF,VEGFA,MAPK3,EDN1,EGF,IL4,AKT1,SIRT1,CTNNB1,PIK3CA |
| GO:BP | positive regulation of catalytic activity | GO:0043085 | 2.03E-09 | 8.692794529 | ADIPOQ,NOS3,TNF,VEGFA,MAPK3,EDN1,EGF,IL4,ESR1,AKT1,SIRT1,CTNNB1,PIK3CA,SOD1,AGT |
| GO:BP | inositol lipid-mediated signaling | GO:0048017 | 2.06E-09 | 8.686876073 | TNF,VEGFA,EDN1,EGF,AKT1,CAT,SIRT1,PIK3CA,AGT |
| GO:BP | positive regulation of cellular biosynthetic process | GO:0031328 | 2.10E-09 | 8.677251315 | NOS3,TNF,IL6,VEGFA,MAPK3,IL10,PTGS2,EDN1,EGF,IL4,ESR1,AKT1,TP53,SIRT1,CTNNB1,EP300,ESR2,AGT |
| GO:BP | regulation of leukocyte migration | GO:0002685 | 2.51E-09 | 8.600342517 | CXCL12,TNF,IL6,VEGFA,MAPK3,EDN1,IL4,AKT1 |
| GO:BP | regulation of cellular protein metabolic process | GO:0032268 | 2.55E-09 | 8.592914218 | NGF,ADIPOQ,TNF,IL6,VEGFA,MAPK3,IL10,PTGS2,EDN1,EGF,IL4,AKT1,TP53,SIRT1,CTNNB1,PIK3CA,EP300,SOD1,AGT |
| GO:BP | response to peptide hormone | GO:0043434 | 2.80E-09 | 8.553574924 | ADIPOQ,CXCL12,IL10,PTGS2,EDN1,AKT1,CAT,SIRT1,PIK3CA,CYBB,AGT |
| GO:BP | positive regulation of biosynthetic process | GO:0009891 | 2.81E-09 | 8.552018401 | NOS3,TNF,IL6,VEGFA,MAPK3,IL10,PTGS2,EDN1,EGF,IL4,ESR1,AKT1,TP53,SIRT1,CTNNB1,EP300,ESR2,AGT |
| GO:BP | cellular response to organic cyclic compound | GO:0071407 | 2.82E-09 | 8.549043715 | ADIPOQ,TNF,MAPK3,IL10,PTGS2,EDN1,ESR1,SIRT1,CTNNB1,EP300,ESR2,SOD1 |
| GO:BP | anatomical structure development | GO:0048856 | 3.01E-09 | 8.520864566 | NGF,ADIPOQ,NOS3,CXCL12,TNF,IL6,VEGFA,MAPK3,IL10,PTGS2,EDN1,EGF,IL4,ESR1,AKT1,CAT,TP53,SIRT1,CTNNB1,PIK3CA,GJA1,EP300,CYBB |
| GO:BP | regulation of morphogenesis of an epithelium | GO:1905330 | 3.47E-09 | 8.460109387 | TNF,VEGFA,EGF,ESR1,CTNNB1,GJA1,AGT |
| GO:BP | leukocyte migration | GO:0050900 | 3.50E-09 | 8.45619858 | CXCL12,TNF,IL6,VEGFA,MAPK3,IL10,EDN1,IL4,AKT1 |
| GO:BP | positive regulation of leukocyte migration | GO:0002687 | 3.59E-09 | 8.445092023 | CXCL12,TNF,IL6,VEGFA,MAPK3,EDN1,IL4 |
| GO:BP | regulation of gene expression | GO:0010468 | 3.96E-09 | 8.402495893 | NGF,ADIPOQ,NOS3,TNF,IL6,VEGFA,MAPK3,IL10,PTGS2,EDN1,EGF,IL4,ESR1,AKT1,CAT,TP53,SIRT1,CTNNB1,PIK3CA,GJA1,EP300,CYBB,ESR2,SOD1,AGT |
| GO:BP | epithelium development | GO:0060429 | 4.14E-09 | 8.383258883 | ADIPOQ,TNF,VEGFA,IL10,EDN1,EGF,ESR1,AKT1,CAT,TP53,CTNNB1,GJA1,EP300,SOD1,AGT |
| GO:BP | regulation of oxidative stress-induced cell death | GO:1903201 | 4.51E-09 | 8.346090638 | TNF,IL10,AKT1,SIRT1,CTNNB1,MMP3,SOD1 |
| GO:BP | cell surface receptor signaling pathway | GO:0007166 | 4.57E-09 | 8.340403517 | NGF,ADIPOQ,NOS3,CXCL12,TNF,IL6,VEGFA,MAPK3,IL10,EDN1,EGF,IL4,AKT1,TP53,SIRT1,CTNNB1,PIK3CA,EP300 |
| GO:BP | nitric oxide biosynthetic process | GO:0006809 | 5.99E-09 | 8.222274125 | NOS3,TNF,IL10,PTGS2,EDN1,AKT1,AGT |
| GO:BP | regulation of protein metabolic process | GO:0051246 | 8.71E-09 | 8.060100687 | NGF,ADIPOQ,TNF,IL6,VEGFA,MAPK3,IL10,PTGS2,EDN1,EGF,IL4,AKT1,TP53,SIRT1,CTNNB1,PIK3CA,EP300,SOD1,AGT |
| GO:BP | positive regulation of epithelial cell proliferation | GO:0050679 | 9.29E-09 | 8.032121676 | CXCL12,TNF,VEGFA,IL10,EGF,AKT1,SIRT1,CTNNB1 |
| GO:BP | regulation of catalytic activity | GO:0050790 | 9.32E-09 | 8.030707187 | NGF,ADIPOQ,NOS3,TNF,VEGFA,MAPK3,PTGS2,EDN1,EGF,IL4,ESR1,AKT1,TP53,SIRT1,CTNNB1,PIK3CA |
| GO:BP | negative regulation of signal transduction | GO:0009968 | 9.39E-09 | 8.027191314 | ADIPOQ,NOS3,CXCL12,TNF,IL6,IL10,PTGS2,IL4,ESR1,AKT1,TP53,SIRT1,CTNNB1 |
| GO:BP | negative regulation of response to stimulus | GO:0048585 | 9.55E-09 | 8.019914063 | ADIPOQ,NOS3,CXCL12,TNF,IL6,IL10,PTGS2,EDN1,IL4,ESR1,AKT1,TP53,SIRT1,CTNNB1 |
| GO:BP | negative regulation of cellular metabolic process | GO:0031324 | 1.01E-08 | 7.994209194 | NGF,ADIPOQ,TNF,IL6,VEGFA,IL10,PTGS2,EDN1,IL4,ESR1,AKT1,TP53,SIRT1,CTNNB1,PIK3CA,EP300,MMP3,ESR2,AGT |
| GO:BP | regulation of defense response | GO:0031347 | 1.05E-08 | 7.978919192 | ADIPOQ,TNF,IL6,MAPK3,IL10,PTGS2,IL4,ESR1,EP300,MMP3,SOD1,AGT |
| GO:BP | response to mechanical stimulus | GO:0009612 | 1.05E-08 | 7.977862062 | CXCL12,TNF,MAPK3,PTGS2,EDN1,CTNNB1,PIK3CA,AGT,MPO |
| GO:BP | immune system process | GO:0002376 | 1.08E-08 | 7.965092737 | ADIPOQ,CXCL12,TNF,IL6,VEGFA,MAPK3,IL10,EDN1,IL4,ESR1,AKT1,TP53,SIRT1,CTNNB1,PIK3CA,GJA1,EP300,CYBB,SOD1,MPO |
| GO:BP | nitric oxide metabolic process | GO:0046209 | 1.10E-08 | 7.959813751 | NOS3,TNF,IL10,PTGS2,EDN1,AKT1,AGT |
| GO:BP | response to corticosteroid | GO:0031960 | 1.12E-08 | 7.952644367 | ADIPOQ,TNF,IL6,IL10,PTGS2,EDN1,PIK3CA,CYBB |
| GO:BP | reactive nitrogen species metabolic process | GO:2001057 | 1.21E-08 | 7.918229758 | NOS3,TNF,IL10,PTGS2,EDN1,AKT1,AGT |
| GO:BP | DNA metabolic process | GO:0006259 | 1.23E-08 | 7.911491166 | ADIPOQ,TNF,IL6,VEGFA,MAPK3,IL10,IL4,AKT1,TP53,SIRT1,CTNNB1,PIK3CA |
| GO:BP | signaling | GO:0023052 | 1.26E-08 | 7.899994526 | NGF,ADIPOQ,NOS3,CXCL12,TNF,IL6,VEGFA,MAPK3,IL10,PTGS2,EDN1,EGF,IL4,ESR1,AKT1,CAT,TP53,SIRT1,CTNNB1,PIK3CA,GJA1,EP300,CYBB,ESR2,SOD1,AGT |
| GO:BP | regulation of cellular response to oxidative stress | GO:1900407 | 1.31E-08 | 7.881078538 | TNF,IL10,AKT1,SIRT1,CTNNB1,MMP3,SOD1 |
| GO:BP | positive regulation of DNA metabolic process | GO:0051054 | 1.39E-08 | 7.856561732 | TNF,IL6,VEGFA,MAPK3,IL4,AKT1,SIRT1,CTNNB1 |
| GO:BP | phosphorylation | GO:0016310 | 1.46E-08 | 7.835785264 | NGF,ADIPOQ,TNF,IL6,VEGFA,MAPK3,PTGS2,EDN1,EGF,IL4,AKT1,TP53,SIRT1,PIK3CA,EP300,SOD1,AGT |
| GO:BP | regulation of small molecule metabolic process | GO:0062012 | 1.50E-08 | 7.823549073 | ADIPOQ,NOS3,TNF,PTGS2,IL4,AKT1,TP53,SIRT1,EP300,SOD1 |
| GO:BP | positive regulation of peptidyl-serine phosphorylation | GO:0033138 | 1.51E-08 | 7.819816291 | NGF,TNF,IL6,VEGFA,PTGS2,AKT1,PIK3CA |
| GO:BP | protein phosphorylation | GO:0006468 | 1.54E-08 | 7.812143266 | NGF,ADIPOQ,TNF,IL6,VEGFA,MAPK3,PTGS2,EDN1,EGF,IL4,AKT1,TP53,SIRT1,PIK3CA,SOD1,AGT |
| GO:BP | sensory perception of pain | GO:0019233 | 1.70E-08 | 7.769003897 | CXCL12,TNF,MAPK3,IL10,PTGS2,EDN1 |
| GO:BP | response to stimulus | GO:0050896 | 1.95E-08 | 7.710116547 | NGF,ADIPOQ,NOS3,CXCL12,ALB,TNF,IL6,VEGFA,MAPK3,IL10,PTGS2,EDN1,EGF,IL4,ESR1,AKT1,CAT,TP53,SIRT1,CTNNB1,PIK3CA,GJA1,EP300,CYBB,MMP3,ESR2,SOD1,AGT,MPO |
| GO:BP | cell growth | GO:0016049 | 2.31E-08 | 7.636320218 | NGF,CXCL12,VEGFA,EDN1,AKT1,TP53,SIRT1,CTNNB1,GJA1,ESR2,AGT |
| GO:BP | positive regulation of epithelial cell migration | GO:0010634 | 2.32E-08 | 7.634479198 | NOS3,VEGFA,PTGS2,EDN1,EGF,AKT1,SIRT1,AGT |
| GO:BP | positive regulation of nucleobase-containing compound metabolic process | GO:0045935 | 2.36E-08 | 7.626680272 | NOS3,TNF,IL6,VEGFA,MAPK3,IL10,EDN1,EGF,IL4,ESR1,AKT1,TP53,SIRT1,CTNNB1,EP300,ESR2,AGT |
| GO:BP | positive regulation of nucleic acid-templated transcription | GO:1903508 | 2.75E-08 | 7.560971239 | TNF,IL6,VEGFA,MAPK3,IL10,EDN1,EGF,IL4,ESR1,AKT1,TP53,SIRT1,CTNNB1,EP300,ESR2,AGT |
| GO:BP | positive regulation of transcription, DNA-templated | GO:0045893 | 2.75E-08 | 7.560971239 | TNF,IL6,VEGFA,MAPK3,IL10,EDN1,EGF,IL4,ESR1,AKT1,TP53,SIRT1,CTNNB1,EP300,ESR2,AGT |
| GO:BP | positive regulation of transcription by RNA polymerase II | GO:0045944 | 2.75E-08 | 7.560333332 | TNF,IL6,VEGFA,MAPK3,IL10,EDN1,IL4,ESR1,AKT1,TP53,SIRT1,CTNNB1,EP300,ESR2 |
| GO:BP | positive regulation of RNA biosynthetic process | GO:1902680 | 2.80E-08 | 7.552772793 | TNF,IL6,VEGFA,MAPK3,IL10,EDN1,EGF,IL4,ESR1,AKT1,TP53,SIRT1,CTNNB1,EP300,ESR2,AGT |
| GO:BP | regulation of inflammatory response | GO:0050727 | 2.80E-08 | 7.55228309 | ADIPOQ,TNF,IL6,IL10,PTGS2,IL4,ESR1,MMP3,SOD1,AGT |
| GO:BP | regulation of response to oxidative stress | GO:1902882 | 2.83E-08 | 7.548083261 | TNF,IL10,AKT1,SIRT1,CTNNB1,MMP3,SOD1 |
| GO:BP | multicellular organismal process | GO:0032501 | 2.88E-08 | 7.540913033 | NGF,ADIPOQ,NOS3,CXCL12,ALB,TNF,IL6,VEGFA,MAPK3,IL10,PTGS2,EDN1,EGF,IL4,ESR1,AKT1,CAT,TP53,SIRT1,CTNNB1,PIK3CA,GJA1,EP300,CYBB |
| GO:BP | regulation of growth | GO:0040008 | 2.89E-08 | 7.539405947 | NGF,CXCL12,VEGFA,EDN1,AKT1,TP53,SIRT1,PIK3CA,GJA1,ESR2,SOD1,AGT |
| GO:BP | regulation of endothelial cell proliferation | GO:0001936 | 2.92E-08 | 7.535311427 | CXCL12,TNF,VEGFA,IL10,EGF,AKT1,SIRT1 |
| GO:BP | developmental process | GO:0032502 | 2.98E-08 | 7.526245055 | NGF,ADIPOQ,NOS3,CXCL12,TNF,IL6,VEGFA,MAPK3,IL10,PTGS2,EDN1,EGF,IL4,ESR1,AKT1,CAT,TP53,SIRT1,CTNNB1,PIK3CA,GJA1,EP300,CYBB |
| GO:BP | negative regulation of apoptotic signaling pathway | GO:2001234 | 3.05E-08 | 7.515109887 | NOS3,CXCL12,TNF,PTGS2,IL4,AKT1,SIRT1,CTNNB1 |
| GO:BP | cell death in response to oxidative stress | GO:0036473 | 3.07E-08 | 7.513281388 | TNF,IL10,AKT1,SIRT1,CTNNB1,MMP3,SOD1 |
| GO:BP | blood circulation | GO:0008015 | 3.21E-08 | 7.493930983 | ADIPOQ,NOS3,CXCL12,TNF,VEGFA,PTGS2,EDN1,PIK3CA,GJA1,SOD1,AGT |
| GO:BP | negative regulation of metabolic process | GO:0009892 | 3.70E-08 | 7.432021366 | NGF,ADIPOQ,TNF,IL6,VEGFA,IL10,PTGS2,EDN1,IL4,ESR1,AKT1,TP53,SIRT1,CTNNB1,PIK3CA,GJA1,EP300,MMP3,ESR2,SOD1,AGT |
| GO:BP | positive regulation of leukocyte chemotaxis | GO:0002690 | 3.77E-08 | 7.423457202 | CXCL12,IL6,VEGFA,MAPK3,EDN1,IL4 |
| GO:BP | positive regulation of small molecule metabolic process | GO:0062013 | 3.79E-08 | 7.421641999 | ADIPOQ,NOS3,TNF,PTGS2,IL4,AKT1,SIRT1 |
| GO:BP | negative regulation of gene expression | GO:0010629 | 5.10E-08 | 7.292115799 | ADIPOQ,TNF,IL6,VEGFA,IL10,EDN1,IL4,ESR1,AKT1,TP53,SIRT1,CTNNB1,PIK3CA,GJA1 |
| GO:BP | cellular response to lipid | GO:0071396 | 5.53E-08 | 7.257632827 | NOS3,TNF,IL6,MAPK3,IL10,EDN1,ESR1,AKT1,SIRT1,EP300,ESR2 |
| GO:BP | regulation of lipid localization | GO:1905952 | 5.60E-08 | 7.251681379 | ADIPOQ,TNF,IL6,EDN1,EGF,AKT1,SIRT1,AGT |
| GO:BP | leukocyte differentiation | GO:0002521 | 6.60E-08 | 7.180567221 | ADIPOQ,TNF,IL6,VEGFA,IL10,IL4,TP53,SIRT1,CTNNB1,EP300,SOD1 |
| GO:BP | response to lipopolysaccharide | GO:0032496 | 6.63E-08 | 7.178261538 | NOS3,TNF,IL6,MAPK3,IL10,PTGS2,EDN1,AKT1 |
| GO:BP | endothelial cell proliferation | GO:0001935 | 6.83E-08 | 7.165320701 | CXCL12,TNF,VEGFA,IL10,EGF,AKT1,SIRT1 |
| GO:BP | positive regulation of immune system process | GO:0002684 | 7.08E-08 | 7.149691846 | CXCL12,TNF,IL6,VEGFA,MAPK3,IL10,EDN1,IL4,AKT1,SIRT1,PIK3CA,EP300 |
| GO:BP | response to tumor necrosis factor | GO:0034612 | 7.27E-08 | 7.138210166 | ADIPOQ,TNF,MAPK3,PTGS2,EDN1,AKT1,TP53,SIRT1 |
| GO:BP | positive regulation of protein kinase activity | GO:0045860 | 7.66E-08 | 7.115815166 | ADIPOQ,TNF,VEGFA,EDN1,EGF,IL4,AKT1,SIRT1,PIK3CA,AGT |
| GO:BP | positive regulation of RNA metabolic process | GO:0051254 | 8.54E-08 | 7.068565355 | TNF,IL6,VEGFA,MAPK3,IL10,EDN1,EGF,IL4,ESR1,AKT1,TP53,SIRT1,CTNNB1,EP300,ESR2,AGT |
| GO:BP | response to heat | GO:0009408 | 9.23E-08 | 7.034935311 | NOS3,CXCL12,PTGS2,AKT1,SIRT1,EP300,SOD1 |
| GO:BP | response to glucocorticoid | GO:0051384 | 9.57E-08 | 7.019001676 | ADIPOQ,TNF,IL6,IL10,PTGS2,EDN1,PIK3CA |
| GO:BP | response to molecule of bacterial origin | GO:0002237 | 1.04E-07 | 6.983383594 | NOS3,TNF,IL6,MAPK3,IL10,PTGS2,EDN1,AKT1 |
| GO:BP | regulation of angiogenesis | GO:0045765 | 1.11E-07 | 6.955588258 | NOS3,TNF,IL6,VEGFA,IL10,SIRT1,CTNNB1,CYBB,AGT |
| GO:BP | regulation of peptidyl-serine phosphorylation | GO:0033135 | 1.11E-07 | 6.953898463 | NGF,TNF,IL6,VEGFA,PTGS2,AKT1,PIK3CA |
| GO:BP | leukocyte chemotaxis | GO:0030595 | 1.14E-07 | 6.942612918 | CXCL12,IL6,VEGFA,MAPK3,IL10,EDN1,IL4 |
| GO:BP | regulation of vasculature development | GO:1901342 | 1.26E-07 | 6.900567045 | NOS3,TNF,IL6,VEGFA,IL10,SIRT1,CTNNB1,CYBB,AGT |
| GO:BP | response to other organism | GO:0051707 | 1.33E-07 | 6.877098826 | ADIPOQ,NOS3,CXCL12,TNF,IL6,MAPK3,IL10,PTGS2,EDN1,IL4,AKT1,TP53,EP300,CYBB |
| GO:BP | morphogenesis of a branching epithelium | GO:0061138 | 1.35E-07 | 6.869913616 | TNF,VEGFA,IL10,EDN1,EGF,ESR1,CTNNB1,AGT |
| GO:BP | response to external biotic stimulus | GO:0043207 | 1.36E-07 | 6.86541652 | ADIPOQ,NOS3,CXCL12,TNF,IL6,MAPK3,IL10,PTGS2,EDN1,IL4,AKT1,TP53,EP300,CYBB |
| GO:BP | inflammatory response | GO:0006954 | 1.37E-07 | 6.863881622 | ADIPOQ,TNF,IL6,IL10,PTGS2,IL4,ESR1,AKT1,CYBB,MMP3,SOD1,AGT |
| GO:BP | positive regulation of cell death | GO:0010942 | 1.41E-07 | 6.851399827 | ADIPOQ,TNF,IL6,IL10,PTGS2,TP53,SIRT1,CTNNB1,MMP3,SOD1,AGT |
| GO:BP | regulation of cell growth | GO:0001558 | 1.47E-07 | 6.831806942 | NGF,CXCL12,VEGFA,EDN1,AKT1,TP53,SIRT1,GJA1,ESR2,AGT |
| GO:BP | positive regulation of response to external stimulus | GO:0032103 | 1.51E-07 | 6.820263108 | CXCL12,TNF,IL6,VEGFA,MAPK3,PTGS2,EDN1,IL4 |
| GO:BP | regulation of nitric oxide biosynthetic process | GO:0045428 | 1.55E-07 | 6.808279463 | TNF,IL10,PTGS2,EDN1,AKT1,AGT |
| GO:BP | regulation of vascular associated smooth muscle cell proliferation | GO:1904705 | 1.55E-07 | 6.808279463 | ADIPOQ,TNF,IL10,EDN1,GJA1,AGT |
| GO:BP | reproductive structure development | GO:0048608 | 1.56E-07 | 6.807636627 | NOS3,VEGFA,IL10,PTGS2,ESR1,AKT1,SIRT1,CTNNB1,GJA1,SOD1 |
| GO:BP | reproductive system development | GO:0061458 | 1.66E-07 | 6.778726481 | NOS3,VEGFA,IL10,PTGS2,ESR1,AKT1,SIRT1,CTNNB1,GJA1,SOD1 |
| GO:BP | positive regulation of peptidyl-tyrosine phosphorylation | GO:0050731 | 1.67E-07 | 6.776290839 | ADIPOQ,TNF,IL6,VEGFA,EGF,IL4,TP53,AGT |
| GO:BP | circulatory system process | GO:0003013 | 1.72E-07 | 6.763386852 | ADIPOQ,NOS3,CXCL12,TNF,VEGFA,PTGS2,EDN1,PIK3CA,GJA1,SOD1,AGT |
| GO:BP | vascular associated smooth muscle cell proliferation | GO:1990874 | 1.74E-07 | 6.759451604 | ADIPOQ,TNF,IL10,EDN1,GJA1,AGT |
| GO:BP | regulation of neuron death | GO:1901214 | 1.78E-07 | 6.749400157 | NGF,TNF,IL10,AKT1,TP53,SIRT1,CTNNB1,PIK3CA,SOD1 |
| GO:BP | regulation of leukocyte chemotaxis | GO:0002688 | 1.83E-07 | 6.738184002 | CXCL12,IL6,VEGFA,MAPK3,EDN1,IL4 |
| GO:BP | response to biotic stimulus | GO:0009607 | 1.91E-07 | 6.719564032 | ADIPOQ,NOS3,CXCL12,TNF,IL6,MAPK3,IL10,PTGS2,EDN1,IL4,AKT1,TP53,EP300,CYBB |
| GO:BP | negative regulation of multicellular organismal process | GO:0051241 | 1.93E-07 | 6.715507083 | ADIPOQ,NOS3,TNF,IL6,IL10,PTGS2,EDN1,IL4,TP53,CTNNB1,GJA1,SOD1,AGT |
| GO:BP | positive regulation of macromolecule biosynthetic process | GO:0010557 | 2.16E-07 | 6.665952709 | TNF,IL6,VEGFA,MAPK3,IL10,EDN1,EGF,IL4,ESR1,AKT1,TP53,SIRT1,CTNNB1,EP300,ESR2,AGT |
| GO:BP | embryonic organ development | GO:0048568 | 2.16E-07 | 6.66510193 | TNF,VEGFA,MAPK3,IL10,EDN1,AKT1,TP53,CTNNB1,GJA1,SOD1 |
| GO:BP | regulation of nitric oxide metabolic process | GO:0080164 | 2.17E-07 | 6.664502626 | TNF,IL10,PTGS2,EDN1,AKT1,AGT |
| GO:BP | regulation of blood vessel endothelial cell migration | GO:0043535 | 2.24E-07 | 6.648854323 | NOS3,TNF,VEGFA,PTGS2,AKT1,SIRT1 |
| GO:BP | morphogenesis of a branching structure | GO:0001763 | 2.43E-07 | 6.614116309 | TNF,VEGFA,IL10,EDN1,EGF,ESR1,CTNNB1,AGT |
| GO:BP | cell differentiation | GO:0030154 | 2.47E-07 | 6.606730713 | NGF,ADIPOQ,CXCL12,TNF,IL6,VEGFA,MAPK3,IL10,PTGS2,EDN1,IL4,ESR1,AKT1,CAT,TP53,SIRT1,CTNNB1,GJA1,EP300 |
| GO:BP | regulation of metabolic process | GO:0019222 | 2.49E-07 | 6.603881862 | NGF,ADIPOQ,NOS3,TNF,IL6,VEGFA,MAPK3,IL10,PTGS2,EDN1,EGF,IL4,ESR1,AKT1,CAT,TP53,SIRT1,CTNNB1,PIK3CA,GJA1,EP300,CYBB,MMP3,ESR2,SOD1,AGT |
| GO:BP | cytokine-mediated signaling pathway | GO:0019221 | 2.66E-07 | 6.574391878 | ADIPOQ,CXCL12,TNF,IL6,MAPK3,EDN1,AKT1,TP53,SIRT1 |
| GO:BP | regulation of cell-cell adhesion | GO:0022407 | 2.92E-07 | 6.534997993 | ADIPOQ,CXCL12,TNF,IL6,VEGFA,IL10,IL4,AKT1,PIK3CA |
| GO:BP | positive regulation of endothelial cell proliferation | GO:0001938 | 2.94E-07 | 6.531938945 | CXCL12,VEGFA,IL10,EGF,AKT1,SIRT1 |
| GO:BP | cell activation | GO:0001775 | 3.01E-07 | 6.521160268 | NOS3,TNF,IL6,IL10,EDN1,IL4,AKT1,TP53,CTNNB1,PIK3CA,GJA1,EP300,SOD1 |
| GO:BP | embryo development | GO:0009790 | 3.23E-07 | 6.491451445 | ADIPOQ,NOS3,TNF,VEGFA,MAPK3,IL10,EDN1,AKT1,TP53,CTNNB1,GJA1,EP300,SOD1 |
| GO:BP | negative regulation of cellular protein metabolic process | GO:0032269 | 3.37E-07 | 6.472380444 | NGF,ADIPOQ,TNF,VEGFA,IL10,PTGS2,EDN1,AKT1,TP53,SIRT1,CTNNB1 |
| GO:BP | cellular developmental process | GO:0048869 | 3.53E-07 | 6.451985785 | NGF,ADIPOQ,CXCL12,TNF,IL6,VEGFA,MAPK3,IL10,PTGS2,EDN1,IL4,ESR1,AKT1,CAT,TP53,SIRT1,CTNNB1,GJA1,EP300 |
| GO:BP | cellular response to environmental stimulus | GO:0104004 | 3.54E-07 | 6.451099982 | MAPK3,PTGS2,TP53,SIRT1,PIK3CA,GJA1,EP300,MMP3,AGT |
| GO:BP | cellular response to abiotic stimulus | GO:0071214 | 3.54E-07 | 6.451099982 | MAPK3,PTGS2,TP53,SIRT1,PIK3CA,GJA1,EP300,MMP3,AGT |
| GO:BP | cellular response to toxic substance | GO:0097237 | 3.68E-07 | 6.43394858 | NOS3,ALB,TNF,PTGS2,CAT,SOD1,MPO |
| GO:BP | aging | GO:0007568 | 3.71E-07 | 6.430762949 | IL10,PTGS2,EDN1,CAT,TP53,SIRT1,SOD1,AGT,MPO |
| GO:BP | positive regulation of cell development | GO:0010720 | 3.76E-07 | 6.42473231 | NGF,ADIPOQ,CXCL12,TNF,IL6,VEGFA |
| GO:BP | regulation of protein kinase activity | GO:0045859 | 4.13E-07 | 6.384167793 | ADIPOQ,TNF,VEGFA,EDN1,EGF,IL4,AKT1,SIRT1,PIK3CA,SOD1,AGT |
| GO:BP | positive regulation of cytokine production | GO:0001819 | 4.16E-07 | 6.380399235 | ADIPOQ,TNF,IL6,IL10,PTGS2,IL4,SIRT1,CYBB,SOD1,AGT |
| GO:BP | positive regulation of chemotaxis | GO:0050921 | 4.37E-07 | 6.359298319 | CXCL12,IL6,VEGFA,MAPK3,EDN1,IL4 |
| GO:BP | animal organ morphogenesis | GO:0009887 | 4.46E-07 | 6.350809833 | NOS3,TNF,IL6,VEGFA,MAPK3,EDN1,ESR1,TP53,CTNNB1,GJA1,EP300,SOD1,AGT |
| GO:BP | multi-multicellular organism process | GO:0044706 | 4.53E-07 | 6.343972392 | VEGFA,PTGS2,EDN1,ESR1,AKT1,GJA1,SOD1,AGT |
| GO:BP | DNA biosynthetic process | GO:0071897 | 4.57E-07 | 6.33972042 | ADIPOQ,TNF,VEGFA,MAPK3,TP53,SIRT1,CTNNB1 |
| GO:BP | biological process involved in interspecies interaction between organisms | GO:0044419 | 4.62E-07 | 6.335769626 | ADIPOQ,NOS3,CXCL12,TNF,IL6,MAPK3,IL10,PTGS2,EDN1,IL4,AKT1,TP53,EP300,CYBB |
| GO:BP | positive regulation of kinase activity | GO:0033674 | 4.72E-07 | 6.326241159 | ADIPOQ,TNF,VEGFA,EDN1,EGF,IL4,AKT1,SIRT1,PIK3CA,AGT |
| GO:BP | response to estradiol | GO:0032355 | 5.27E-07 | 6.278319613 | IL10,PTGS2,ESR1,CAT,CTNNB1,ESR2,AGT |
| GO:BP | neuron death | GO:0070997 | 5.27E-07 | 6.278250552 | NGF,TNF,IL10,AKT1,TP53,SIRT1,CTNNB1,PIK3CA,SOD1 |
| GO:BP | regulation of cell development | GO:0060284 | 5.28E-07 | 6.277415598 | NGF,ADIPOQ,CXCL12,TNF,IL6,VEGFA,AKT1,TP53,CTNNB1 |
| GO:BP | I-kappaB kinase/NF-kappaB signaling | GO:0007249 | 5.28E-07 | 6.277117203 | ADIPOQ,TNF,EDN1,ESR1,AKT1,SIRT1,CTNNB1,GJA1 |
| GO:BP | superoxide metabolic process | GO:0006801 | 5.52E-07 | 6.257826277 | NOS3,EDN1,CYBB,SOD1,AGT,MPO |
| GO:BP | regulation of localization | GO:0032879 | 5.57E-07 | 6.253978613 | ADIPOQ,NOS3,CXCL12,TNF,IL6,VEGFA,MAPK3,PTGS2,EDN1,EGF,IL4,AKT1,SIRT1,CTNNB1,GJA1,CYBB,SOD1,AGT |
| GO:BP | response to UV | GO:0009411 | 5.72E-07 | 6.242317043 | PTGS2,AKT1,CAT,TP53,SIRT1,EP300,MMP3 |
| GO:BP | peptidyl-tyrosine phosphorylation | GO:0018108 | 5.96E-07 | 6.22498782 | ADIPOQ,TNF,IL6,VEGFA,MAPK3,EGF,IL4,TP53 |
| GO:BP | positive regulation of protein serine/threonine kinase activity | GO:0071902 | 6.15E-07 | 6.21095354 | ADIPOQ,TNF,VEGFA,EDN1,EGF,AKT1,SIRT1 |
| GO:BP | regulation of cellular metabolic process | GO:0031323 | 6.21E-07 | 6.206713388 | NGF,ADIPOQ,NOS3,TNF,IL6,VEGFA,MAPK3,IL10,PTGS2,EDN1,EGF,IL4,ESR1,AKT1,CAT,TP53,SIRT1,CTNNB1,PIK3CA,EP300,MMP3,ESR2,SOD1,AGT |
| GO:BP | negative regulation of nitrogen compound metabolic process | GO:0051172 | 6.23E-07 | 6.205253975 | NGF,ADIPOQ,TNF,IL6,VEGFA,IL10,PTGS2,EDN1,IL4,ESR1,AKT1,TP53,SIRT1,CTNNB1,EP300,ESR2,AGT |
| GO:BP | peptidyl-tyrosine modification | GO:0018212 | 6.35E-07 | 6.19737481 | ADIPOQ,TNF,IL6,VEGFA,MAPK3,EGF,IL4,TP53 |
| GO:BP | regulation of anatomical structure size | GO:0090066 | 6.66E-07 | 6.17663829 | NGF,NOS3,CXCL12,TNF,VEGFA,PTGS2,EDN1,PIK3CA,SOD1,AGT |
| GO:BP | cellular catabolic process | GO:0044248 | 6.73E-07 | 6.171781451 | ADIPOQ,NOS3,TNF,IL6,MAPK3,IL10,EGF,IL4,AKT1,CAT,TP53,SIRT1,CTNNB1,PIK3CA,EP300 |
| GO:BP | negative regulation of protein metabolic process | GO:0051248 | 6.79E-07 | 6.168398934 | NGF,ADIPOQ,TNF,VEGFA,IL10,PTGS2,EDN1,AKT1,TP53,SIRT1,CTNNB1 |
| GO:BP | regulation of cellular catabolic process | GO:0031329 | 6.83E-07 | 6.165693312 | TNF,IL6,MAPK3,IL10,EGF,IL4,AKT1,TP53,SIRT1,PIK3CA,EP300 |
| GO:BP | phosphate-containing compound metabolic process | GO:0006796 | 7.16E-07 | 6.144944815 | NGF,ADIPOQ,NOS3,TNF,IL6,VEGFA,MAPK3,PTGS2,EDN1,EGF,IL4,AKT1,TP53,SIRT1,PIK3CA,EP300,SOD1,AGT |
| GO:BP | regulation of biosynthetic process | GO:0009889 | 7.22E-07 | 6.141706231 | ADIPOQ,NOS3,TNF,IL6,VEGFA,MAPK3,IL10,PTGS2,EDN1,EGF,IL4,ESR1,AKT1,CAT,TP53,SIRT1,CTNNB1,EP300,ESR2,SOD1,AGT |
| GO:BP | regulation of macromolecule metabolic process | GO:0060255 | 7.51E-07 | 6.124116789 | NGF,ADIPOQ,NOS3,TNF,IL6,VEGFA,MAPK3,IL10,PTGS2,EDN1,EGF,IL4,ESR1,AKT1,CAT,TP53,SIRT1,CTNNB1,PIK3CA,GJA1,EP300,CYBB,ESR2,SOD1,AGT |
| GO:BP | regulation of neuroinflammatory response | GO:0150077 | 7.53E-07 | 6.123099737 | TNF,IL6,PTGS2,IL4,MMP3 |
| GO:BP | regulation of epithelial cell proliferation | GO:0050678 | 7.80E-07 | 6.107984538 | CXCL12,TNF,VEGFA,IL10,EGF,AKT1,SIRT1,CTNNB1 |
| GO:BP | cell chemotaxis | GO:0060326 | 7.93E-07 | 6.100598877 | CXCL12,IL6,VEGFA,MAPK3,IL10,EDN1,IL4 |
| GO:BP | phosphorus metabolic process | GO:0006793 | 8.13E-07 | 6.089789844 | NGF,ADIPOQ,NOS3,TNF,IL6,VEGFA,MAPK3,PTGS2,EDN1,EGF,IL4,AKT1,TP53,SIRT1,PIK3CA,EP300,SOD1,AGT |
| GO:BP | cellular response to peptide | GO:1901653 | 8.26E-07 | 6.083197964 | ADIPOQ,TNF,EDN1,AKT1,TP53,SIRT1,PIK3CA,GJA1,AGT |
| GO:BP | regulation of cellular biosynthetic process | GO:0031326 | 8.50E-07 | 6.07046542 | ADIPOQ,NOS3,TNF,IL6,VEGFA,MAPK3,IL10,PTGS2,EDN1,EGF,IL4,ESR1,AKT1,CAT,TP53,SIRT1,CTNNB1 |
| GO:BP | cellular response to decreased oxygen levels | GO:0036294 | 8.53E-07 | 6.069112011 | VEGFA,PTGS2,EDN1,AKT1,TP53,SIRT1,CYBB |
| GO:BP | positive regulation of apoptotic process | GO:0043065 | 8.59E-07 | 6.065883469 | ADIPOQ,TNF,IL6,IL10,PTGS2,TP53,SIRT1,CTNNB1,SOD1,AGT |
| GO:BP | hematopoietic or lymphoid organ development | GO:0048534 | 8.66E-07 | 6.062689028 | ADIPOQ,TNF,IL6,VEGFA,MAPK3,IL10,IL4,TP53,SIRT1,CTNNB1,EP300,SOD1 |
| GO:BP | regulation of ion transport | GO:0043269 | 8.94E-07 | 6.04869694 | NOS3,CXCL12,TNF,PTGS2,EDN1,EGF,AKT1,CTNNB1,GJA1,CYBB,AGT |
| GO:BP | leukocyte apoptotic process | GO:0071887 | 9.13E-07 | 6.039586192 | CXCL12,IL6,IL10,AKT1,TP53,SIRT1 |
| GO:BP | positive regulation of transport | GO:0051050 | 9.14E-07 | 6.038832903 | ADIPOQ,CXCL12,TNF,VEGFA,PTGS2,EDN1,EGF,IL4,AKT1,SIRT1,SOD1,AGT |
| GO:BP | blood vessel endothelial cell migration | GO:0043534 | 9.64E-07 | 6.016122523 | NOS3,TNF,VEGFA,PTGS2,AKT1,SIRT1 |
| GO:BP | leukocyte activation | GO:0045321 | 1.08E-06 | 5.967436234 | TNF,IL6,IL10,EDN1,IL4,AKT1,TP53,CTNNB1,PIK3CA,GJA1,EP300,SOD1 |
| GO:BP | positive regulation of programmed cell death | GO:0043068 | 1.08E-06 | 5.966297463 | ADIPOQ,TNF,IL6,IL10,PTGS2,TP53,SIRT1,CTNNB1,SOD1,AGT |
| GO:BP | positive regulation of vascular associated smooth muscle cell proliferation | GO:1904707 | 1.19E-06 | 5.926157799 | TNF,IL10,EDN1,GJA1,AGT |
| GO:BP | cellular response to biotic stimulus | GO:0071216 | 1.32E-06 | 5.878047306 | NOS3,TNF,IL6,MAPK3,IL10,AKT1,TP53 |
| GO:BP | cell development | GO:0048468 | 1.34E-06 | 5.871422298 | NGF,ADIPOQ,CXCL12,TNF,IL6,VEGFA,MAPK3,EDN1,ESR1,AKT1,TP53,CTNNB1,GJA1,EP300,SOD1,AGT |
| GO:BP | response to bacterium | GO:0009617 | 1.35E-06 | 5.868447569 | ADIPOQ,NOS3,TNF,IL6,MAPK3,IL10,PTGS2,EDN1,AKT1 |
| GO:BP | neuroinflammatory response | GO:0150076 | 1.35E-06 | 5.86826918 | TNF,IL6,PTGS2,IL4,MMP3 |
| GO:BP | regulation of DNA biosynthetic process | GO:2000278 | 1.37E-06 | 5.863118105 | ADIPOQ,TNF,VEGFA,MAPK3,TP53,CTNNB1 |
| GO:BP | positive regulation of phosphatidylinositol 3-kinase signaling | GO:0014068 | 1.46E-06 | 5.835902664 | TNF,VEGFA,EGF,CAT,SIRT1,AGT |
| GO:BP | chemical homeostasis | GO:0048878 | 1.51E-06 | 5.822010011 | ADIPOQ,CXCL12,IL6,VEGFA,MAPK3,EDN1,ESR1,AKT1,SIRT1,PIK3CA,GJA1,SOD1,AGT |
| GO:BP | positive regulation of blood vessel endothelial cell migration | GO:0043536 | 1.53E-06 | 5.813925326 | NOS3,VEGFA,PTGS2,AKT1,SIRT1 |
| GO:BP | branching morphogenesis of an epithelial tube | GO:0048754 | 1.59E-06 | 5.797362381 | TNF,VEGFA,EDN1,EGF,ESR1,CTNNB1,AGT |
| GO:BP | cellular response to oxygen levels | GO:0071453 | 1.70E-06 | 5.769163209 | VEGFA,PTGS2,EDN1,AKT1,TP53,SIRT1,CYBB |
| GO:BP | immune system development | GO:0002520 | 1.73E-06 | 5.762496066 | ADIPOQ,TNF,IL6,VEGFA,MAPK3,IL10,IL4,TP53,SIRT1,CTNNB1,EP300,SOD1 |
| GO:BP | regulation of peptidyl-tyrosine phosphorylation | GO:0050730 | 1.80E-06 | 5.744560285 | ADIPOQ,TNF,IL6,VEGFA,EGF,IL4,TP53 |
| GO:BP | regulation of nitrogen compound metabolic process | GO:0051171 | 1.81E-06 | 5.741700854 | NGF,ADIPOQ,NOS3,TNF,IL6,VEGFA,MAPK3,IL10,PTGS2,EDN1,EGF,IL4,ESR1,AKT1,CAT,TP53,SIRT1,CTNNB1,PIK3CA,EP300,ESR2,SOD1,AGT |
| GO:BP | regulation of nucleobase-containing compound metabolic process | GO:0019219 | 1.82E-06 | 5.739457678 | ADIPOQ,NOS3,TNF,IL6,VEGFA,MAPK3,IL10,EDN1,EGF,IL4,ESR1,AKT1,CAT,TP53,SIRT1,CTNNB1,PIK3CA,EP300 |
| GO:BP | negative regulation of macromolecule metabolic process | GO:0010605 | 1.83E-06 | 5.73720631 | NGF,ADIPOQ,TNF,IL6,VEGFA,IL10,PTGS2,EDN1,IL4,ESR1,AKT1,TP53,SIRT1,CTNNB1,PIK3CA,GJA1,EP300 |
| GO:BP | positive regulation of morphogenesis of an epithelium | GO:1905332 | 1.88E-06 | 5.72637811 | VEGFA,EGF,CTNNB1,GJA1,AGT |
| GO:BP | response to radiation | GO:0009314 | 1.88E-06 | 5.725194247 | CXCL12,TNF,PTGS2,AKT1,CAT,TP53,SIRT1,EP300,MMP3 |
| GO:BP | enzyme linked receptor protein signaling pathway | GO:0007167 | 1.90E-06 | 5.721721201 | NGF,ADIPOQ,VEGFA,MAPK3,EGF,AKT1,TP53,SIRT1,CTNNB1,PIK3CA,EP300,AGT |
| GO:BP | cellular response to tumor necrosis factor | GO:0071356 | 2.02E-06 | 5.695197543 | ADIPOQ,TNF,MAPK3,EDN1,AKT1,TP53,SIRT1 |
| GO:BP | glucose metabolic process | GO:0006006 | 2.12E-06 | 5.674147468 | ADIPOQ,TNF,AKT1,TP53,SIRT1,PIK3CA,EP300 |
| GO:BP | tissue homeostasis | GO:0001894 | 2.40E-06 | 5.619832087 | NOS3,ALB,IL6,VEGFA,PTGS2,CTNNB1,GJA1,SOD1 |
| GO:BP | regulation of autophagy | GO:0010506 | 2.47E-06 | 5.607584591 | MAPK3,IL10,IL4,AKT1,TP53,SIRT1,PIK3CA,EP300 |
| GO:BP | regulation of kinase activity | GO:0043549 | 2.50E-06 | 5.601812613 | ADIPOQ,TNF,VEGFA,EDN1,EGF,IL4,AKT1,SIRT1,PIK3CA,SOD1,AGT |
| GO:BP | catabolic process | GO:0009056 | 2.55E-06 | 5.594050427 | ADIPOQ,NOS3,TNF,IL6,MAPK3,IL10,EGF,IL4,AKT1,CAT,TP53,SIRT1,CTNNB1,PIK3CA,EP300,MMP3 |
| GO:BP | regulation of fat cell differentiation | GO:0045598 | 2.59E-06 | 5.587166429 | ADIPOQ,TNF,IL6,PTGS2,AKT1,SIRT1 |
| GO:BP | positive regulation of nitric oxide biosynthetic process | GO:0045429 | 2.86E-06 | 5.543659896 | TNF,PTGS2,EDN1,AKT1,AGT |
| GO:BP | response to cytokine | GO:0034097 | 2.88E-06 | 5.540941061 | ADIPOQ,CXCL12,TNF,IL6,MAPK3,PTGS2,EDN1,AKT1,TP53,SIRT1 |
| GO:BP | biosynthetic process | GO:0009058 | 3.00E-06 | 5.522502233 | ADIPOQ,NOS3,TNF,IL6,VEGFA,MAPK3,IL10,PTGS2,EDN1,EGF,IL4,ESR1,AKT1,CAT,TP53,SIRT1,CTNNB1,PIK3CA,EP300,CYBB,ESR2,SOD1,AGT,MPO |
| GO:BP | negative regulation of biosynthetic process | GO:0009890 | 3.11E-06 | 5.506817739 | ADIPOQ,TNF,IL6,VEGFA,IL10,EDN1,IL4,ESR1,TP53,SIRT1,CTNNB1,EP300,ESR2,SOD1 |
| GO:BP | negative regulation of small molecule metabolic process | GO:0062014 | 3.33E-06 | 5.478181133 | ADIPOQ,AKT1,TP53,SIRT1,EP300,SOD1 |
| GO:BP | regulation of primary metabolic process | GO:0080090 | 3.36E-06 | 5.473551453 | NGF,ADIPOQ,NOS3,TNF,IL6,VEGFA,MAPK3,IL10,PTGS2,EDN1,EGF,IL4,ESR1,AKT1,CAT,TP53,SIRT1,CTNNB1,PIK3CA |
| GO:BP | regulation of catabolic process | GO:0009894 | 3.38E-06 | 5.470955952 | TNF,IL6,MAPK3,IL10,EGF,IL4,AKT1,TP53,SIRT1,PIK3CA,EP300 |
| GO:BP | positive regulation of nitric oxide metabolic process | GO:1904407 | 3.72E-06 | 5.429979208 | TNF,PTGS2,EDN1,AKT1,AGT |
| GO:BP | generation of precursor metabolites and energy | GO:0006091 | 3.78E-06 | 5.422929561 | ADIPOQ,TNF,IL4,AKT1,CAT,TP53,PIK3CA,EP300,CYBB |
| GO:BP | response to temperature stimulus | GO:0009266 | 3.80E-06 | 5.419989572 | NOS3,CXCL12,PTGS2,AKT1,SIRT1,EP300,SOD1 |
| GO:BP | regulation of production of miRNAs involved in gene silencing by miRNA | GO:1903798 | 3.98E-06 | 5.400276285 | TNF,IL6,ESR1,TP53 |
| GO:BP | regulation of production of small RNA involved in gene silencing by RNA | GO:0070920 | 3.98E-06 | 5.400276285 | TNF,IL6,ESR1,TP53 |
| GO:BP | system process | GO:0003008 | 4.13E-06 | 5.384145719 | NGF,ADIPOQ,NOS3,CXCL12,TNF,VEGFA,MAPK3,IL10,PTGS2,EDN1,AKT1,PIK3CA,GJA1,EP300,SOD1,AGT |
| GO:BP | intrinsic apoptotic signaling pathway | GO:0097193 | 4.41E-06 | 5.355894256 | CXCL12,TNF,PTGS2,AKT1,TP53,SIRT1,EP300,SOD1 |
| GO:BP | negative regulation of catalytic activity | GO:0043086 | 4.64E-06 | 5.333035833 | NGF,ADIPOQ,NOS3,TNF,VEGFA,PTGS2,AKT1,TP53,SIRT1 |
| GO:BP | cellular biosynthetic process | GO:0044249 | 4.92E-06 | 5.308386458 | ADIPOQ,NOS3,TNF,IL6,VEGFA,MAPK3,IL10,PTGS2,EDN1,EGF,IL4,ESR1,AKT1,CAT,TP53,SIRT1,CTNNB1,PIK3CA,EP300,CYBB,ESR2,SOD1,AGT |
| GO:BP | positive regulation of receptor signaling pathway via JAK-STAT | GO:0046427 | 5.37E-06 | 5.270047678 | TNF,IL6,IL10,IL4,AGT |
| GO:BP | regulation of receptor signaling pathway via JAK-STAT | GO:0046425 | 5.43E-06 | 5.265036844 | TNF,IL6,IL10,EGF,IL4,AGT |
| GO:BP | regulation of binding | GO:0051098 | 5.56E-06 | 5.255262082 | NGF,ADIPOQ,MAPK3,IL10,EGF,AKT1,CTNNB1,EP300 |
| GO:BP | cellular oxidant detoxification | GO:0098869 | 5.65E-06 | 5.247969986 | NOS3,ALB,PTGS2,CAT,SOD1,MPO |
| GO:BP | regulation of cellular macromolecule biosynthetic process | GO:2000112 | 5.84E-06 | 5.233446924 | ADIPOQ,TNF,IL6,VEGFA,MAPK3,EGF,AKT1,TP53,CTNNB1 |
| GO:BP | regulation of nervous system development | GO:0051960 | 6.00E-06 | 5.221552855 | NGF,CXCL12,TNF,IL6,VEGFA,AKT1,TP53,CTNNB1 |
| GO:BP | female pregnancy | GO:0007565 | 6.10E-06 | 5.214404536 | VEGFA,PTGS2,ESR1,AKT1,GJA1,SOD1,AGT |
| GO:BP | regulation of blood pressure | GO:0008217 | 6.10E-06 | 5.214404536 | ADIPOQ,NOS3,TNF,PTGS2,EDN1,SOD1,AGT |
| GO:BP | regulation of chemotaxis | GO:0050920 | 6.12E-06 | 5.212991382 | CXCL12,IL6,VEGFA,MAPK3,EDN1,IL4 |
| GO:BP | negative regulation of cell-cell adhesion | GO:0022408 | 6.26E-06 | 5.203642803 | ADIPOQ,CXCL12,VEGFA,IL10,IL4,AKT1 |
| GO:BP | muscle structure development | GO:0061061 | 6.30E-06 | 5.200784182 | TNF,VEGFA,EDN1,IL4,AKT1,SIRT1,CTNNB1,GJA1,EP300,AGT |
| GO:BP | response to fatty acid | GO:0070542 | 6.62E-06 | 5.17909304 | ADIPOQ,PTGS2,EDN1,CAT,PIK3CA |
| GO:BP | fat cell differentiation | GO:0045444 | 6.64E-06 | 5.177650877 | ADIPOQ,TNF,IL6,PTGS2,AKT1,SIRT1,EP300 |
| GO:BP | cell-cell adhesion | GO:0098609 | 7.07E-06 | 5.150649056 | ADIPOQ,CXCL12,TNF,IL6,VEGFA,IL10,IL4,AKT1,CTNNB1,PIK3CA |
| GO:BP | neurogenesis | GO:0022008 | 7.29E-06 | 5.136996106 | NGF,CXCL12,TNF,IL6,VEGFA,MAPK3,EDN1,AKT1,TP53,CTNNB1,GJA1,EP300,SOD1,AGT |
| GO:BP | in utero embryonic development | GO:0001701 | 7.37E-06 | 5.132457717 | NOS3,VEGFA,IL10,EDN1,AKT1,TP53,CTNNB1,GJA1 |
| GO:BP | myeloid leukocyte migration | GO:0097529 | 7.39E-06 | 5.131443228 | CXCL12,IL6,VEGFA,MAPK3,EDN1,IL4 |
| GO:BP | regulation of receptor signaling pathway via STAT | GO:1904892 | 7.79E-06 | 5.108556378 | TNF,IL6,IL10,EGF,IL4,AGT |
| GO:BP | regulation of proteolysis | GO:0030162 | 8.22E-06 | 5.085244788 | NGF,TNF,VEGFA,IL10,PTGS2,EGF,AKT1,TP53,SIRT1 |
| GO:BP | ossification | GO:0001503 | 8.28E-06 | 5.081969602 | TNF,IL6,MAPK3,PTGS2,AKT1,CAT,CTNNB1,GJA1 |
| GO:BP | mononuclear cell migration | GO:0071674 | 8.28E-06 | 5.081782463 | CXCL12,TNF,IL6,MAPK3,IL4,AKT1 |
| GO:BP | hexose metabolic process | GO:0019318 | 8.41E-06 | 5.075134269 | ADIPOQ,TNF,AKT1,TP53,SIRT1,PIK3CA,EP300 |
| GO:BP | positive regulation of receptor signaling pathway via STAT | GO:1904894 | 8.44E-06 | 5.073885197 | TNF,IL6,IL10,IL4,AGT |
| GO:BP | regulation of generation of precursor metabolites and energy | GO:0043467 | 8.61E-06 | 5.06521144 | TNF,IL4,AKT1,TP53,PIK3CA,EP300 |
| GO:BP | anatomical structure homeostasis | GO:0060249 | 8.74E-06 | 5.058634793 | NOS3,ALB,IL6,VEGFA,PTGS2,CTNNB1,GJA1,SOD1 |
| GO:BP | tissue morphogenesis | GO:0048729 | 8.81E-06 | 5.054893007 | NOS3,TNF,VEGFA,IL10,EDN1,EGF,ESR1,CTNNB1,GJA1,AGT |
| GO:BP | hemopoiesis | GO:0030097 | 9.17E-06 | 5.037628356 | ADIPOQ,TNF,IL6,VEGFA,IL10,IL4,TP53,SIRT1,CTNNB1,EP300,SOD1 |
| GO:BP | organic cyclic compound biosynthetic process | GO:1901362 | 9.39E-06 | 5.027111377 | ADIPOQ,NOS3,TNF,IL6,VEGFA,MAPK3,IL10,EDN1,EGF,IL4,ESR1,AKT1,CAT,TP53,SIRT1,CTNNB1,EP300,ESR2,SOD1,AGT |
| GO:BP | nucleobase-containing compound biosynthetic process | GO:0034654 | 9.45E-06 | 5.024495547 | ADIPOQ,NOS3,TNF,IL6,VEGFA,MAPK3,IL10,EDN1,EGF,IL4,ESR1,AKT1,CAT,TP53,SIRT1,CTNNB1 |
| GO:BP | regulation of neuron apoptotic process | GO:0043523 | 9.48E-06 | 5.023089392 | NGF,TNF,IL10,TP53,CTNNB1,PIK3CA,SOD1 |
| GO:BP | cellular nitrogen compound biosynthetic process | GO:0044271 | 9.55E-06 | 5.020175064 | ADIPOQ,NOS3,TNF,IL6,VEGFA,MAPK3,IL10,PTGS2,EDN1,EGF,IL4,ESR1,AKT1,CAT,TP53,SIRT1,CTNNB1 |
| GO:BP | positive regulation of cell adhesion | GO:0045785 | 9.69E-06 | 5.013482975 | CXCL12,TNF,IL6,VEGFA,IL10,IL4,AKT1,PIK3CA |
| GO:BP | regulation of mitotic cell cycle | GO:0007346 | 9.75E-06 | 5.01084443 | TNF,IL10,EDN1,EGF,AKT1,TP53,SIRT1,CTNNB1 |
| GO:BP | negative regulation of neuron death | GO:1901215 | 9.81E-06 | 5.008289186 | NGF,IL10,AKT1,SIRT1,CTNNB1,PIK3CA,SOD1 |
| GO:BP | localization | GO:0051179 | 1.02E-05 | 4.990173101 | ADIPOQ,NOS3,CXCL12,ALB,TNF,IL6,VEGFA,MAPK3,IL10,PTGS2,EDN1,EGF,IL4,ESR1,AKT1,TP53,SIRT1,CTNNB1,PIK3CA,GJA1 |
| GO:BP | negative regulation of transport | GO:0051051 | 1.06E-05 | 4.975186046 | ADIPOQ,NOS3,TNF,PTGS2,EDN1,EGF,AKT1,GJA1 |
| GO:BP | transport | GO:0006810 | 1.06E-05 | 4.974933105 | ADIPOQ,NOS3,CXCL12,TNF,IL6,VEGFA,MAPK3,IL10,PTGS2,EDN1,EGF,IL4,AKT1,TP53,SIRT1,CTNNB1,PIK3CA,GJA1,CYBB,SOD1,AGT |
| GO:BP | regulation of phosphatidylinositol 3-kinase signaling | GO:0014066 | 1.09E-05 | 4.961205505 | TNF,VEGFA,EGF,CAT,SIRT1,AGT |
| GO:BP | regulation of macroautophagy | GO:0016241 | 1.10E-05 | 4.958696072 | MAPK3,IL4,AKT1,TP53,SIRT1,PIK3CA |
| GO:BP | cellular response to lipopolysaccharide | GO:0071222 | 1.11E-05 | 4.952913194 | NOS3,TNF,IL6,MAPK3,IL10,AKT1 |
| GO:BP | negative regulation of cell population proliferation | GO:0008285 | 1.12E-05 | 4.949782673 | NGF,ADIPOQ,NOS3,TNF,IL6,IL10,PTGS2,TP53,CTNNB1 |
| GO:BP | heterocycle biosynthetic process | GO:0018130 | 1.24E-05 | 4.907265641 | ADIPOQ,NOS3,TNF,IL6,VEGFA,MAPK3,IL10,EDN1,EGF,IL4,ESR1,AKT1,CAT,TP53,SIRT1,CTNNB1 |
| GO:BP | positive regulation of neurogenesis | GO:0050769 | 1.25E-05 | 4.903274326 | NGF,CXCL12,TNF,IL6,VEGFA |
| GO:BP | negative regulation of proteolysis | GO:0045861 | 1.27E-05 | 4.895684646 | NGF,TNF,VEGFA,IL10,PTGS2,AKT1,TP53 |
| GO:BP | regulation of morphogenesis of a branching structure | GO:0060688 | 1.28E-05 | 4.894251931 | TNF,VEGFA,ESR1,CTNNB1,AGT |
| GO:BP | aromatic compound biosynthetic process | GO:0019438 | 1.29E-05 | 4.889301764 | ADIPOQ,NOS3,TNF,IL6,VEGFA,MAPK3,IL10,EDN1,EGF,IL4,ESR1,AKT1,CAT,TP53,SIRT1,CTNNB1 |
| GO:BP | T cell activation | GO:0042110 | 1.29E-05 | 4.888491435 | IL6,IL10,IL4,AKT1,TP53,CTNNB1,PIK3CA,GJA1,SOD1 |
| GO:BP | regulation of MAPK cascade | GO:0043408 | 1.37E-05 | 4.864338303 | ADIPOQ,TNF,IL6,VEGFA,MAPK3,EDN1,EGF,CTNNB1,SOD1,AGT |
| GO:BP | cellular detoxification | GO:1990748 | 1.37E-05 | 4.862378823 | NOS3,ALB,PTGS2,CAT,SOD1,MPO |
| GO:BP | epithelial tube morphogenesis | GO:0060562 | 1.43E-05 | 4.845612072 | TNF,VEGFA,EDN1,EGF,ESR1,CTNNB1,GJA1,AGT |
| GO:BP | cellular response to molecule of bacterial origin | GO:0071219 | 1.44E-05 | 4.842283096 | NOS3,TNF,IL6,MAPK3,IL10,AKT1 |
| GO:BP | regulation of cellular process | GO:0050794 | 1.44E-05 | 4.840618142 | NGF,ADIPOQ,NOS3,CXCL12,ALB,TNF,IL6,VEGFA,MAPK3,IL10,PTGS2,EDN1,EGF,IL4,ESR1,AKT1,CAT,TP53,SIRT1,CTNNB1,PIK3CA,GJA1,EP300,CYBB,MMP3,ESR2,SOD1,AGT,MPO |
| GO:BP | monosaccharide metabolic process | GO:0005996 | 1.47E-05 | 4.833652908 | ADIPOQ,TNF,AKT1,TP53,SIRT1,PIK3CA,EP300 |
| GO:BP | response to hydrogen peroxide | GO:0042542 | 1.61E-05 | 4.792753473 | IL6,IL10,EDN1,CAT,SIRT1,SOD1 |
| GO:BP | cellular response to inorganic substance | GO:0071241 | 1.65E-05 | 4.781243249 | MAPK3,PTGS2,EDN1,AKT1,CYBB,MMP3,SOD1 |
| GO:BP | regulation of transcription, DNA-templated | GO:0006355 | 1.69E-05 | 4.772454602 | ADIPOQ,TNF,IL6,VEGFA,MAPK3,IL10,EDN1,EGF,IL4,ESR1,AKT1,CAT,TP53,SIRT1,CTNNB1 |
| GO:BP | regulation of nucleic acid-templated transcription | GO:1903506 | 1.70E-05 | 4.770655496 | ADIPOQ,TNF,IL6,VEGFA,MAPK3,IL10,EDN1,EGF,IL4,ESR1,AKT1,CAT,TP53,SIRT1,CTNNB1 |
| GO:BP | hydrogen peroxide metabolic process | GO:0042743 | 1.70E-05 | 4.770253724 | CAT,CYBB,MMP3,SOD1,MPO |
| GO:BP | regulation of RNA biosynthetic process | GO:2001141 | 1.73E-05 | 4.761668468 | ADIPOQ,TNF,IL6,VEGFA,MAPK3,IL10,EDN1,EGF,IL4,ESR1,AKT1,CAT,TP53,SIRT1,CTNNB1 |
| GO:BP | response to cadmium ion | GO:0046686 | 1.84E-05 | 4.734881083 | MAPK3,AKT1,CAT,CYBB,SOD1 |
| GO:BP | establishment of localization | GO:0051234 | 1.99E-05 | 4.7009559 | ADIPOQ,NOS3,CXCL12,TNF,IL6,VEGFA,MAPK3,IL10,PTGS2,EDN1,EGF,IL4,AKT1,TP53,SIRT1,CTNNB1,PIK3CA,GJA1,CYBB,SOD1,AGT |
| GO:BP | cellular response to cytokine stimulus | GO:0071345 | 2.52E-05 | 4.598710353 | ADIPOQ,CXCL12,TNF,IL6,MAPK3,EDN1,AKT1,TP53,SIRT1 |
| GO:BP | regulation of cell adhesion | GO:0030155 | 2.61E-05 | 4.583892712 | ADIPOQ,CXCL12,TNF,IL6,VEGFA,IL10,IL4,AKT1,PIK3CA |
| GO:BP | peptidyl-serine phosphorylation | GO:0018105 | 2.63E-05 | 4.580364952 | NGF,TNF,IL6,VEGFA,PTGS2,AKT1,PIK3CA |
| GO:BP | regulation of cytokine production | GO:0001817 | 2.72E-05 | 4.566148603 | ADIPOQ,TNF,IL6,IL10,PTGS2,IL4,SIRT1,CYBB,SOD1,AGT |
| GO:BP | neuron apoptotic process | GO:0051402 | 2.77E-05 | 4.55812022 | NGF,TNF,IL10,TP53,CTNNB1,PIK3CA,SOD1 |
| GO:BP | chordate embryonic development | GO:0043009 | 2.79E-05 | 4.554293159 | NOS3,VEGFA,IL10,EDN1,AKT1,TP53,CTNNB1,GJA1,EP300 |
| GO:BP | regulation of protein serine/threonine kinase activity | GO:0071900 | 2.93E-05 | 4.532635679 | ADIPOQ,TNF,VEGFA,EDN1,EGF,AKT1,SIRT1,AGT |
| GO:BP | cytokine production | GO:0001816 | 2.94E-05 | 4.531863534 | ADIPOQ,TNF,IL6,IL10,PTGS2,IL4,SIRT1,CYBB,SOD1,AGT |
| GO:BP | transcription, DNA-templated | GO:0006351 | 2.97E-05 | 4.527680619 | ADIPOQ,TNF,IL6,VEGFA,MAPK3,IL10,EDN1,EGF,IL4,ESR1,AKT1,CAT,TP53,SIRT1,CTNNB1 |
| GO:BP | nucleic acid-templated transcription | GO:0097659 | 2.98E-05 | 4.525957345 | ADIPOQ,TNF,IL6,VEGFA,MAPK3,IL10,EDN1,EGF,IL4,ESR1,AKT1,CAT,TP53,SIRT1,CTNNB1 |
| GO:BP | response to alcohol | GO:0097305 | 3.02E-05 | 4.520482303 | ADIPOQ,TNF,AKT1,CAT,CTNNB1,CYBB,SOD1 |
| GO:BP | RNA biosynthetic process | GO:0032774 | 3.17E-05 | 4.49845606 | ADIPOQ,TNF,IL6,VEGFA,MAPK3,IL10,EDN1,EGF,IL4,ESR1,AKT1,CAT,TP53,SIRT1,CTNNB1 |
| GO:BP | morphogenesis of an epithelium | GO:0002009 | 3.24E-05 | 4.489383873 | TNF,VEGFA,IL10,EDN1,EGF,ESR1,CTNNB1,GJA1,AGT |
| GO:BP | cellular response to hypoxia | GO:0071456 | 3.26E-05 | 4.487188472 | VEGFA,PTGS2,EDN1,TP53,SIRT1,CYBB |
| GO:BP | ERK1 and ERK2 cascade | GO:0070371 | 3.26E-05 | 4.487086839 | ADIPOQ,TNF,VEGFA,MAPK3,EDN1,EGF |
| GO:BP | generation of neurons | GO:0048699 | 3.32E-05 | 4.479452098 | NGF,CXCL12,TNF,IL6,VEGFA,EDN1,AKT1,TP53,CTNNB1,GJA1,EP300,SOD1,AGT |
| GO:BP | positive regulation of nervous system development | GO:0051962 | 3.38E-05 | 4.470955924 | NGF,CXCL12,TNF,IL6,VEGFA |
| GO:BP | regulation of cellular localization | GO:0060341 | 3.46E-05 | 4.460321103 | ADIPOQ,TNF,VEGFA,MAPK3,PTGS2,EGF,IL4,AKT1,CTNNB1 |
| GO:BP | regulation of mononuclear cell migration | GO:0071675 | 3.48E-05 | 4.458490703 | CXCL12,TNF,MAPK3,IL4,AKT1 |
| GO:BP | developmental process involved in reproduction | GO:0003006 | 3.51E-05 | 4.45462591 | NOS3,VEGFA,IL10,PTGS2,EDN1,ESR1,AKT1,SIRT1,CTNNB1,GJA1,SOD1 |
| GO:BP | heart contraction | GO:0060047 | 3.65E-05 | 4.437349579 | NOS3,TNF,EDN1,PIK3CA,GJA1,SOD1,AGT |
| GO:BP | embryo development ending in birth or egg hatching | GO:0009792 | 3.66E-05 | 4.436917718 | NOS3,VEGFA,IL10,EDN1,AKT1,TP53,CTNNB1,GJA1,EP300 |
| GO:BP | animal organ regeneration | GO:0031100 | 3.69E-05 | 4.432430592 | CXCL12,TNF,IL6,IL10 |
| GO:BP | regulation of leukocyte cell-cell adhesion | GO:1903037 | 3.70E-05 | 4.431459958 | CXCL12,TNF,IL6,IL10,IL4,AKT1,PIK3CA |
| GO:BP | cytokine production involved in immune response | GO:0002367 | 3.71E-05 | 4.430395369 | TNF,IL6,IL10,IL4,SIRT1 |
| GO:BP | regulation of cytokine production involved in immune response | GO:0002718 | 3.71E-05 | 4.430395369 | TNF,IL6,IL10,IL4,SIRT1 |
| GO:BP | small molecule metabolic process | GO:0044281 | 3.75E-05 | 4.426364478 | ADIPOQ,NOS3,TNF,PTGS2,EDN1,IL4,AKT1,CAT,TP53,SIRT1,PIK3CA,EP300,SOD1,MPO |
| GO:BP | regulation of acute inflammatory response | GO:0002673 | 3.85E-05 | 4.41449423 | TNF,IL6,PTGS2,IL4 |
| GO:BP | temperature homeostasis | GO:0001659 | 3.89E-05 | 4.40990476 | ADIPOQ,TNF,VEGFA,PTGS2,IL4,GJA1 |
| GO:BP | regulation of lipid transport | GO:0032368 | 3.92E-05 | 4.406812739 | ADIPOQ,EDN1,EGF,AKT1,SIRT1,AGT |
| GO:BP | rhythmic process | GO:0048511 | 4.13E-05 | 4.384051055 | ADIPOQ,NOS3,TNF,ESR1,TP53,SIRT1,EP300 |
| GO:BP | response to toxic substance | GO:0009636 | 4.24E-05 | 4.372360479 | NOS3,ALB,TNF,PTGS2,CAT,SOD1,MPO |
| GO:BP | regulation of tube diameter | GO:0035296 | 4.28E-05 | 4.368840073 | NOS3,TNF,PTGS2,EDN1,SOD1,AGT |
| GO:BP | blood vessel diameter maintenance | GO:0097746 | 4.28E-05 | 4.368840073 | NOS3,TNF,PTGS2,EDN1,SOD1,AGT |
| GO:BP | peptidyl-serine modification | GO:0018209 | 4.28E-05 | 4.368662249 | NGF,TNF,IL6,VEGFA,PTGS2,AKT1,PIK3CA |
| GO:BP | lymphocyte activation | GO:0046649 | 4.35E-05 | 4.361118254 | IL6,IL10,IL4,AKT1,TP53,CTNNB1,PIK3CA,GJA1,EP300,SOD1 |
| GO:BP | regulation of tube size | GO:0035150 | 4.47E-05 | 4.350067807 | NOS3,TNF,PTGS2,EDN1,SOD1,AGT |
| GO:BP | regulation of neurogenesis | GO:0050767 | 4.47E-05 | 4.349514734 | NGF,CXCL12,TNF,IL6,VEGFA,TP53,CTNNB1 |
| GO:BP | regulation of system process | GO:0044057 | 4.52E-05 | 4.344779249 | ADIPOQ,NOS3,TNF,IL10,PTGS2,EDN1,GJA1,SOD1,AGT |
| GO:BP | positive regulation of histone modification | GO:0031058 | 4.68E-05 | 4.329766863 | VEGFA,MAPK3,TP53,SIRT1,CTNNB1 |
| GO:BP | detoxification | GO:0098754 | 4.70E-05 | 4.327633954 | NOS3,ALB,PTGS2,CAT,SOD1,MPO |
| GO:BP | heart process | GO:0003015 | 4.85E-05 | 4.313945756 | NOS3,TNF,EDN1,PIK3CA,GJA1,SOD1,AGT |
| GO:BP | leukocyte proliferation | GO:0070661 | 4.93E-05 | 4.307411315 | IL6,MAPK3,IL10,IL4,TP53,CTNNB1,GJA1 |
| GO:BP | negative regulation of RNA metabolic process | GO:0051253 | 4.99E-05 | 4.301595342 | ADIPOQ,TNF,IL6,VEGFA,EDN1,IL4,ESR1,TP53,SIRT1,CTNNB1,EP300,ESR2 |
| GO:BP | regulation of chronic inflammatory response | GO:0002676 | 5.02E-05 | 4.299341161 | TNF,IL10,IL4 |
| GO:BP | regulation of RNA metabolic process | GO:0051252 | 5.29E-05 | 4.276540606 | ADIPOQ,TNF,IL6,VEGFA,MAPK3,IL10,EDN1,EGF,IL4,ESR1,AKT1,CAT,TP53,SIRT1,CTNNB1 |
| GO:BP | lung development | GO:0030324 | 5.59E-05 | 4.252565697 | NOS3,TNF,VEGFA,MAPK3,CTNNB1,EP300 |
| GO:BP | secretion | GO:0046903 | 5.71E-05 | 4.243239398 | ADIPOQ,CXCL12,TNF,IL6,VEGFA,EDN1,EGF,IL4 |
| GO:BP | regulation of carbohydrate metabolic process | GO:0006109 | 5.78E-05 | 4.23814497 | ADIPOQ,EGF,AKT1,TP53,SIRT1,EP300 |
| GO:BP | negative regulation of lipid localization | GO:1905953 | 5.83E-05 | 4.23401971 | TNF,IL6,EGF,AKT1 |
| GO:BP | myeloid leukocyte differentiation | GO:0002573 | 5.85E-05 | 4.232844583 | ADIPOQ,TNF,VEGFA,IL4,SIRT1,CTNNB1 |
| GO:BP | heart development | GO:0007507 | 5.86E-05 | 4.231919058 | NOS3,VEGFA,MAPK3,EDN1,TP53,CTNNB1,GJA1,EP300,AGT |
| GO:BP | response to insulin | GO:0032868 | 5.88E-05 | 4.230629518 | ADIPOQ,IL10,AKT1,CAT,SIRT1,PIK3CA,AGT |
| GO:BP | protein kinase B signaling | GO:0043491 | 5.91E-05 | 4.22865923 | TNF,VEGFA,EGF,AKT1,SIRT1,PIK3CA |
| GO:BP | respiratory tube development | GO:0030323 | 6.38E-05 | 4.19537554 | NOS3,TNF,VEGFA,MAPK3,CTNNB1,EP300 |
| GO:BP | organic substance biosynthetic process | GO:1901576 | 6.56E-05 | 4.183164313 | ADIPOQ,NOS3,TNF,IL6,VEGFA,MAPK3,IL10,PTGS2,EDN1,EGF,IL4,ESR1,AKT1,CAT,TP53,SIRT1,CTNNB1,PIK3CA,EP300,ESR2,SOD1,AGT |
| GO:BP | vascular process in circulatory system | GO:0003018 | 6.72E-05 | 4.172574286 | NOS3,TNF,VEGFA,PTGS2,EDN1,SOD1,AGT |
| GO:BP | MAPK cascade | GO:0000165 | 6.95E-05 | 4.158144994 | ADIPOQ,TNF,IL6,VEGFA,MAPK3,EDN1,EGF,CTNNB1,SOD1,AGT |
| GO:BP | cellular response to hormone stimulus | GO:0032870 | 7.01E-05 | 4.154384036 | ADIPOQ,EDN1,ESR1,AKT1,SIRT1,PIK3CA,EP300,ESR2,AGT |
| GO:BP | negative regulation of cell adhesion | GO:0007162 | 7.19E-05 | 4.143000023 | ADIPOQ,CXCL12,VEGFA,IL10,IL4,AKT1 |
| GO:BP | cellular protein modification process | GO:0006464 | 7.50E-05 | 4.124717345 | NGF,ADIPOQ,TNF,IL6,VEGFA,MAPK3,PTGS2,EDN1,EGF,IL4,AKT1,TP53,SIRT1,CTNNB1,PIK3CA,EP300,SOD1,AGT |
| GO:BP | protein modification process | GO:0036211 | 7.50E-05 | 4.124717345 | NGF,ADIPOQ,TNF,IL6,VEGFA,MAPK3,PTGS2,EDN1,EGF,IL4,AKT1,TP53,SIRT1,CTNNB1,PIK3CA,EP300,SOD1,AGT |
| GO:BP | leukocyte cell-cell adhesion | GO:0007159 | 7.83E-05 | 4.105990243 | CXCL12,TNF,IL6,IL10,IL4,AKT1,PIK3CA |
| GO:BP | regulation of secretion | GO:0051046 | 8.10E-05 | 4.091320025 | ADIPOQ,CXCL12,TNF,IL6,EDN1,EGF,IL4 |
| GO:BP | maintenance of blood-brain barrier | GO:0035633 | 8.22E-05 | 4.085138946 | IL6,VEGFA,PTGS2,GJA1 |
| GO:BP | multi-organism reproductive process | GO:0044703 | 8.31E-05 | 4.08050822 | NOS3,VEGFA,PTGS2,EDN1,ESR1,AKT1,SIRT1,CTNNB1,GJA1,SOD1,AGT |
| GO:BP | receptor signaling pathway via JAK-STAT | GO:0007259 | 8.36E-05 | 4.077941228 | TNF,IL6,IL10,EGF,IL4 |
| GO:BP | chemotaxis | GO:0006935 | 8.48E-05 | 4.071704738 | CXCL12,IL6,VEGFA,MAPK3,IL10,EDN1,IL4 |
| GO:BP | negative regulation of developmental process | GO:0051093 | 8.48E-05 | 4.071494781 | ADIPOQ,NOS3,TNF,IL6,VEGFA,IL4,TP53,SIRT1,CTNNB1 |
| GO:BP | cell cycle | GO:0007049 | 8.48E-05 | 4.071394793 | TNF,MAPK3,IL10,PTGS2,EDN1,EGF,AKT1,TP53,SIRT1,CTNNB1,GJA1,EP300 |
| GO:BP | cell-cell signaling | GO:0007267 | 8.64E-05 | 4.063438208 | NGF,ADIPOQ,CXCL12,TNF,IL6,PTGS2,EDN1,EGF,AKT1,CTNNB1,GJA1,ESR2,AGT |
| GO:BP | regulation of interleukin-8 production | GO:0032677 | 8.72E-05 | 4.05940534 | ADIPOQ,TNF,IL6,IL10 |
| GO:BP | interleukin-8 production | GO:0032637 | 8.72E-05 | 4.05940534 | ADIPOQ,TNF,IL6,IL10 |
| GO:BP | taxis | GO:0042330 | 8.87E-05 | 4.05222199 | CXCL12,IL6,VEGFA,MAPK3,IL10,EDN1,IL4 |
| GO:BP | T cell proliferation | GO:0042098 | 9.06E-05 | 4.042687025 | IL6,IL10,IL4,TP53,CTNNB1,GJA1 |
| GO:BP | lipid metabolic process | GO:0006629 | 9.28E-05 | 4.03228089 | ADIPOQ,TNF,PTGS2,EDN1,IL4,ESR1,AKT1,CAT,SIRT1,PIK3CA,SOD1,AGT |
| GO:BP | sex differentiation | GO:0007548 | 9.35E-05 | 4.029250341 | NOS3,VEGFA,ESR1,SIRT1,CTNNB1,GJA1,SOD1 |
| GO:BP | mononuclear cell differentiation | GO:1903131 | 9.47E-05 | 4.023440957 | IL6,VEGFA,IL10,IL4,TP53,CTNNB1,EP300,SOD1 |
| GO:BP | regulation of protein localization to cell surface | GO:2000008 | 9.50E-05 | 4.022458485 | TNF,EGF,AKT1,CTNNB1 |
| GO:BP | muscle system process | GO:0003012 | 9.61E-05 | 4.017285175 | NOS3,TNF,PTGS2,EDN1,PIK3CA,GJA1,SOD1,AGT |
| GO:BP | regulation of gene silencing by miRNA | GO:0060964 | 9.78E-05 | 4.009725334 | TNF,IL6,ESR1,TP53 |
| GO:BP | epithelial cell development | GO:0002064 | 9.88E-05 | 4.005029658 | ADIPOQ,TNF,VEGFA,ESR1,AKT1,GJA1 |
| GO:BP | regulation of MAP kinase activity | GO:0043405 | 9.99E-05 | 4.00064791 | ADIPOQ,TNF,VEGFA,EDN1,EGF |
| GO:BP | receptor signaling pathway via STAT | GO:0097696 | 0.000100361 | 3.998435241 | TNF,IL6,IL10,EGF,IL4 |
| GO:BP | regulation of carbohydrate biosynthetic process | GO:0043255 | 0.000100475 | 3.997941658 | ADIPOQ,EGF,AKT1,SIRT1,EP300 |
| GO:BP | response to light stimulus | GO:0009416 | 0.000100995 | 3.995700467 | PTGS2,AKT1,CAT,TP53,SIRT1,EP300,MMP3 |
| GO:BP | autophagy | GO:0006914 | 0.000101208 | 3.994784294 | MAPK3,IL10,IL4,AKT1,TP53,SIRT1,PIK3CA,EP300 |
| GO:BP | process utilizing autophagic mechanism | GO:0061919 | 0.000101208 | 3.994784294 | MAPK3,IL10,IL4,AKT1,TP53,SIRT1,PIK3CA,EP300 |
| GO:BP | regulation of intrinsic apoptotic signaling pathway | GO:2001242 | 0.000105527 | 3.976634364 | CXCL12,PTGS2,AKT1,TP53,SIRT1,SOD1 |
| GO:BP | cellular response to UV | GO:0034644 | 0.000107553 | 3.968378424 | PTGS2,TP53,SIRT1,EP300,MMP3 |
| GO:BP | negative regulation of nucleobase-containing compound metabolic process | GO:0045934 | 0.000108508 | 3.964536342 | ADIPOQ,TNF,IL6,VEGFA,EDN1,IL4,ESR1,TP53,SIRT1,CTNNB1,EP300,ESR2 |
| GO:BP | transmembrane receptor protein tyrosine kinase signaling pathway | GO:0007169 | 0.000110749 | 3.955661557 | NGF,ADIPOQ,VEGFA,EGF,AKT1,SIRT1,CTNNB1,PIK3CA,AGT |
| GO:BP | protein metabolic process | GO:0019538 | 0.000111429 | 3.953003009 | NGF,ADIPOQ,TNF,IL6,VEGFA,MAPK3,IL10,PTGS2,EDN1,EGF,IL4,AKT1,CAT,TP53,SIRT1,CTNNB1,PIK3CA,EP300,MMP3,SOD1,AGT |
| GO:BP | negative regulation of production of miRNAs involved in gene silencing by miRNA | GO:1903799 | 0.000112412 | 3.949186313 | TNF,ESR1,TP53 |
| GO:BP | developmental growth involved in morphogenesis | GO:0060560 | 0.000113768 | 3.943981029 | NGF,CXCL12,VEGFA,EDN1,ESR1,CTNNB1 |
| GO:BP | positive regulation of cellular catabolic process | GO:0031331 | 0.000115677 | 3.936751335 | TNF,IL6,MAPK3,EGF,IL4,AKT1,SIRT1 |
| GO:BP | regulation of posttranscriptional gene silencing | GO:0060147 | 0.000116936 | 3.932050544 | TNF,IL6,ESR1,TP53 |
| GO:BP | cellular component organization | GO:0016043 | 0.000119356 | 3.923157314 | NGF,ADIPOQ,NOS3,CXCL12,TNF,IL6,VEGFA,MAPK3,IL10,EDN1,EGF,IL4,ESR1,AKT1,TP53,SIRT1,CTNNB1,PIK3CA,GJA1,EP300,MMP3,ESR2,SOD1,AGT,MPO |
| GO:BP | negative regulation of immune system process | GO:0002683 | 0.000120909 | 3.917540232 | ADIPOQ,CXCL12,TNF,IL10,IL4,AKT1,CTNNB1 |
| GO:BP | lipid localization | GO:0010876 | 0.000121208 | 3.916469569 | ADIPOQ,TNF,IL6,EDN1,EGF,AKT1,SIRT1 |
| GO:BP | regulation of macromolecule biosynthetic process | GO:0010556 | 0.000121378 | 3.915859628 | ADIPOQ,TNF,IL6,VEGFA,MAPK3,IL10,EDN1,EGF,IL4,ESR1,AKT1,CAT,TP53,SIRT1,CTNNB1 |
| GO:BP | respiratory system development | GO:0060541 | 0.000121641 | 3.914920945 | NOS3,TNF,VEGFA,MAPK3,CTNNB1,EP300 |
| GO:BP | nervous system development | GO:0007399 | 0.000126989 | 3.896232348 | NGF,CXCL12,TNF,IL6,VEGFA,MAPK3,EDN1,EGF,AKT1,TP53,CTNNB1,GJA1,EP300,SOD1,AGT |
| GO:BP | regulation of gene silencing by RNA | GO:0060966 | 0.000127499 | 3.894493565 | TNF,IL6,ESR1,TP53 |
| GO:BP | tissue remodeling | GO:0048771 | 0.00012875 | 3.890252773 | NOS3,IL6,TP53,CTNNB1,GJA1,AGT |
| GO:BP | negative regulation of macromolecule biosynthetic process | GO:0010558 | 0.000132237 | 3.878647861 | ADIPOQ,TNF,VEGFA,IL10,EDN1,IL4,ESR1,TP53,SIRT1,CTNNB1,EP300,ESR2 |
| GO:BP | chemokine production | GO:0032602 | 0.000137526 | 3.861615701 | ADIPOQ,TNF,IL6,IL10 |
| GO:BP | regulation of chemokine production | GO:0032642 | 0.000137526 | 3.861615701 | ADIPOQ,TNF,IL6,IL10 |
| GO:BP | regulation of cell activation | GO:0050865 | 0.00013933 | 3.855955884 | NOS3,TNF,IL6,IL10,IL4,AKT1,CTNNB1,PIK3CA,SOD1 |
| GO:BP | negative regulation of cell differentiation | GO:0045596 | 0.000140374 | 3.852714541 | ADIPOQ,TNF,IL6,VEGFA,IL4,TP53,SIRT1,CTNNB1 |
| GO:BP | negative regulation of transcription by RNA polymerase II | GO:0000122 | 0.000143965 | 3.841742001 | TNF,VEGFA,EDN1,IL4,ESR1,TP53,SIRT1,CTNNB1,EP300,ESR2 |
| GO:BP | regulation of leukocyte adhesion to vascular endothelial cell | GO:1904994 | 0.000146823 | 3.833205882 | CXCL12,TNF,IL6 |
| GO:BP | production of miRNAs involved in gene silencing by miRNA | GO:0035196 | 0.000150744 | 3.821761004 | TNF,IL6,ESR1,TP53 |
| GO:BP | production of small RNA involved in gene silencing by RNA | GO:0070918 | 0.000163486 | 3.78652028 | TNF,IL6,ESR1,TP53 |
| GO:BP | dsRNA processing | GO:0031050 | 0.000163486 | 3.78652028 | TNF,IL6,ESR1,TP53 |
| GO:BP | osteoblast differentiation | GO:0001649 | 0.00016637 | 3.77892578 | TNF,IL6,AKT1,CAT,CTNNB1,GJA1 |
| GO:BP | negative regulation of oxidative stress-induced cell death | GO:1903202 | 0.000168941 | 3.772264662 | IL10,AKT1,SIRT1,CTNNB1 |
| GO:BP | negative regulation of response to external stimulus | GO:0032102 | 0.000171584 | 3.765523559 | ADIPOQ,NOS3,TNF,IL10,EDN1,IL4 |
| GO:BP | intrinsic apoptotic signaling pathway in response to DNA damage | GO:0008630 | 0.00017338 | 3.761001862 | CXCL12,TNF,TP53,SIRT1,EP300 |
| GO:BP | regulation of organelle organization | GO:0033043 | 0.000173919 | 3.759652148 | CXCL12,TNF,MAPK3,EDN1,EGF,AKT1,TP53,CTNNB1,PIK3CA,EP300 |
| GO:BP | positive regulation of cell growth | GO:0030307 | 0.000180093 | 3.744503094 | NGF,CXCL12,VEGFA,EDN1,AKT1 |
| GO:BP | positive regulation of MHC class II biosynthetic process | GO:0045348 | 0.000183398 | 3.736606416 | IL10,IL4,SIRT1 |
| GO:BP | macromolecule modification | GO:0043412 | 0.000186577 | 3.729141092 | NGF,ADIPOQ,TNF,IL6,VEGFA,MAPK3,PTGS2,EDN1,EGF,IL4,AKT1,TP53,SIRT1,CTNNB1,PIK3CA,EP300 |
| GO:BP | positive regulation of MAPK cascade | GO:0043410 | 0.000191237 | 3.718428732 | TNF,IL6,VEGFA,MAPK3,EDN1,EGF,CTNNB1,SOD1 |
| GO:BP | positive regulation of developmental growth | GO:0048639 | 0.000191541 | 3.717737687 | NGF,CXCL12,VEGFA,EDN1,AKT1 |
| GO:BP | negative regulation of cellular biosynthetic process | GO:0031327 | 0.000199672 | 3.699681897 | ADIPOQ,TNF,VEGFA,IL10,EDN1,IL4,ESR1,TP53,SIRT1,CTNNB1,EP300,ESR2 |
| GO:BP | lipopolysaccharide-mediated signaling pathway | GO:0031663 | 0.000202853 | 3.692818219 | NOS3,TNF,MAPK3,AKT1 |
| GO:BP | regulation of developmental growth | GO:0048638 | 0.000211514 | 3.674661228 | NGF,CXCL12,VEGFA,EDN1,AKT1,PIK3CA,SOD1 |
| GO:BP | regulation of biological process | GO:0050789 | 0.000212983 | 3.671656001 | NGF,ADIPOQ,NOS3,CXCL12,ALB,TNF,IL6,VEGFA,MAPK3,IL10,PTGS2,EDN1,EGF,IL4,ESR1,AKT1,CAT,TP53,SIRT1,CTNNB1,PIK3CA,GJA1,EP300,CYBB,MMP3,ESR2,SOD1,AGT,MPO |
| GO:BP | cellular component organization or biogenesis | GO:0071840 | 0.000213238 | 3.671135358 | NGF,ADIPOQ,NOS3,CXCL12,TNF,IL6,VEGFA,MAPK3,IL10,EDN1,EGF,IL4,ESR1,AKT1,TP53,SIRT1,CTNNB1,PIK3CA,GJA1,EP300,MMP3,ESR2,SOD1,AGT,MPO |
| GO:BP | cellular response to cadmium ion | GO:0071276 | 0.00022036 | 3.656866274 | MAPK3,AKT1,CYBB,SOD1 |
| GO:BP | cellular homeostasis | GO:0019725 | 0.000220618 | 3.656358086 | NOS3,CXCL12,IL6,MAPK3,EDN1,ESR1,SIRT1,PIK3CA,SOD1,AGT |
| GO:BP | cellular response to metal ion | GO:0071248 | 0.000226892 | 3.644180462 | MAPK3,PTGS2,EDN1,AKT1,CYBB,SOD1 |
| GO:BP | mitotic cell cycle | GO:0000278 | 0.000237103 | 3.625063768 | TNF,IL10,EDN1,EGF,AKT1,TP53,SIRT1,CTNNB1,GJA1 |
| GO:BP | negative regulation of fat cell differentiation | GO:0045599 | 0.000241942 | 3.616289281 | ADIPOQ,TNF,IL6,SIRT1 |
| GO:BP | regulation of cell cycle | GO:0051726 | 0.000245819 | 3.609384498 | TNF,IL10,PTGS2,EDN1,EGF,AKT1,TP53,SIRT1,CTNNB1 |
| GO:BP | regulation of immunoglobulin production | GO:0002637 | 0.000245845 | 3.609338184 | TNF,IL6,IL10,IL4 |
| GO:BP | myeloid cell differentiation | GO:0030099 | 0.000246585 | 3.608033215 | ADIPOQ,TNF,VEGFA,IL4,SIRT1,CTNNB1,EP300 |
| GO:BP | negative regulation of transcription, DNA-templated | GO:0045892 | 0.000247961 | 3.605616172 | ADIPOQ,TNF,VEGFA,EDN1,IL4,ESR1,TP53,SIRT1,CTNNB1,EP300,ESR2 |
| GO:BP | carbohydrate metabolic process | GO:0005975 | 0.000251394 | 3.599645906 | ADIPOQ,TNF,EGF,AKT1,TP53,SIRT1,PIK3CA,EP300 |
| GO:BP | negative regulation of nucleic acid-templated transcription | GO:1903507 | 0.000251913 | 3.598749684 | ADIPOQ,TNF,VEGFA,EDN1,IL4,ESR1,TP53,SIRT1,CTNNB1,EP300,ESR2 |
| GO:BP | negative regulation of RNA biosynthetic process | GO:1902679 | 0.000255921 | 3.59189489 | ADIPOQ,TNF,VEGFA,EDN1,IL4,ESR1,TP53,SIRT1,CTNNB1,EP300,ESR2 |
| GO:BP | negative regulation of hydrolase activity | GO:0051346 | 0.000261671 | 3.582243685 | NGF,NOS3,TNF,VEGFA,PTGS2,AKT1 |
| GO:BP | steroid metabolic process | GO:0008202 | 0.000266737 | 3.573916376 | TNF,IL4,ESR1,CAT,SIRT1,SOD1,AGT |
| GO:BP | nervous system process | GO:0050877 | 0.000277805 | 3.556260657 | NGF,NOS3,CXCL12,TNF,MAPK3,IL10,PTGS2,EDN1,AKT1,EP300,SOD1,AGT |
| GO:BP | regulation of heterotypic cell-cell adhesion | GO:0034114 | 0.000278409 | 3.555316925 | ADIPOQ,TNF,IL10 |
| GO:BP | regulation of glucose metabolic process | GO:0010906 | 0.000283113 | 3.548039809 | ADIPOQ,AKT1,TP53,SIRT1,EP300 |
| GO:BP | chronic inflammatory response | GO:0002544 | 0.000283657 | 3.547206702 | TNF,IL10,IL4 |
| GO:BP | organonitrogen compound metabolic process | GO:1901564 | 0.00029928 | 3.52392292 | NGF,ADIPOQ,NOS3,TNF,IL6,VEGFA,MAPK3,IL10,PTGS2,EDN1,EGF,IL4,AKT1,CAT,TP53,SIRT1,CTNNB1,PIK3CA,EP300,MMP3,SOD1,AGT |
| GO:BP | heart morphogenesis | GO:0003007 | 0.000302015 | 3.519971269 | NOS3,VEGFA,EDN1,TP53,CTNNB1,GJA1 |
| GO:BP | regulation of T cell activation | GO:0050863 | 0.000307037 | 3.512809637 | IL6,IL10,IL4,AKT1,CTNNB1,PIK3CA,SOD1 |
| GO:BP | positive regulation of catabolic process | GO:0009896 | 0.000309574 | 3.509236195 | TNF,IL6,MAPK3,EGF,IL4,AKT1,SIRT1 |
| GO:BP | regulation of I-kappaB kinase/NF-kappaB signaling | GO:0043122 | 0.000324018 | 3.489430646 | ADIPOQ,TNF,ESR1,SIRT1,CTNNB1,GJA1 |
| GO:BP | regulation of lipid metabolic process | GO:0019216 | 0.000343167 | 3.464494005 | ADIPOQ,TNF,PTGS2,AKT1,SIRT1,SOD1,AGT |
| GO:BP | female gonad development | GO:0008585 | 0.000354989 | 3.449785218 | NOS3,VEGFA,ESR1,SIRT1,SOD1 |
| GO:BP | reproductive process | GO:0022414 | 0.000358226 | 3.445842729 | NOS3,VEGFA,IL10,PTGS2,EDN1,ESR1,AKT1,SIRT1,CTNNB1,GJA1,SOD1,AGT |
| GO:BP | reproduction | GO:0000003 | 0.000366007 | 3.436510292 | NOS3,VEGFA,IL10,PTGS2,EDN1,ESR1,AKT1,SIRT1,CTNNB1,GJA1,SOD1,AGT |
| GO:BP | neuron projection development | GO:0031175 | 0.000387561 | 3.411660441 | NGF,CXCL12,IL6,VEGFA,EDN1,AKT1,CTNNB1,EP300,SOD1,AGT |
| GO:BP | negative regulation of blood pressure | GO:0045776 | 0.000396691 | 3.401547874 | ADIPOQ,NOS3,TNF |
| GO:BP | superoxide anion generation | GO:0042554 | 0.000396927 | 3.401289132 | EDN1,CYBB,SOD1,AGT |
| GO:BP | response to metal ion | GO:0010038 | 0.000412354 | 3.384730293 | MAPK3,PTGS2,EDN1,AKT1,CAT,CYBB,SOD1 |
| GO:BP | response to wounding | GO:0009611 | 0.000416889 | 3.379979935 | NOS3,TNF,IL6,VEGFA,IL10,EDN1 |
| GO:BP | cellular localization | GO:0051641 | 0.000421551 | 3.375149615 | ADIPOQ,ALB,TNF,VEGFA,MAPK3,IL10,PTGS2,EGF,IL4,ESR1,AKT1,TP53,CTNNB1,GJA1 |
| GO:BP | muscle contraction | GO:0006936 | 0.000428726 | 3.367820165 | TNF,PTGS2,EDN1,PIK3CA,GJA1,SOD1,AGT |
| GO:BP | development of primary female sexual characteristics | GO:0046545 | 0.000428786 | 3.367759167 | NOS3,VEGFA,ESR1,SIRT1,SOD1 |
| GO:BP | response to inactivity | GO:0014854 | 0.00043551 | 3.361002282 | IL10,CAT,PIK3CA |
| GO:BP | regulation of glucose transmembrane transport | GO:0010827 | 0.000444615 | 3.352015688 | ADIPOQ,TNF,EDN1,AKT1 |
| GO:BP | negative regulation of cell migration | GO:0030336 | 0.000444926 | 3.351712404 | ADIPOQ,CXCL12,TNF,IL4,AKT1,GJA1 |
| GO:BP | immune response | GO:0006955 | 0.000458543 | 3.338620017 | CXCL12,TNF,IL6,IL10,EDN1,IL4,ESR1,TP53,SIRT1,PIK3CA,EP300,CYBB |
| GO:BP | negative regulation of cysteine-type endopeptidase activity involved in apoptotic process | GO:0043154 | 0.000469449 | 3.328411402 | TNF,VEGFA,PTGS2,AKT1 |
| GO:BP | positive regulation of cell-cell adhesion | GO:0022409 | 0.000470315 | 3.327610759 | TNF,IL6,IL10,IL4,AKT1,PIK3CA |
| GO:BP | macromolecule localization | GO:0033036 | 0.000476288 | 3.322130421 | ADIPOQ,TNF,IL6,VEGFA,IL10,PTGS2,EDN1,EGF,ESR1,AKT1,TP53,SIRT1,CTNNB1,GJA1 |
| GO:BP | regulation of hydrolase activity | GO:0051336 | 0.000483432 | 3.315664996 | NGF,NOS3,TNF,VEGFA,PTGS2,ESR1,AKT1,SIRT1,SOD1,AGT |
| GO:BP | mammary gland development | GO:0030879 | 0.000486441 | 3.312970028 | VEGFA,EGF,ESR1,AKT1,GJA1 |
| GO:BP | positive regulation of acute inflammatory response | GO:0002675 | 0.000491106 | 3.308824797 | TNF,IL6,PTGS2 |
| GO:BP | cellular protein metabolic process | GO:0044267 | 0.00050671 | 3.295240283 | NGF,ADIPOQ,TNF,IL6,VEGFA,MAPK3,IL10,PTGS2,EDN1,EGF,IL4,AKT1,TP53,SIRT1,CTNNB1,PIK3CA,EP300 |
| GO:BP | female gamete generation | GO:0007292 | 0.000516323 | 3.287078295 | NOS3,PTGS2,EDN1,SIRT1,CTNNB1 |
| GO:BP | regulation of epithelial cell apoptotic process | GO:1904035 | 0.000518708 | 3.28507696 | TNF,IL6,IL10,IL4 |
| GO:BP | negative regulation of gene silencing by miRNA | GO:0060965 | 0.000522909 | 3.281574156 | TNF,ESR1,TP53 |
| GO:BP | cellular response to light stimulus | GO:0071482 | 0.000523217 | 3.281318503 | PTGS2,TP53,SIRT1,EP300,MMP3 |
| GO:BP | regulation of transcription by RNA polymerase II | GO:0006357 | 0.000536317 | 3.270578225 | TNF,IL6,VEGFA,MAPK3,IL10,EDN1,IL4,ESR1,AKT1,TP53,SIRT1,CTNNB1,EP300,ESR2 |
| GO:BP | glial cell differentiation | GO:0010001 | 0.000538745 | 3.268616813 | TNF,IL6,MAPK3,AKT1,CTNNB1,SOD1 |
| GO:BP | regulation of production of molecular mediator of immune response | GO:0002700 | 0.000564468 | 3.248360346 | TNF,IL6,IL10,IL4,SIRT1 |
| GO:BP | regulation of histone modification | GO:0031056 | 0.000567954 | 3.245686556 | VEGFA,MAPK3,TP53,SIRT1,CTNNB1 |
| GO:BP | gene expression | GO:0010467 | 0.000568434 | 3.245319809 | NGF,ADIPOQ,NOS3,TNF,IL6,VEGFA,MAPK3,IL10,PTGS2,EDN1,EGF,IL4,ESR1,AKT1,CAT,TP53,SIRT1,CTNNB1,PIK3CA,GJA1,EP300,CYBB,ESR2,SOD1,AGT |
| GO:BP | maintenance of location | GO:0051235 | 0.000582042 | 3.235045357 | ALB,TNF,IL6,IL10,AKT1,SIRT1 |
| GO:BP | muscle cell differentiation | GO:0042692 | 0.000596047 | 3.224719673 | VEGFA,EDN1,IL4,AKT1,SIRT1,CTNNB1,AGT |
| GO:BP | leukocyte adhesion to vascular endothelial cell | GO:0061756 | 0.000608475 | 3.215757162 | CXCL12,TNF,IL6 |
| GO:BP | liver regeneration | GO:0097421 | 0.000617224 | 3.209557297 | TNF,IL6,IL10 |
| GO:BP | regulation of MHC class II biosynthetic process | GO:0045346 | 0.000620666 | 3.207141993 | IL10,IL4,SIRT1 |
| GO:BP | response to acid chemical | GO:0001101 | 0.000622788 | 3.205660002 | TNF,VEGFA,EDN1,PIK3CA,CYBB |
| GO:BP | negative regulation of cell motility | GO:2000146 | 0.00062628 | 3.203231619 | ADIPOQ,CXCL12,TNF,IL4,AKT1,GJA1 |
| GO:BP | negative regulation of gene silencing by RNA | GO:0060967 | 0.000634621 | 3.19748571 | TNF,ESR1,TP53 |
| GO:BP | negative regulation of posttranscriptional gene silencing | GO:0060149 | 0.000634621 | 3.19748571 | TNF,ESR1,TP53 |
| GO:BP | macroautophagy | GO:0016236 | 0.000636288 | 3.196346177 | MAPK3,IL4,AKT1,TP53,SIRT1,PIK3CA |
| GO:BP | gonad development | GO:0008406 | 0.00064761 | 3.188686634 | NOS3,VEGFA,ESR1,SIRT1,GJA1,SOD1 |
| GO:BP | mammary gland alveolus development | GO:0060749 | 0.000691967 | 3.159914758 | VEGFA,EGF,ESR1 |
| GO:BP | mammary gland lobule development | GO:0061377 | 0.000691967 | 3.159914758 | VEGFA,EGF,ESR1 |
| GO:BP | developmental cell growth | GO:0048588 | 0.00069805 | 3.156113504 | NGF,CXCL12,VEGFA,EDN1,CTNNB1,AGT |
| GO:BP | regulation of secretion by cell | GO:1903530 | 0.000698449 | 3.155865037 | ADIPOQ,CXCL12,TNF,IL6,EDN1,IL4,GJA1,AGT |
| GO:BP | negative regulation of cysteine-type endopeptidase activity | GO:2000117 | 0.000707161 | 3.150481967 | TNF,VEGFA,PTGS2,AKT1 |
| GO:BP | development of primary sexual characteristics | GO:0045137 | 0.000735944 | 3.133155042 | NOS3,VEGFA,ESR1,SIRT1,GJA1,SOD1 |
| GO:BP | negative regulation of cellular component movement | GO:0051271 | 0.000737503 | 3.132236204 | ADIPOQ,CXCL12,TNF,IL4,AKT1,GJA1 |
| GO:BP | positive regulation of DNA binding | GO:0043388 | 0.000737936 | 3.131981316 | NGF,EGF,CTNNB1,EP300 |
| GO:BP | MHC class II biosynthetic process | GO:0045342 | 0.000753236 | 3.12306904 | IL10,IL4,SIRT1 |
| GO:BP | extrinsic apoptotic signaling pathway | GO:0097191 | 0.000755523 | 3.12175249 | NGF,NOS3,TNF,IL4,AKT1,AGT |
| GO:BP | vasodilation | GO:0042311 | 0.00076974 | 3.113655762 | NOS3,TNF,SOD1,AGT |
| GO:BP | protein localization to cell surface | GO:0034394 | 0.000783426 | 3.106002198 | TNF,EGF,AKT1,CTNNB1 |
| GO:BP | lymphocyte proliferation | GO:0046651 | 0.000798919 | 3.097497303 | IL6,IL10,IL4,TP53,CTNNB1,GJA1 |
| GO:BP | response to ethanol | GO:0045471 | 0.000820334 | 3.08600954 | ADIPOQ,TNF,CAT,CYBB,SOD1 |
| GO:BP | female sex differentiation | GO:0046660 | 0.000820334 | 3.08600954 | NOS3,VEGFA,ESR1,SIRT1,SOD1 |
| GO:BP | lymphocyte differentiation | GO:0030098 | 0.00082336 | 3.084410098 | IL6,IL10,IL4,TP53,CTNNB1,EP300,SOD1 |
| GO:BP | cellular macromolecule biosynthetic process | GO:0034645 | 0.000831071 | 3.080362067 | ADIPOQ,TNF,IL6,VEGFA,MAPK3,EGF,AKT1,TP53,SIRT1,CTNNB1 |
| GO:BP | regulation of protein localization | GO:0032880 | 0.000831991 | 3.079881514 | ADIPOQ,TNF,IL6,VEGFA,PTGS2,EGF,AKT1,CTNNB1 |
| GO:BP | cellular macromolecule metabolic process | GO:0044260 | 0.000844146 | 3.07358228 | NGF,ADIPOQ,TNF,IL6,VEGFA,MAPK3,IL10,PTGS2,EDN1,EGF,IL4,AKT1,CAT,TP53,SIRT1,CTNNB1,PIK3CA |
| GO:BP | B cell differentiation | GO:0030183 | 0.000846362 | 3.072443646 | IL6,IL10,IL4,TP53,EP300 |
| GO:BP | macrophage activation | GO:0042116 | 0.000863431 | 3.063772319 | TNF,IL6,IL10,IL4 |
| GO:BP | mononuclear cell proliferation | GO:0032943 | 0.000864478 | 3.063245932 | IL6,IL10,IL4,TP53,CTNNB1,GJA1 |
| GO:BP | regulation of cellular carbohydrate metabolic process | GO:0010675 | 0.000875329 | 3.05782882 | ADIPOQ,AKT1,TP53,SIRT1,EP300 |
| GO:BP | cell adhesion | GO:0007155 | 0.000888018 | 3.051578104 | ADIPOQ,CXCL12,TNF,IL6,VEGFA,IL10,IL4,AKT1,CTNNB1,PIK3CA |
| GO:BP | regulation of adaptive immune response | GO:0002819 | 0.000888696 | 3.051246956 | TNF,IL6,IL10,IL4,SIRT1 |
| GO:BP | transcription by RNA polymerase II | GO:0006366 | 0.00089157 | 3.049844432 | TNF,IL6,VEGFA,MAPK3,IL10,EDN1,IL4,ESR1,AKT1,TP53,SIRT1,CTNNB1,EP300,ESR2 |
| GO:BP | biological adhesion | GO:0022610 | 0.000927772 | 3.03255852 | ADIPOQ,CXCL12,TNF,IL6,VEGFA,IL10,IL4,AKT1,CTNNB1,PIK3CA |
| GO:BP | immunoglobulin production | GO:0002377 | 0.00093427 | 3.029527517 | TNF,IL6,IL10,IL4 |
| GO:BP | positive regulation of DNA biosynthetic process | GO:2000573 | 0.000935454 | 3.028977572 | TNF,VEGFA,MAPK3,CTNNB1 |
| GO:BP | negative regulation of cell development | GO:0010721 | 0.00094304 | 3.025469967 | TNF,IL6,VEGFA,TP53,CTNNB1 |
| GO:BP | small molecule biosynthetic process | GO:0044283 | 0.00098243 | 3.007698567 | ADIPOQ,TNF,PTGS2,EDN1,SIRT1,EP300,SOD1,MPO |
| GO:BP | negative regulation of secretion | GO:0051048 | 0.001014358 | 2.993808658 | ADIPOQ,TNF,EDN1,EGF,GJA1 |
| GO:BP | regulation of hormone secretion | GO:0046883 | 0.001025732 | 2.988966035 | ADIPOQ,TNF,IL6,EDN1,GJA1,AGT |
| GO:BP | regulation of protein kinase B signaling | GO:0051896 | 0.001034441 | 2.985294329 | TNF,VEGFA,EGF,SIRT1,PIK3CA |
| GO:BP | energy derivation by oxidation of organic compounds | GO:0015980 | 0.001036865 | 2.984277838 | TNF,IL4,AKT1,CAT,TP53,PIK3CA |
| GO:BP | positive regulation of MAP kinase activity | GO:0043406 | 0.001043116 | 2.981667583 | TNF,VEGFA,EDN1,EGF |
| GO:BP | regulation of receptor-mediated endocytosis | GO:0048259 | 0.001048534 | 2.979417576 | ADIPOQ,VEGFA,EGF,IL4 |
| GO:BP | regulation of vesicle-mediated transport | GO:0060627 | 0.001088269 | 2.963263625 | ADIPOQ,TNF,VEGFA,MAPK3,EGF,IL4 |
| GO:BP | regulation of chronic inflammatory response to antigenic stimulus | GO:0002874 | 0.001090056 | 2.962551022 | TNF,IL10 |
| GO:BP | chronic inflammatory response to antigenic stimulus | GO:0002439 | 0.001090056 | 2.962551022 | TNF,IL10 |
| GO:BP | regulation of metal ion transport | GO:0010959 | 0.001099113 | 2.958957536 | NOS3,CXCL12,PTGS2,EGF,AKT1,CTNNB1,AGT |
| GO:BP | biological regulation | GO:0065007 | 0.001126541 | 2.948252987 | NGF,ADIPOQ,NOS3,CXCL12,ALB,TNF,IL6,VEGFA,MAPK3,IL10,PTGS2,EDN1,EGF,IL4,ESR1,AKT1,CAT,TP53,SIRT1,CTNNB1,PIK3CA,GJA1,EP300,CYBB,MMP3,ESR2,SOD1,AGT,MPO |
| GO:BP | negative regulation of endopeptidase activity | GO:0010951 | 0.001131696 | 2.94627035 | NGF,TNF,VEGFA,PTGS2,AKT1,AGT |
| GO:BP | embryonic morphogenesis | GO:0048598 | 0.001137261 | 2.944139763 | ADIPOQ,MAPK3,IL10,EDN1,TP53,CTNNB1,GJA1,SOD1 |
| GO:BP | embryo implantation | GO:0007566 | 0.001164577 | 2.933831629 | VEGFA,PTGS2,GJA1,SOD1 |
| GO:BP | negative regulation of protein binding | GO:0032091 | 0.001167631 | 2.932694434 | ADIPOQ,MAPK3,IL10,AKT1 |
| GO:BP | negative regulation of cytokine production | GO:0001818 | 0.00119719 | 2.921836777 | ADIPOQ,TNF,IL6,IL10,IL4 |
| GO:BP | positive regulation of apoptotic signaling pathway | GO:2001235 | 0.001211553 | 2.916657638 | TNF,TP53,SIRT1,SOD1,AGT |
| GO:BP | regulation of leukocyte activation | GO:0002694 | 0.001224729 | 2.911960079 | TNF,IL6,IL10,IL4,AKT1,CTNNB1,PIK3CA,SOD1 |
| GO:BP | negative regulation of locomotion | GO:0040013 | 0.001239302 | 2.906822816 | ADIPOQ,CXCL12,TNF,IL4,AKT1,GJA1 |
| GO:BP | production of molecular mediator of immune response | GO:0002440 | 0.001248042 | 2.903770722 | TNF,IL6,IL10,IL4,SIRT1 |
| GO:BP | epithelial cell apoptotic process | GO:1904019 | 0.001307925 | 2.883417258 | TNF,IL6,IL10,IL4 |
| GO:BP | negative regulation of extrinsic apoptotic signaling pathway | GO:2001237 | 0.001324841 | 2.877836171 | NOS3,TNF,IL4,AKT1 |
| GO:BP | vascular endothelial growth factor production | GO:0010573 | 0.001344807 | 2.871340065 | TNF,IL6,PTGS2 |
| GO:BP | acute inflammatory response | GO:0002526 | 0.001355405 | 2.867931016 | TNF,IL6,PTGS2,IL4 |
| GO:BP | negative regulation of peptidase activity | GO:0010466 | 0.001370651 | 2.863073181 | NGF,TNF,VEGFA,PTGS2,AKT1,AGT |
| GO:BP | regulation of calcium ion transport | GO:0051924 | 0.001403227 | 2.852871952 | NOS3,CXCL12,PTGS2,EGF,CTNNB1,AGT |
| GO:BP | regulation of blood circulation | GO:1903522 | 0.001436436 | 2.842713622 | NOS3,TNF,PTGS2,EDN1,GJA1,AGT |
| GO:BP | regulation of cysteine-type endopeptidase activity involved in apoptotic process | GO:0043281 | 0.001448507 | 2.839079303 | TNF,VEGFA,PTGS2,AKT1,SIRT1 |
| GO:BP | multicellular organismal reproductive process | GO:0048609 | 0.001455411 | 2.837014476 | NOS3,PTGS2,EDN1,ESR1,AKT1,SIRT1,CTNNB1,GJA1,SOD1 |
| GO:BP | cellular response to hydrogen peroxide | GO:0070301 | 0.001492494 | 2.826087456 | IL6,IL10,EDN1,SIRT1 |
| GO:BP | neuron development | GO:0048666 | 0.00150273 | 2.823119117 | NGF,CXCL12,IL6,VEGFA,EDN1,AKT1,CTNNB1,EP300,SOD1,AGT |
| GO:BP | positive regulation of vasculature development | GO:1904018 | 0.001519693 | 2.818243995 | NOS3,VEGFA,IL10,SIRT1,CYBB |
| GO:BP | positive regulation of angiogenesis | GO:0045766 | 0.001519693 | 2.818243995 | NOS3,VEGFA,IL10,SIRT1,CYBB |
| GO:BP | regulation of endopeptidase activity | GO:0052548 | 0.001560358 | 2.806775842 | NGF,TNF,VEGFA,PTGS2,AKT1,SIRT1,AGT |
| GO:BP | positive regulation of oxidoreductase activity | GO:0051353 | 0.001572072 | 2.803527513 | TNF,EDN1,AKT1,AGT |
| GO:BP | positive regulation of interleukin-8 production | GO:0032757 | 0.001587914 | 2.799173131 | ADIPOQ,TNF,IL6 |
| GO:BP | regulation of nervous system process | GO:0031644 | 0.001595859 | 2.79700541 | NOS3,TNF,IL10,EDN1 |
| GO:BP | regulation of tumor necrosis factor production | GO:0032680 | 0.001617203 | 2.791235592 | ADIPOQ,IL6,IL10,IL4,CYBB |
| GO:BP | tumor necrosis factor production | GO:0032640 | 0.001617203 | 2.791235592 | ADIPOQ,IL6,IL10,IL4,CYBB |
| GO:BP | proteolysis | GO:0006508 | 0.00168318 | 2.773869495 | NGF,TNF,VEGFA,IL10,PTGS2,EGF,AKT1,TP53,SIRT1,CTNNB1,MMP3,AGT |
| GO:BP | regulation of leukocyte apoptotic process | GO:2000106 | 0.001721772 | 2.764024415 | CXCL12,IL10,TP53,SIRT1 |
| GO:BP | positive regulation of growth | GO:0045927 | 0.001729223 | 2.762148895 | NGF,CXCL12,VEGFA,EDN1,AKT1 |
| GO:BP | multicellular organism reproduction | GO:0032504 | 0.001759232 | 2.754676981 | NOS3,PTGS2,EDN1,ESR1,AKT1,SIRT1,CTNNB1,GJA1,SOD1 |
| GO:BP | calcium ion transport | GO:0006816 | 0.001802766 | 2.744060647 | NOS3,CXCL12,PTGS2,EDN1,EGF,CTNNB1,AGT |
| GO:BP | regulation of fatty acid metabolic process | GO:0019217 | 0.001803717 | 2.743831517 | ADIPOQ,PTGS2,AKT1,SIRT1 |
| GO:BP | activation of protein kinase activity | GO:0032147 | 0.001822839 | 2.739251576 | VEGFA,EGF,IL4,PIK3CA,AGT |
| GO:BP | epithelial cell differentiation | GO:0030855 | 0.00188054 | 2.725717411 | ADIPOQ,TNF,VEGFA,ESR1,AKT1,CTNNB1,GJA1,SOD1 |
| GO:BP | regulation of tumor necrosis factor superfamily cytokine production | GO:1903555 | 0.001882701 | 2.725218568 | ADIPOQ,IL6,IL10,IL4,CYBB |
| GO:BP | tumor necrosis factor superfamily cytokine production | GO:0071706 | 0.001882701 | 2.725218568 | ADIPOQ,IL6,IL10,IL4,CYBB |
| GO:BP | regulation of reactive oxygen species metabolic process | GO:2000377 | 0.001888625 | 2.723854172 | TNF,TP53,MMP3,SOD1,AGT |
| GO:BP | ovulation | GO:0030728 | 0.001955028 | 2.708846982 | NOS3,PTGS2,SIRT1 |
| GO:BP | glucose transmembrane transport | GO:1904659 | 0.002038853 | 2.690614061 | ADIPOQ,TNF,EDN1,AKT1 |
| GO:BP | cellular protein localization | GO:0034613 | 0.002073463 | 2.683303802 | ADIPOQ,TNF,VEGFA,IL10,PTGS2,EGF,ESR1,AKT1,TP53,CTNNB1,GJA1 |
| GO:BP | gastrulation | GO:0007369 | 0.002078926 | 2.682160953 | ADIPOQ,IL10,TP53,CTNNB1,GJA1 |
| GO:BP | kidney epithelium development | GO:0072073 | 0.002097298 | 2.678339862 | ADIPOQ,VEGFA,CAT,CTNNB1,AGT |
| GO:BP | extrinsic apoptotic signaling pathway via death domain receptors | GO:0008625 | 0.002099435 | 2.677897638 | NGF,NOS3,TNF |
| GO:BP | cellular macromolecule localization | GO:0070727 | 0.002171057 | 2.663328766 | ADIPOQ,TNF,VEGFA,IL10,PTGS2,EGF,ESR1,AKT1,TP53,CTNNB1,GJA1 |
| GO:BP | positive regulation of chemokine production | GO:0032722 | 0.002186685 | 2.660213731 | ADIPOQ,TNF,IL6 |
| GO:BP | regeneration | GO:0031099 | 0.002224687 | 2.652731091 | CXCL12,TNF,IL6,IL10 |
| GO:BP | granulocyte chemotaxis | GO:0071621 | 0.002303451 | 2.637620986 | VEGFA,MAPK3,EDN1,IL4 |
| GO:BP | hexose transmembrane transport | GO:0008645 | 0.002358568 | 2.627351662 | ADIPOQ,TNF,EDN1,AKT1 |
| GO:BP | regulation of peptidase activity | GO:0052547 | 0.002383399 | 2.622803267 | NGF,TNF,VEGFA,PTGS2,AKT1,SIRT1,AGT |
| GO:BP | regulation of leukocyte differentiation | GO:1902105 | 0.002396545 | 2.620414375 | ADIPOQ,TNF,IL10,IL4,CTNNB1,SOD1 |
| GO:BP | negative regulation of autophagy | GO:0010507 | 0.002403752 | 2.619110401 | IL10,AKT1,TP53,PIK3CA |
| GO:BP | macromolecule biosynthetic process | GO:0009059 | 0.002449471 | 2.610927772 | ADIPOQ,TNF,IL6,VEGFA,MAPK3,IL10,EDN1,EGF,IL4,ESR1,AKT1,CAT,TP53,SIRT1,CTNNB1 |
| GO:BP | regulation of response to wounding | GO:1903034 | 0.002459217 | 2.609203135 | NOS3,TNF,IL10,EDN1 |
| GO:BP | plasma membrane bounded cell projection organization | GO:0120036 | 0.002515732 | 2.599335597 | NGF,CXCL12,IL6,VEGFA,EDN1,AKT1,CTNNB1,PIK3CA,EP300,SOD1,AGT |
| GO:BP | regulation of muscle cell apoptotic process | GO:0010660 | 0.002533307 | 2.596312169 | EDN1,TP53,SIRT1,AGT |
| GO:BP | defense response to other organism | GO:0098542 | 0.002573245 | 2.58951883 | TNF,IL6,MAPK3,IL10,EDN1,IL4,TP53,EP300,CYBB,MPO |
| GO:BP | monosaccharide transmembrane transport | GO:0015749 | 0.002621748 | 2.581409048 | ADIPOQ,TNF,EDN1,AKT1 |
| GO:BP | regulation of cysteine-type endopeptidase activity | GO:2000116 | 0.002630416 | 2.579975489 | TNF,VEGFA,PTGS2,AKT1,SIRT1 |
| GO:BP | positive regulation of lipid metabolic process | GO:0045834 | 0.002655247 | 2.575895067 | ADIPOQ,TNF,PTGS2,AKT1,AGT |
| GO:BP | regulation of neural precursor cell proliferation | GO:2000177 | 0.002692081 | 2.56991182 | VEGFA,EGF,TP53,CTNNB1 |
| GO:BP | carbohydrate transmembrane transport | GO:0034219 | 0.002808929 | 2.551459256 | ADIPOQ,TNF,EDN1,AKT1 |
| GO:BP | regulation of animal organ morphogenesis | GO:2000027 | 0.002834402 | 2.547538574 | TNF,VEGFA,EDN1,CTNNB1,AGT |
| GO:BP | positive regulation of defense response | GO:0031349 | 0.002835618 | 2.547352272 | TNF,IL6,MAPK3,PTGS2,EP300,AGT |
| GO:BP | gliogenesis | GO:0042063 | 0.002872268 | 2.54177505 | TNF,IL6,MAPK3,AKT1,CTNNB1,SOD1 |
| GO:BP | regulation of extrinsic apoptotic signaling pathway | GO:2001236 | 0.002927482 | 2.533505792 | NOS3,TNF,IL4,AKT1,AGT |
| GO:BP | negative regulation of smooth muscle cell proliferation | GO:0048662 | 0.003018741 | 2.52017408 | ADIPOQ,NOS3,IL10 |
| GO:BP | positive regulation of immune effector process | GO:0002699 | 0.003051881 | 2.515432447 | TNF,IL6,IL10,IL4,SIRT1 |
| GO:BP | response to muscle stretch | GO:0035994 | 0.003060371 | 2.514225853 | EDN1,CTNNB1,PIK3CA |
| GO:BP | hormone secretion | GO:0046879 | 0.003140913 | 2.50294409 | ADIPOQ,TNF,IL6,EDN1,GJA1,AGT |
| GO:BP | signal release | GO:0023061 | 0.003205225 | 2.494141454 | ADIPOQ,CXCL12,TNF,IL6,EDN1,GJA1,AGT |
| GO:BP | positive regulation of oxidative stress-induced cell death | GO:1903209 | 0.003221578 | 2.491931399 | TNF,MMP3,SOD1 |
| GO:BP | muscle cell apoptotic process | GO:0010657 | 0.003247875 | 2.488400651 | EDN1,TP53,SIRT1,AGT |
| GO:BP | regulation of cell cycle process | GO:0010564 | 0.003271195 | 2.485293512 | TNF,EDN1,EGF,AKT1,TP53,SIRT1,CTNNB1 |
| GO:BP | response to transition metal nanoparticle | GO:1990267 | 0.003279075 | 2.484248587 | TNF,MPO |
| GO:BP | response to gold nanoparticle | GO:1990268 | 0.003279075 | 2.484248587 | TNF,MPO |
| GO:BP | negative regulation of intrinsic apoptotic signaling pathway | GO:2001243 | 0.003282155 | 2.483840862 | CXCL12,PTGS2,AKT1,SIRT1 |
| GO:BP | cell projection organization | GO:0030030 | 0.003315463 | 2.479455842 | NGF,CXCL12,IL6,VEGFA,EDN1,AKT1,CTNNB1,PIK3CA,EP300,SOD1,AGT |
| GO:BP | organ growth | GO:0035265 | 0.003324184 | 2.478314974 | EDN1,ESR1,AKT1,SOD1,AGT |
| GO:BP | regulation of signaling receptor activity | GO:0010469 | 0.003332231 | 2.477264919 | TNF,IL10,EDN1,EGF,ESR2 |
| GO:BP | cellular response to peptide hormone stimulus | GO:0071375 | 0.003336643 | 2.476690256 | ADIPOQ,EDN1,AKT1,SIRT1,PIK3CA,AGT |
| GO:BP | regulation of lipid biosynthetic process | GO:0046890 | 0.003405158 | 2.46786274 | TNF,PTGS2,AKT1,SIRT1,SOD1 |
| GO:BP | sprouting angiogenesis | GO:0002040 | 0.003430306 | 2.464667115 | VEGFA,IL10,PTGS2,AKT1 |
| GO:BP | type 2 immune response | GO:0042092 | 0.003490123 | 2.457159261 | IL6,IL10,IL4 |
| GO:BP | regulation of ATP metabolic process | GO:1903578 | 0.003534843 | 2.451629897 | TNF,IL4,TP53,EP300 |
| GO:BP | regulation of protein binding | GO:0043393 | 0.003592407 | 2.444614475 | ADIPOQ,MAPK3,IL10,AKT1,EP300 |
| GO:BP | acute-phase response | GO:0006953 | 0.003676895 | 2.434518784 | TNF,IL6,PTGS2 |
| GO:BP | wound healing | GO:0042060 | 0.003686743 | 2.433357185 | NOS3,TNF,IL6,VEGFA,EDN1 |
| GO:BP | hormone transport | GO:0009914 | 0.003758078 | 2.425034201 | ADIPOQ,TNF,IL6,EDN1,GJA1,AGT |
| GO:BP | response to fluid shear stress | GO:0034405 | 0.003806307 | 2.419496187 | NOS3,PTGS2,AKT1 |
| GO:BP | regulation of cellular response to growth factor stimulus | GO:0090287 | 0.003832369 | 2.416532732 | IL4,TP53,SIRT1,CTNNB1,EP300,AGT |
| GO:BP | ion transport | GO:0006811 | 0.003842513 | 2.415384694 | NOS3,CXCL12,TNF,PTGS2,EDN1,EGF,AKT1,CTNNB1,GJA1,CYBB,AGT |
| GO:BP | positive regulation of immune response | GO:0050778 | 0.003866242 | 2.412710965 | TNF,IL6,IL10,IL4,SIRT1,PIK3CA,EP300 |
| GO:BP | regulation of body fluid levels | GO:0050878 | 0.003879972 | 2.411171418 | NOS3,IL6,VEGFA,EDN1,PIK3CA,GJA1 |
| GO:BP | cellular response to radiation | GO:0071478 | 0.003892138 | 2.409811754 | PTGS2,TP53,SIRT1,EP300,MMP3 |
| GO:BP | monocarboxylic acid metabolic process | GO:0032787 | 0.00391001 | 2.407822164 | ADIPOQ,PTGS2,EDN1,AKT1,TP53,SIRT1,EP300 |
| GO:BP | stem cell differentiation | GO:0048863 | 0.003948764 | 2.403538864 | MAPK3,EDN1,ESR1,TP53,CTNNB1 |
| GO:BP | positive regulation of leukocyte activation | GO:0002696 | 0.004004108 | 2.39749419 | TNF,IL6,IL10,IL4,AKT1,PIK3CA |
| GO:BP | positive regulation of leukocyte apoptotic process | GO:2000108 | 0.004019901 | 2.395784589 | IL10,TP53,SIRT1 |
| GO:BP | removal of superoxide radicals | GO:0019430 | 0.004019901 | 2.395784589 | NOS3,SOD1,MPO |
| GO:BP | positive regulation of transcription from RNA polymerase II promoter involved in cellular response to chemical stimulus | GO:1901522 | 0.004069025 | 2.390509667 | VEGFA,TP53,EP300 |
| GO:BP | regulation of organic acid transport | GO:0032890 | 0.004102177 | 2.38698556 | TNF,EDN1,AKT1,AGT |
| GO:BP | cellular response to acid chemical | GO:0071229 | 0.004228921 | 2.373770426 | TNF,VEGFA,PIK3CA,CYBB |
| GO:BP | carbohydrate biosynthetic process | GO:0016051 | 0.004263782 | 2.370205018 | ADIPOQ,EGF,AKT1,SIRT1,EP300 |
| GO:BP | response to ketone | GO:1901654 | 0.004283565 | 2.368194689 | EDN1,AKT1,SIRT1,PIK3CA,CYBB |
| GO:BP | chemical synaptic transmission | GO:0007268 | 0.004326873 | 2.363825827 | NGF,ADIPOQ,TNF,PTGS2,EDN1,AKT1,CTNNB1 |
| GO:BP | anterograde trans-synaptic signaling | GO:0098916 | 0.004326873 | 2.363825827 | NGF,ADIPOQ,TNF,PTGS2,EDN1,AKT1,CTNNB1 |
| GO:BP | heterotypic cell-cell adhesion | GO:0034113 | 0.004432791 | 2.353322757 | ADIPOQ,TNF,IL10 |
| GO:BP | positive regulation of adaptive immune response | GO:0002821 | 0.004451739 | 2.351470274 | TNF,IL6,IL4,SIRT1 |
| GO:BP | prostanoid biosynthetic process | GO:0046457 | 0.004464008 | 2.350275029 | PTGS2,EDN1,SIRT1 |
| GO:BP | prostaglandin biosynthetic process | GO:0001516 | 0.004464008 | 2.350275029 | PTGS2,EDN1,SIRT1 |
| GO:BP | positive regulation of signaling receptor activity | GO:2000273 | 0.004606895 | 2.336591671 | IL10,EDN1,EGF |
| GO:BP | regulation of cell size | GO:0008361 | 0.004645326 | 2.332983808 | NGF,CXCL12,VEGFA,EDN1 |
| GO:BP | trans-synaptic signaling | GO:0099537 | 0.004663873 | 2.331253251 | NGF,ADIPOQ,TNF,PTGS2,EDN1,AKT1,CTNNB1 |
| GO:BP | positive regulation of cell activation | GO:0050867 | 0.004666135 | 2.331042745 | TNF,IL6,IL10,IL4,AKT1,PIK3CA |
| GO:BP | modulation of chemical synaptic transmission | GO:0050804 | 0.004696522 | 2.328223624 | NGF,ADIPOQ,TNF,PTGS2,EDN1 |
| GO:BP | regulation of oxidative stress-induced neuron death | GO:1903203 | 0.004724912 | 2.325606321 | TNF,IL10,CTNNB1 |
| GO:BP | positive regulation of axon extension involved in axon guidance | GO:0048842 | 0.00474449 | 2.323810426 | CXCL12,VEGFA |
| GO:BP | regulation of trans-synaptic signaling | GO:0099177 | 0.004749543 | 2.32334817 | NGF,ADIPOQ,TNF,PTGS2,EDN1 |
| GO:BP | circadian rhythm | GO:0007623 | 0.004801051 | 2.318663653 | ADIPOQ,TNF,TP53,SIRT1,EP300 |
| GO:BP | gamete generation | GO:0007276 | 0.004807183 | 2.318109366 | NOS3,PTGS2,EDN1,AKT1,SIRT1,CTNNB1,GJA1,SOD1 |
| GO:BP | negative regulation of leukocyte cell-cell adhesion | GO:1903038 | 0.004827427 | 2.316284321 | CXCL12,IL10,IL4,AKT1 |
| GO:BP | regulation of immune response | GO:0050776 | 0.004871363 | 2.312349471 | TNF,IL6,IL10,IL4,ESR1,SIRT1,PIK3CA,EP300 |
| GO:BP | positive regulation of cell cycle | GO:0045787 | 0.004899818 | 2.309820028 | TNF,IL10,EDN1,EGF,AKT1 |
| GO:BP | cellular response to external stimulus | GO:0071496 | 0.00491253 | 2.308694778 | ALB,MAPK3,PTGS2,TP53,SIRT1,AGT |
| GO:BP | granulocyte migration | GO:0097530 | 0.004915824 | 2.308403637 | VEGFA,MAPK3,EDN1,IL4 |
| GO:BP | positive regulation of mitotic nuclear division | GO:0045840 | 0.004926547 | 2.307457328 | TNF,EDN1,EGF |
| GO:BP | positive regulation of axonogenesis | GO:0050772 | 0.005034143 | 2.298074495 | NGF,CXCL12,VEGFA |
| GO:BP | regulation of tissue remodeling | GO:0034103 | 0.005112828 | 2.291338849 | IL6,TP53,GJA1,AGT |
| GO:BP | positive regulation of cellular component biogenesis | GO:0044089 | 0.005145168 | 2.288600452 | TNF,VEGFA,ESR1,TP53,PIK3CA,MMP3,AGT |
| GO:BP | regulation of leukocyte proliferation | GO:0070663 | 0.005248862 | 2.279934866 | IL6,MAPK3,IL10,IL4,CTNNB1 |
| GO:BP | positive regulation of protein-containing complex assembly | GO:0031334 | 0.005314268 | 2.274556521 | TNF,VEGFA,ESR1,TP53,MMP3 |
| GO:BP | endocrine system development | GO:0035270 | 0.005427388 | 2.265409164 | IL6,MAPK3,EDN1,AKT1 |
| GO:BP | positive regulation of cold-induced thermogenesis | GO:0120162 | 0.00543106 | 2.265115421 | ADIPOQ,VEGFA,IL4,GJA1 |
| GO:BP | cellular response to superoxide | GO:0071451 | 0.005507287 | 2.259062329 | NOS3,SOD1,MPO |
| GO:BP | cellular response to oxygen radical | GO:0071450 | 0.005507287 | 2.259062329 | NOS3,SOD1,MPO |
| GO:BP | regulation of hormone levels | GO:0010817 | 0.005647128 | 2.248172345 | ADIPOQ,TNF,IL6,EDN1,ESR1,GJA1,AGT |
| GO:BP | positive regulation of leukocyte cell-cell adhesion | GO:1903039 | 0.00569551 | 2.244467384 | TNF,IL6,IL4,AKT1,PIK3CA |
| GO:BP | synaptic signaling | GO:0099536 | 0.005809957 | 2.235827049 | NGF,ADIPOQ,TNF,PTGS2,EDN1,AKT1,CTNNB1 |
| GO:BP | regulation of lymphocyte activation | GO:0051249 | 0.005820096 | 2.235069839 | IL6,IL10,IL4,AKT1,CTNNB1,PIK3CA,SOD1 |
| GO:BP | microglial cell activation | GO:0001774 | 0.005854542 | 2.232507039 | TNF,IL6,IL4 |
| GO:BP | glucose homeostasis | GO:0042593 | 0.005932355 | 2.226772877 | ADIPOQ,IL6,AKT1,SIRT1,PIK3CA |
| GO:BP | carbohydrate homeostasis | GO:0033500 | 0.006053648 | 2.217982822 | ADIPOQ,IL6,AKT1,SIRT1,PIK3CA |
| GO:BP | organic substance transport | GO:0071702 | 0.00621233 | 2.206745489 | ADIPOQ,CXCL12,TNF,IL6,IL10,PTGS2,EDN1,EGF,AKT1,TP53,SIRT1,GJA1 |
| GO:BP | oxoacid metabolic process | GO:0043436 | 0.006326269 | 2.198852339 | ADIPOQ,NOS3,PTGS2,EDN1,AKT1,TP53,SIRT1,EP300,MPO |
| GO:BP | negative regulation of intracellular signal transduction | GO:1902532 | 0.006352168 | 2.197078024 | ADIPOQ,CXCL12,PTGS2,ESR1,AKT1,SIRT1,AGT |
| GO:BP | regulation of lymphocyte mediated immunity | GO:0002706 | 0.006445482 | 2.190744573 | TNF,IL6,IL10,IL4 |
| GO:BP | protein localization | GO:0008104 | 0.00648956 | 2.187784734 | ADIPOQ,TNF,IL6,VEGFA,IL10,PTGS2,EGF,ESR1,AKT1,TP53,CTNNB1,GJA1 |
| GO:BP | positive regulation of production of molecular mediator of immune response | GO:0002702 | 0.006532115 | 2.184946155 | IL6,IL10,IL4,SIRT1 |
| GO:BP | myeloid cell apoptotic process | GO:0033028 | 0.006564457 | 2.182801202 | ADIPOQ,IL6,SIRT1 |
| GO:BP | regulation of adaptive immune response based on somatic recombination of immune receptors built from immunoglobulin superfamily domains | GO:0002822 | 0.006600167 | 2.180445058 | TNF,IL6,IL10,IL4 |
| GO:BP | regulation of fibroblast proliferation | GO:0048145 | 0.00662264 | 2.178968834 | ESR1,TP53,CTNNB1,AGT |
| GO:BP | astrocyte differentiation | GO:0048708 | 0.006699034 | 2.173987808 | TNF,IL6,MAPK3 |
| GO:BP | organic substance catabolic process | GO:1901575 | 0.006780675 | 2.168727088 | ADIPOQ,NOS3,TNF,IL6,IL10,EGF,AKT1,TP53,SIRT1,CTNNB1,EP300 |
| GO:BP | fibroblast proliferation | GO:0048144 | 0.006960311 | 2.157371352 | ESR1,TP53,CTNNB1,AGT |
| GO:BP | carboxylic acid metabolic process | GO:0019752 | 0.007035084 | 2.152730733 | ADIPOQ,NOS3,PTGS2,EDN1,AKT1,TP53,SIRT1,EP300 |
| GO:BP | neuron death in response to oxidative stress | GO:0036475 | 0.007037945 | 2.152554159 | TNF,IL10,CTNNB1 |
| GO:BP | branching involved in blood vessel morphogenesis | GO:0001569 | 0.007037945 | 2.152554159 | VEGFA,EDN1,CTNNB1 |
| GO:BP | regulation of myeloid leukocyte differentiation | GO:0002761 | 0.007103127 | 2.148550399 | ADIPOQ,TNF,IL4,CTNNB1 |
| GO:BP | negative regulation of chronic inflammatory response | GO:0002677 | 0.007341266 | 2.134229016 | IL10,IL4 |
| GO:BP | regulation of signal transduction by p53 class mediator | GO:1901796 | 0.00738844 | 2.13144727 | AKT1,TP53,SIRT1,EP300 |
| GO:BP | organic acid metabolic process | GO:0006082 | 0.007682769 | 2.114482215 | ADIPOQ,NOS3,PTGS2,EDN1,AKT1,TP53,SIRT1,EP300,MPO |
| GO:BP | protein deacetylation | GO:0006476 | 0.007684444 | 2.114387538 | VEGFA,TP53,SIRT1,EP300 |
| GO:BP | regulation of neuroblast proliferation | GO:1902692 | 0.007714356 | 2.112700348 | VEGFA,TP53,CTNNB1 |
| GO:BP | regulation of cholesterol efflux | GO:0010874 | 0.007823653 | 2.106590437 | ADIPOQ,EGF,SIRT1 |
| GO:BP | positive regulation of organelle organization | GO:0010638 | 0.007949511 | 2.099659607 | TNF,MAPK3,EDN1,EGF,TP53,CTNNB1 |
| GO:BP | positive regulation of cell projection organization | GO:0031346 | 0.007956222 | 2.099293098 | NGF,CXCL12,VEGFA,PIK3CA,EP300,AGT |
| GO:BP | regulation of systemic arterial blood pressure by endothelin | GO:0003100 | 0.00798564 | 2.097690287 | NOS3,EDN1 |
| GO:BP | regulation of granulocyte chemotaxis | GO:0071622 | 0.008070752 | 2.093085996 | MAPK3,EDN1,IL4 |
| GO:BP | carbohydrate transport | GO:0008643 | 0.008179712 | 2.087261995 | ADIPOQ,TNF,EDN1,AKT1 |
| GO:BP | negative regulation of cell growth | GO:0030308 | 0.008251967 | 2.083442542 | TP53,SIRT1,GJA1,ESR2,AGT |
| GO:BP | response to superoxide | GO:0000303 | 0.008357727 | 2.077911794 | NOS3,SOD1,MPO |
| GO:BP | regulation of ERK1 and ERK2 cascade | GO:0070372 | 0.008408358 | 2.075288792 | ADIPOQ,TNF,VEGFA,MAPK3 |
| GO:BP | positive regulation of protein kinase B signaling | GO:0051897 | 0.008441329 | 2.073589165 | TNF,VEGFA,EGF,PIK3CA |
| GO:BP | regulation of endothelial cell apoptotic process | GO:2000351 | 0.008571808 | 2.066927589 | TNF,IL10,IL4 |
| GO:BP | positive regulation of immunoglobulin production | GO:0002639 | 0.008571808 | 2.066927589 | IL6,IL10,IL4 |
| GO:BP | cellular response to steroid hormone stimulus | GO:0071383 | 0.008741017 | 2.058438032 | EDN1,ESR1,SIRT1,EP300,ESR2 |
| GO:BP | connective tissue development | GO:0061448 | 0.008753901 | 2.057798366 | MAPK3,EDN1,SIRT1,CTNNB1,PIK3CA |
| GO:BP | endocrine process | GO:0050886 | 0.008843469 | 2.053377356 | NOS3,EDN1,GJA1,AGT |
| GO:BP | regulation of endothelial cell differentiation | GO:0045601 | 0.00919347 | 2.036520544 | TNF,VEGFA,CTNNB1 |
| GO:BP | cellular response to transforming growth factor beta stimulus | GO:0071560 | 0.009244829 | 2.03410114 | EDN1,IL4,TP53,SIRT1,EP300 |
| GO:BP | positive regulation of fever generation | GO:0031622 | 0.009315103 | 2.030812349 | TNF,PTGS2 |
| GO:BP | response to oxygen radical | GO:0000305 | 0.009488614 | 2.022797224 | NOS3,SOD1,MPO |
| GO:BP | negative regulation of macroautophagy | GO:0016242 | 0.009826648 | 2.007594586 | AKT1,TP53,PIK3CA |
| GO:BP | negative regulation of cellular component organization | GO:0051129 | 0.00998343 | 2.000720213 | ADIPOQ,TNF,VEGFA,AKT1,TP53,PIK3CA,EP300 |
| GO:BP | neuron differentiation | GO:0030182 | 0.010155358 | 1.993304782 | NGF,CXCL12,IL6,VEGFA,EDN1,AKT1,CTNNB1,EP300,SOD1,AGT |
| GO:BP | positive regulation of receptor-mediated endocytosis | GO:0048260 | 0.010197589 | 1.991502486 | VEGFA,EGF,IL4 |
| GO:BP | negative regulation of binding | GO:0051100 | 0.010261293 | 1.988797891 | ADIPOQ,MAPK3,IL10,AKT1 |
| GO:BP | secretion by cell | GO:0032940 | 0.010294763 | 1.987383666 | ADIPOQ,CXCL12,TNF,IL6,EDN1,IL4,GJA1,AGT |
| GO:BP | response to transforming growth factor beta | GO:0071559 | 0.010430901 | 1.981678169 | EDN1,IL4,TP53,SIRT1,EP300 |
| GO:BP | urogenital system development | GO:0001655 | 0.010730485 | 1.969380655 | ADIPOQ,VEGFA,ESR1,CAT,CTNNB1,AGT |
| GO:BP | nitric oxide mediated signal transduction | GO:0007263 | 0.010807928 | 1.966257554 | NOS3,VEGFA,AGT |
| GO:BP | cell-cell junction organization | GO:0045216 | 0.010872098 | 1.963686653 | TNF,VEGFA,CTNNB1,GJA1,AGT |
| GO:BP | cellular response to insulin stimulus | GO:0032869 | 0.010872098 | 1.963686653 | ADIPOQ,AKT1,SIRT1,PIK3CA,AGT |
| GO:BP | regulation of protein-containing complex assembly | GO:0043254 | 0.010928619 | 1.961434714 | TNF,VEGFA,ESR1,TP53,EP300,MMP3 |
| GO:BP | detection of mechanical stimulus involved in sensory perception of pain | GO:0050966 | 0.01100771 | 1.958303016 | CXCL12,TNF |
| GO:BP | regulation of hemopoiesis | GO:1903706 | 0.011016629 | 1.957951274 | ADIPOQ,TNF,IL10,IL4,CTNNB1,SOD1 |
| GO:BP | organelle organization | GO:0006996 | 0.011050241 | 1.956628255 | NOS3,CXCL12,TNF,MAPK3,EDN1,EGF,ESR1,AKT1,TP53,SIRT1,CTNNB1,PIK3CA,GJA1,EP300 |
| GO:BP | endothelial cell apoptotic process | GO:0072577 | 0.011387488 | 1.943572066 | TNF,IL10,IL4 |
| GO:BP | regulation of heart contraction | GO:0008016 | 0.011411536 | 1.942655909 | NOS3,TNF,EDN1,GJA1,AGT |
| GO:BP | protein deacylation | GO:0035601 | 0.011550736 | 1.937390333 | VEGFA,TP53,SIRT1,EP300 |
| GO:BP | positive regulation of nuclear division | GO:0051785 | 0.011617883 | 1.934873015 | TNF,EDN1,EGF |
| GO:BP | mesenchyme development | GO:0060485 | 0.011835033 | 1.926830541 | NOS3,IL6,MAPK3,EDN1,CTNNB1 |
| GO:BP | sensory perception | GO:0007600 | 0.011955493 | 1.922432515 | CXCL12,TNF,MAPK3,IL10,PTGS2,EDN1 |
| GO:BP | regulation of transmembrane transport | GO:0034762 | 0.012030202 | 1.919727069 | ADIPOQ,TNF,EDN1,AKT1,GJA1,CYBB,AGT |
| GO:BP | regulation of oxidative stress-induced intrinsic apoptotic signaling pathway | GO:1902175 | 0.012044682 | 1.919204676 | AKT1,SIRT1,SOD1 |
| GO:BP | response to angiotensin | GO:1990776 | 0.01209409 | 1.917426786 | PTGS2,CYBB,AGT |
| GO:BP | positive regulation of RNA polymerase II transcription preinitiation complex assembly | GO:0045899 | 0.012338311 | 1.908744271 | ESR1,TP53 |
| GO:BP | regulation of macrophage activation | GO:0043030 | 0.012665818 | 1.897366757 | IL6,IL10,IL4 |
| GO:BP | cellular chemical homeostasis | GO:0055082 | 0.012730823 | 1.89514353 | CXCL12,MAPK3,EDN1,ESR1,SIRT1,PIK3CA,SOD1,AGT |
| GO:BP | positive regulation of miRNA transcription | GO:1902895 | 0.013046057 | 1.884520731 | TNF,IL10,TP53 |
| GO:BP | regulation of oxidoreductase activity | GO:0051341 | 0.013149184 | 1.881101197 | TNF,EDN1,AKT1,AGT |
| GO:BP | negative regulation of lipid metabolic process | GO:0045833 | 0.013326477 | 1.875284635 | TNF,AKT1,SIRT1,SOD1 |
| GO:BP | striated muscle tissue development | GO:0014706 | 0.013393523 | 1.873105174 | VEGFA,EDN1,CTNNB1,GJA1,EP300,AGT |
| GO:BP | negative regulation of wound healing | GO:0061045 | 0.013581536 | 1.867051096 | NOS3,TNF,EDN1 |
| GO:BP | regulation of systemic arterial blood pressure | GO:0003073 | 0.013708357 | 1.863014605 | NOS3,TNF,EDN1,AGT |
| GO:BP | ureteric bud development | GO:0001657 | 0.013708357 | 1.863014605 | VEGFA,CAT,CTNNB1,AGT |
| GO:BP | regulation of steroid metabolic process | GO:0019218 | 0.013708357 | 1.863014605 | TNF,SIRT1,SOD1,AGT |
| GO:BP | macromolecule deacylation | GO:0098732 | 0.013716687 | 1.862750776 | VEGFA,TP53,SIRT1,EP300 |
| GO:BP | negative regulation of helicase activity | GO:0051097 | 0.013788565 | 1.860480918 | TP53,SIRT1 |
| GO:BP | negative regulation of tumor necrosis factor production | GO:0032720 | 0.01403565 | 1.852767477 | ADIPOQ,IL10,IL4 |
| GO:BP | regulation of extent of cell growth | GO:0061387 | 0.014040109 | 1.852629523 | NGF,CXCL12,VEGFA |
| GO:BP | negative regulation of neurogenesis | GO:0050768 | 0.01415645 | 1.849045649 | TNF,IL6,TP53,CTNNB1 |
| GO:BP | mesonephric tubule development | GO:0072164 | 0.014284905 | 1.845122634 | VEGFA,CAT,CTNNB1,AGT |
| GO:BP | mesonephric epithelium development | GO:0072163 | 0.014284905 | 1.845122634 | VEGFA,CAT,CTNNB1,AGT |
| GO:BP | regulation of endocytosis | GO:0030100 | 0.014522015 | 1.837973105 | ADIPOQ,VEGFA,EGF,IL4 |
| GO:BP | regulation of cellular ketone metabolic process | GO:0010565 | 0.014620107 | 1.835049457 | ADIPOQ,PTGS2,AKT1,SIRT1 |
| GO:BP | regulation of immunoglobulin mediated immune response | GO:0002889 | 0.014755836 | 1.831036187 | TNF,IL10,IL4 |
| GO:BP | regulation of B cell mediated immunity | GO:0002712 | 0.014755836 | 1.831036187 | TNF,IL10,IL4 |
| GO:BP | response to amino acid | GO:0043200 | 0.014798437 | 1.829784142 | TNF,EDN1,PIK3CA,CYBB |
| GO:BP | positive regulation of glial cell proliferation | GO:0060252 | 0.015404931 | 1.812340237 | TNF,IL6 |
| GO:BP | negative regulation of tumor necrosis factor superfamily cytokine production | GO:1903556 | 0.015500044 | 1.809667064 | ADIPOQ,IL10,IL4 |
| GO:BP | cell migration involved in sprouting angiogenesis | GO:0002042 | 0.015631625 | 1.805995872 | VEGFA,PTGS2,AKT1 |
| GO:BP | regulation of DNA binding | GO:0051101 | 0.015654575 | 1.805358725 | NGF,EGF,CTNNB1,EP300 |
| GO:BP | positive regulation of heat generation | GO:0031652 | 0.015959636 | 1.796977028 | TNF,PTGS2 |
| GO:BP | regulation of fever generation | GO:0031620 | 0.015959636 | 1.796977028 | TNF,PTGS2 |
| GO:BP | export from cell | GO:0140352 | 0.016247009 | 1.789226571 | ADIPOQ,CXCL12,TNF,IL6,EDN1,IL4 |
| GO:BP | negative regulation of nervous system development | GO:0051961 | 0.016297788 | 1.787871341 | TNF,IL6,TP53,CTNNB1 |
| GO:BP | regulation of cell-cell adhesion involved in gastrulation | GO:0070587 | 0.016321837 | 1.78723096 | ADIPOQ,IL10 |
| GO:BP | negative regulation of heterotypic cell-cell adhesion | GO:0034115 | 0.016321837 | 1.78723096 | ADIPOQ,IL10 |
| GO:BP | histone modification | GO:0016570 | 0.01633572 | 1.786861723 | VEGFA,MAPK3,TP53,SIRT1,CTNNB1,EP300 |
| GO:BP | response to ozone | GO:0010193 | 0.016444823 | 1.783970786 | EDN1,CAT |
| GO:BP | regulation of cellular response to transforming growth factor beta stimulus | GO:1903844 | 0.016695946 | 1.777388981 | IL4,TP53,SIRT1,EP300 |
| GO:BP | mesonephros development | GO:0001823 | 0.016771748 | 1.775421676 | VEGFA,CAT,CTNNB1,AGT |
| GO:BP | liver development | GO:0001889 | 0.016905117 | 1.771981822 | TNF,IL6,IL10,PIK3CA |
| GO:BP | regulation of calcium ion import | GO:0090279 | 0.016970442 | 1.770306846 | CXCL12,EGF,CTNNB1 |
| GO:BP | response to carbon monoxide | GO:0034465 | 0.016982242 | 1.770004976 | IL10,SOD1 |
| GO:BP | regulation of glucose import | GO:0046324 | 0.017453102 | 1.758127382 | ADIPOQ,TNF,AKT1 |
| GO:BP | positive regulation of epithelial tube formation | GO:1905278 | 0.017602287 | 1.754430909 | VEGFA,EGF |
| GO:BP | regulation of epithelial tube formation | GO:1905276 | 0.017602287 | 1.754430909 | VEGFA,EGF |
| GO:BP | negative regulation of I-kappaB kinase/NF-kappaB signaling | GO:0043124 | 0.017656356 | 1.753098929 | ADIPOQ,ESR1,SIRT1 |
| GO:BP | positive regulation of neuroinflammatory response | GO:0150078 | 0.017772102 | 1.750261195 | TNF,IL6 |
| GO:BP | positive regulation of tyrosine phosphorylation of STAT protein | GO:0042531 | 0.017880611 | 1.747617634 | TNF,IL6,IL4 |
| GO:BP | muscle tissue development | GO:0060537 | 0.018107603 | 1.742139047 | VEGFA,EDN1,CTNNB1,GJA1,EP300,AGT |
| GO:BP | hepaticobiliary system development | GO:0061008 | 0.018416971 | 1.734781801 | TNF,IL6,IL10,PIK3CA |
| GO:BP | positive regulation of transcription from RNA polymerase II promoter in response to hypoxia | GO:0061419 | 0.018498079 | 1.732873361 | VEGFA,TP53 |
| GO:BP | neural precursor cell proliferation | GO:0061351 | 0.018669473 | 1.728867943 | VEGFA,EGF,TP53,CTNNB1 |
| GO:BP | metal ion transport | GO:0030001 | 0.018822437 | 1.725324148 | NOS3,CXCL12,PTGS2,EDN1,EGF,AKT1,CTNNB1 |
| GO:BP | macrophage differentiation | GO:0030225 | 0.018822698 | 1.725318135 | ADIPOQ,VEGFA,SIRT1 |
| GO:BP | ovulation cycle process | GO:0022602 | 0.018822698 | 1.725318135 | NOS3,ESR1,SIRT1 |
| GO:BP | epidermal growth factor receptor signaling pathway | GO:0007173 | 0.018836376 | 1.725002657 | EGF,AKT1,PIK3CA,AGT |
| GO:BP | behavior | GO:0007610 | 0.019054788 | 1.719995881 | NGF,CXCL12,PTGS2,TP53,EP300,SOD1,AGT |
| GO:BP | regulation of immune effector process | GO:0002697 | 0.019291417 | 1.714635862 | TNF,IL6,IL10,IL4,SIRT1 |
| GO:BP | maternal process involved in female pregnancy | GO:0060135 | 0.019409574 | 1.711984 | PTGS2,ESR1,AKT1 |
| GO:BP | lipid transport | GO:0006869 | 0.019474949 | 1.71052367 | ADIPOQ,EDN1,EGF,AKT1,SIRT1,AGT |
| GO:BP | cell-cell adhesion involved in gastrulation | GO:0070586 | 0.019943852 | 1.700190957 | ADIPOQ,IL10 |
| GO:BP | interleukin-18 production | GO:0032621 | 0.019943852 | 1.700190957 | TNF,IL10 |
| GO:BP | regulation of interleukin-18 production | GO:0032661 | 0.019943852 | 1.700190957 | TNF,IL10 |
| GO:BP | negative regulation of receptor binding | GO:1900121 | 0.019943852 | 1.700190957 | ADIPOQ,IL10 |
| GO:BP | prostaglandin metabolic process | GO:0006693 | 0.020038822 | 1.698127817 | PTGS2,EDN1,SIRT1 |
| GO:BP | unsaturated fatty acid biosynthetic process | GO:0006636 | 0.020038822 | 1.698127817 | PTGS2,EDN1,SIRT1 |
| GO:BP | prostanoid metabolic process | GO:0006692 | 0.020038822 | 1.698127817 | PTGS2,EDN1,SIRT1 |
| GO:BP | regulation of renal system process | GO:0098801 | 0.020052454 | 1.697832461 | ADIPOQ,EDN1,AGT |
| GO:BP | regulation of kidney development | GO:0090183 | 0.020052454 | 1.697832461 | ADIPOQ,VEGFA,AGT |
| GO:BP | positive regulation of I-kappaB phosphorylation | GO:1903721 | 0.020307754 | 1.692338115 | TNF,AKT1 |
| GO:BP | positive regulation of mononuclear cell migration | GO:0071677 | 0.020491061 | 1.688435556 | CXCL12,TNF,IL4 |
| GO:BP | regulation of muscle system process | GO:0090257 | 0.020531429 | 1.687580824 | NOS3,PTGS2,EDN1,SOD1,AGT |
| GO:BP | sexual reproduction | GO:0019953 | 0.020589773 | 1.686348445 | NOS3,PTGS2,EDN1,AKT1,SIRT1,CTNNB1,GJA1,SOD1 |
| GO:BP | positive regulation of cAMP-dependent protein kinase activity | GO:2000481 | 0.020671701 | 1.68462378 | ADIPOQ,SIRT1 |
| GO:BP | negative regulation of myeloid leukocyte differentiation | GO:0002762 | 0.020739617 | 1.683199276 | ADIPOQ,IL4,CTNNB1 |
| GO:BP | cellular response to starvation | GO:0009267 | 0.020944532 | 1.67892933 | ALB,MAPK3,TP53,SIRT1 |
| GO:BP | myeloid leukocyte activation | GO:0002274 | 0.021337872 | 1.670848897 | TNF,IL6,IL10,IL4 |
| GO:BP | intracellular steroid hormone receptor signaling pathway | GO:0030518 | 0.021364737 | 1.670302454 | ESR1,SIRT1,EP300,ESR2 |
| GO:BP | cellular carbohydrate metabolic process | GO:0044262 | 0.021715497 | 1.663230229 | ADIPOQ,AKT1,TP53,SIRT1,EP300 |
| GO:BP | smooth muscle contraction | GO:0006939 | 0.02187362 | 1.660079332 | PTGS2,EDN1,SOD1,AGT |
| GO:BP | regulation of leukocyte mediated immunity | GO:0002703 | 0.022091572 | 1.655773372 | TNF,IL6,IL10,IL4 |
| GO:BP | regulation of myelination | GO:0031641 | 0.022108845 | 1.655433954 | TNF,AKT1,CTNNB1 |
| GO:BP | regulation of plasma membrane bounded cell projection organization | GO:0120035 | 0.022592525 | 1.646035227 | NGF,CXCL12,VEGFA,AKT1,PIK3CA,EP300,AGT |
| GO:BP | positive regulation of protein localization to early endosome | GO:1902966 | 0.022623619 | 1.645437922 | VEGFA,EGF |
| GO:BP | regulation of protein localization to early endosome | GO:1902965 | 0.022623619 | 1.645437922 | VEGFA,EGF |
| GO:BP | brown fat cell differentiation | GO:0050873 | 0.022624418 | 1.64542259 | ADIPOQ,PTGS2,SIRT1 |
| GO:BP | regulation of lipid storage | GO:0010883 | 0.022624418 | 1.64542259 | TNF,IL6,SIRT1 |
| GO:BP | trachea formation | GO:0060440 | 0.022965643 | 1.638921389 | MAPK3,CTNNB1 |
| GO:BP | regulation of branching involved in lung morphogenesis | GO:0061046 | 0.022965643 | 1.638921389 | TNF,CTNNB1 |
| GO:BP | glial cell development | GO:0021782 | 0.023314728 | 1.632369648 | TNF,IL6,AKT1,SOD1 |
| GO:BP | negative regulation of catabolic process | GO:0009895 | 0.023522905 | 1.628509038 | TNF,IL10,AKT1,TP53,PIK3CA |
| GO:BP | lung morphogenesis | GO:0060425 | 0.023536446 | 1.628259107 | TNF,MAPK3,CTNNB1 |
| GO:BP | induction of positive chemotaxis | GO:0050930 | 0.02368637 | 1.625501484 | CXCL12,VEGFA |
| GO:BP | fever generation | GO:0001660 | 0.024368864 | 1.613164724 | TNF,PTGS2 |
| GO:BP | regulation of telomerase activity | GO:0051972 | 0.025023591 | 1.601650361 | MAPK3,TP53,CTNNB1 |
| GO:BP | negative regulation of response to wounding | GO:1903035 | 0.025261298 | 1.597544337 | NOS3,TNF,EDN1 |
| GO:BP | lymphocyte costimulation | GO:0031294 | 0.025752298 | 1.589184009 | IL4,AKT1,PIK3CA |
| GO:BP | negative regulation of protein serine/threonine kinase activity | GO:0071901 | 0.026160499 | 1.582353981 | ADIPOQ,AKT1,SIRT1,AGT |
| GO:BP | negative regulation of phosphate metabolic process | GO:0045936 | 0.026164683 | 1.582284523 | ADIPOQ,TNF,AKT1,TP53,SIRT1,AGT |
| GO:BP | macrophage derived foam cell differentiation | GO:0010742 | 0.026171572 | 1.582170195 | ADIPOQ,EP300,AGT |
| GO:BP | foam cell differentiation | GO:0090077 | 0.026171572 | 1.582170195 | ADIPOQ,EP300,AGT |
| GO:BP | positive regulation of T-helper 2 cell cytokine production | GO:2000553 | 0.026388364 | 1.578587538 | IL6,IL4 |
| GO:BP | cold-induced thermogenesis | GO:0106106 | 0.026424758 | 1.577988975 | ADIPOQ,VEGFA,IL4,GJA1 |
| GO:BP | regulation of cold-induced thermogenesis | GO:0120161 | 0.026424758 | 1.577988975 | ADIPOQ,VEGFA,IL4,GJA1 |
| GO:BP | negative regulation of phosphorus metabolic process | GO:0010563 | 0.026524489 | 1.576352975 | ADIPOQ,TNF,AKT1,TP53,SIRT1,AGT |
| GO:BP | positive regulation of neuron apoptotic process | GO:0043525 | 0.026571445 | 1.575584823 | TNF,TP53,CTNNB1 |
| GO:BP | regulation of cell projection organization | GO:0031344 | 0.02666026 | 1.574135625 | NGF,CXCL12,VEGFA,AKT1,PIK3CA,EP300,AGT |
| GO:BP | cholesterol efflux | GO:0033344 | 0.026901118 | 1.570229665 | ADIPOQ,EGF,SIRT1 |
| GO:BP | icosanoid biosynthetic process | GO:0046456 | 0.026901118 | 1.570229665 | PTGS2,EDN1,SIRT1 |
| GO:BP | regulation of I-kappaB phosphorylation | GO:1903719 | 0.027064987 | 1.567592174 | TNF,AKT1 |
| GO:BP | nephron epithelium development | GO:0072009 | 0.027087029 | 1.567238632 | ADIPOQ,VEGFA,CTNNB1,AGT |
| GO:BP | insulin receptor signaling pathway | GO:0008286 | 0.027087029 | 1.567238632 | AKT1,SIRT1,PIK3CA,AGT |
| GO:BP | negative regulation of DNA-binding transcription factor activity | GO:0043433 | 0.02717669 | 1.565803435 | IL10,ESR1,CAT,SIRT1 |
| GO:BP | regulation of cellular respiration | GO:0043457 | 0.027414187 | 1.562024631 | TNF,IL4,PIK3CA |
| GO:BP | regulation of miRNA transcription | GO:1902893 | 0.028171798 | 1.550185433 | TNF,IL10,TP53 |
| GO:BP | receptor-mediated endocytosis | GO:0006898 | 0.028387828 | 1.546867841 | ADIPOQ,VEGFA,EGF,IL4 |
| GO:BP | cellular response to estradiol stimulus | GO:0071392 | 0.028715414 | 1.541884913 | IL10,ESR1,ESR2 |
| GO:BP | negative regulation of growth | GO:0045926 | 0.029050839 | 1.536841323 | TP53,SIRT1,GJA1,ESR2,AGT |
| GO:BP | interleukin-1 production | GO:0032612 | 0.029055253 | 1.536775345 | TNF,IL6,IL10 |
| GO:BP | regulation of interleukin-1 production | GO:0032652 | 0.029055253 | 1.536775345 | TNF,IL6,IL10 |
| GO:BP | regulation of heat generation | GO:0031650 | 0.029234292 | 1.53410742 | TNF,PTGS2 |
| GO:BP | positive regulation of protein localization | GO:1903829 | 0.0293976 | 1.531688131 | TNF,VEGFA,PTGS2,EGF,AKT1 |
| GO:BP | cell cycle process | GO:0022402 | 0.029444827 | 1.530990993 | TNF,EDN1,EGF,AKT1,TP53,SIRT1,CTNNB1,GJA1 |
| GO:BP | miRNA transcription | GO:0061614 | 0.029665119 | 1.527753908 | TNF,IL10,TP53 |
| GO:BP | immune effector process | GO:0002252 | 0.029781745 | 1.526049852 | TNF,IL6,IL10,IL4,TP53,SIRT1 |
| GO:BP | regulation of tyrosine phosphorylation of STAT protein | GO:0042509 | 0.029803495 | 1.525732811 | TNF,IL6,IL4 |
| GO:BP | detection of mechanical stimulus | GO:0050982 | 0.02985393 | 1.524998491 | CXCL12,TNF,CTNNB1 |
| GO:BP | ERBB signaling pathway | GO:0038127 | 0.030011877 | 1.522706836 | EGF,AKT1,PIK3CA,AGT |
| GO:BP | skeletal system development | GO:0001501 | 0.030184336 | 1.520218374 | MAPK3,EDN1,TP53,CTNNB1,GJA1,EP300 |
| GO:BP | regulation of endothelial cell development | GO:1901550 | 0.030667816 | 1.51331715 | TNF,VEGFA |
| GO:BP | regulation of establishment of endothelial barrier | GO:1903140 | 0.030667816 | 1.51331715 | TNF,VEGFA |
| GO:BP | regulation of systemic arterial blood pressure by hormone | GO:0001990 | 0.030866957 | 1.510506182 | NOS3,EDN1,AGT |
| GO:BP | positive regulation of neural precursor cell proliferation | GO:2000179 | 0.031590878 | 1.500438305 | VEGFA,EGF,CTNNB1 |
| GO:BP | intrinsic apoptotic signaling pathway in response to DNA damage by p53 class mediator | GO:0042771 | 0.032067895 | 1.49392955 | TP53,SIRT1,EP300 |
| GO:BP | tyrosine phosphorylation of STAT protein | GO:0007260 | 0.032193845 | 1.492227147 | TNF,IL6,IL4 |
| GO:BP | extrinsic apoptotic signaling pathway in absence of ligand | GO:0097192 | 0.032789986 | 1.484258767 | TNF,IL4,AKT1 |
| GO:BP | signal transduction in absence of ligand | GO:0038034 | 0.032789986 | 1.484258767 | TNF,IL4,AKT1 |
| GO:BP | regulation of neuron projection development | GO:0010975 | 0.033281102 | 1.477802303 | NGF,CXCL12,VEGFA,AKT1,EP300,AGT |
| GO:BP | NIK/NF-kappaB signaling | GO:0038061 | 0.03356541 | 1.474108045 | TNF,EDN1,AKT1,EP300 |
| GO:BP | fatty acid metabolic process | GO:0006631 | 0.033680306 | 1.472623976 | ADIPOQ,PTGS2,EDN1,AKT1,SIRT1 |
| GO:BP | regulation of gluconeogenesis | GO:0006111 | 0.034181363 | 1.46621063 | ADIPOQ,SIRT1,EP300 |
| GO:BP | regulation of RNA polymerase II transcription preinitiation complex assembly | GO:0045898 | 0.034494729 | 1.462247267 | ESR1,TP53 |
| GO:BP | neuron projection extension | GO:1990138 | 0.034532299 | 1.461774506 | CXCL12,VEGFA,EDN1,CTNNB1 |
| GO:BP | regulation of T cell proliferation | GO:0042129 | 0.034532299 | 1.461774506 | IL6,IL10,IL4,CTNNB1 |
| GO:BP | protein localization to early endosome | GO:1902946 | 0.034539759 | 1.461680696 | VEGFA,EGF |
| GO:BP | second-messenger-mediated signaling | GO:0019932 | 0.034988947 | 1.456069125 | NOS3,TNF,VEGFA,EDN1 |
| GO:BP | astrocyte activation | GO:0048143 | 0.035510402 | 1.449644417 | TNF,IL6 |
| GO:BP | negative regulation of lipid storage | GO:0010888 | 0.035510402 | 1.449644417 | TNF,IL6 |
| GO:BP | T cell differentiation | GO:0030217 | 0.035798533 | 1.446134773 | IL6,IL4,TP53,CTNNB1,SOD1 |
| GO:BP | lipid biosynthetic process | GO:0008610 | 0.03684275 | 1.433647966 | TNF,PTGS2,EDN1,AKT1,SIRT1,PIK3CA,SOD1 |
| GO:BP | adaptive thermogenesis | GO:1990845 | 0.036965442 | 1.432204098 | ADIPOQ,VEGFA,IL4,GJA1 |
| GO:BP | protein localization to organelle | GO:0033365 | 0.036974599 | 1.432096525 | VEGFA,IL10,PTGS2,EGF,ESR1,AKT1,TP53 |
| GO:BP | glucose import | GO:0046323 | 0.037272861 | 1.42860727 | ADIPOQ,TNF,AKT1 |
| GO:BP | protein autophosphorylation | GO:0046777 | 0.037487696 | 1.426111256 | ADIPOQ,VEGFA,MAPK3,AKT1 |
| GO:BP | positive regulation of heterotypic cell-cell adhesion | GO:0034116 | 0.03803601 | 1.419805043 | TNF,IL10 |
| GO:BP | lymphocyte activation involved in immune response | GO:0002285 | 0.038319803 | 1.416576728 | IL6,IL10,IL4,TP53 |
| GO:BP | stress-induced premature senescence | GO:0090400 | 0.038545597 | 1.414025227 | TP53,SIRT1 |
| GO:BP | regulation of endodeoxyribonuclease activity | GO:0032071 | 0.038545597 | 1.414025227 | AKT1,SIRT1 |
| GO:BP | vascular wound healing | GO:0061042 | 0.038545597 | 1.414025227 | TNF,VEGFA |
| GO:BP | regulation of cellular component biogenesis | GO:0044087 | 0.038619897 | 1.413188893 | TNF,VEGFA,ESR1,TP53,PIK3CA,EP300,MMP3,AGT |
| GO:BP | negative regulation of carbohydrate metabolic process | GO:0045912 | 0.038679439 | 1.412519832 | ADIPOQ,TP53,EP300 |
| GO:BP | regulation of protein deacetylation | GO:0090311 | 0.038679439 | 1.412519832 | VEGFA,TP53,EP300 |
| GO:BP | regulation of cellular component size | GO:0032535 | 0.03877207 | 1.411481011 | NGF,CXCL12,VEGFA,EDN1,PIK3CA |
| GO:BP | regulation of axonogenesis | GO:0050770 | 0.038967954 | 1.409292391 | NGF,CXCL12,VEGFA |
| GO:BP | positive regulation of leukocyte adhesion to vascular endothelial cell | GO:1904996 | 0.039055248 | 1.408320605 | TNF,IL6 |
| GO:BP | detection of stimulus involved in sensory perception of pain | GO:0062149 | 0.039253442 | 1.40612225 | CXCL12,TNF |
| GO:BP | negative regulation of transferase activity | GO:0051348 | 0.039379586 | 1.404728855 | ADIPOQ,AKT1,TP53,SIRT1,AGT |
| GO:BP | steroid hormone mediated signaling pathway | GO:0043401 | 0.040004599 | 1.397890077 | ESR1,SIRT1,EP300,ESR2 |
| GO:BP | B cell activation involved in immune response | GO:0002312 | 0.040115589 | 1.396686831 | IL6,IL10,IL4 |
| GO:BP | striated muscle cell differentiation | GO:0051146 | 0.041599392 | 1.380913012 | VEGFA,EDN1,IL4,AKT1,AGT |
| GO:BP | neuron migration | GO:0001764 | 0.04174453 | 1.379400427 | CXCL12,VEGFA,CTNNB1,GJA1 |
| GO:BP | regulation of sterol transport | GO:0032371 | 0.042847756 | 1.36807192 | ADIPOQ,EGF,SIRT1 |
| GO:BP | regulation of cholesterol transport | GO:0032374 | 0.042847756 | 1.36807192 | ADIPOQ,EGF,SIRT1 |
| GO:BP | smooth muscle cell differentiation | GO:0051145 | 0.043424978 | 1.362260392 | VEGFA,SIRT1,CTNNB1 |
| GO:BP | positive regulation of lymphocyte activation | GO:0051251 | 0.043721003 | 1.35930988 | IL6,IL10,IL4,AKT1,PIK3CA |
| GO:BP | response to amyloid-beta | GO:1904645 | 0.044249187 | 1.3540947 | TNF,GJA1,MMP3 |
| GO:BP | positive regulation of cell cycle process | GO:0090068 | 0.044456521 | 1.352064527 | TNF,EDN1,EGF,AKT1 |
| GO:BP | negative regulation of cell cycle | GO:0045786 | 0.044788142 | 1.348836949 | TNF,IL10,PTGS2,TP53,CTNNB1 |
| GO:BP | response to nutrient | GO:0007584 | 0.045639858 | 1.340655716 | ADIPOQ,PTGS2,CAT,CYBB |
| GO:BP | neuroblast proliferation | GO:0007405 | 0.045643407 | 1.340621943 | VEGFA,TP53,CTNNB1 |
| GO:BP | sensory organ morphogenesis | GO:0090596 | 0.045787519 | 1.339252891 | VEGFA,MAPK3,EDN1,CTNNB1,SOD1 |
| GO:BP | cell morphogenesis involved in differentiation | GO:0000904 | 0.045845381 | 1.338704413 | NGF,CXCL12,VEGFA,EDN1,CTNNB1,EP300,SOD1 |
| GO:BP | intrinsic apoptotic signaling pathway in response to oxidative stress | GO:0008631 | 0.046138325 | 1.335938177 | AKT1,SIRT1,SOD1 |
| GO:BP | epithelial cell proliferation involved in liver morphogenesis | GO:0072575 | 0.046648614 | 1.331161252 | TNF,IL6 |
| GO:BP | hepatocyte proliferation | GO:0072574 | 0.046648614 | 1.331161252 | TNF,IL6 |
| GO:BP | positive regulation of cytokine production involved in inflammatory response | GO:1900017 | 0.046648614 | 1.331161252 | TNF,IL6 |
| GO:BP | positive regulation of inflammatory response | GO:0050729 | 0.046827989 | 1.329494492 | TNF,IL6,PTGS2,AGT |
| GO:BP | organic hydroxy compound transport | GO:0015850 | 0.04715859 | 1.326439192 | ADIPOQ,CXCL12,EGF,SIRT1,AGT |
| GO:BP | response to starvation | GO:0042594 | 0.047238271 | 1.325706009 | ALB,MAPK3,TP53,SIRT1 |
| GO:BP | B cell activation | GO:0042113 | 0.048624357 | 1.313146128 | IL6,IL10,IL4,TP53,EP300 |
| GO:BP | positive regulation of protein localization to endosome | GO:1905668 | 0.0489495 | 1.31025174 | VEGFA,EGF |
| GO:BP | positive regulation of myeloid cell apoptotic process | GO:0033034 | 0.049531918 | 1.305114856 | ADIPOQ,SIRT1 |
| GO:BP | homeostasis of number of cells | GO:0048872 | 0.04983725 | 1.302445933 | NOS3,IL6,VEGFA,AKT1,SOD1 |
| GO:CC | extracellular space | GO:0005615 | 2.71E-05 | 4.566706748 | NGF,ADIPOQ,CXCL12,ALB,TNF,IL6,VEGFA,IL10,EDN1,EGF,IL4,CAT,CTNNB1,MMP3,SOD1,AGT,MPO |
| GO:CC | endomembrane system | GO:0012505 | 3.35E-05 | 4.475189722 | NGF,ADIPOQ,NOS3,ALB,TNF,IL6,VEGFA,MAPK3,PTGS2,EDN1,EGF,ESR1,CAT,TP53,SIRT1 |
| GO:CC | endoplasmic reticulum lumen | GO:0005788 | 9.14E-05 | 4.038855585 | ALB,IL6,MAPK3,PTGS2,EDN1 |
| GO:CC | membrane raft | GO:0045121 | 0.000153946 | 3.812630468 | NOS3,TNF,MAPK3,PTGS2,CTNNB1,GJA1 |
| GO:CC | membrane microdomain | GO:0098857 | 0.000156688 | 3.80496323 | NOS3,TNF,MAPK3,PTGS2,CTNNB1,GJA1 |
| GO:CC | caveola | GO:0005901 | 0.000187374 | 3.727291385 | NOS3,MAPK3,PTGS2,CTNNB1 |
| GO:CC | vesicle | GO:0031982 | 0.000297136 | 3.52704478 | NGF,NOS3,CXCL12,ALB,TNF,VEGFA,MAPK3,EDN1,EGF,AKT1,CAT,CTNNB1,GJA1,CYBB,SOD1,AGT,MPO |
| GO:CC | plasma membrane raft | GO:0044853 | 0.000677235 | 3.169260728 | NOS3,MAPK3,PTGS2,CTNNB1 |
| GO:CC | extracellular region | GO:0005576 | 0.00079512 | 3.099567229 | NGF,ADIPOQ,CXCL12,ALB,TNF,IL6,VEGFA,IL10,EDN1,EGF,IL4 |
| GO:CC | secretory vesicle | GO:0099503 | 0.001033573 | 2.985658751 | NGF,ALB,VEGFA,EDN1,EGF,CAT,CYBB,SOD1,MPO |
| GO:CC | cytoplasmic vesicle | GO:0031410 | 0.001176538 | 2.929394197 | NGF,NOS3,ALB,TNF,VEGFA,MAPK3,EDN1,EGF,CAT,GJA1,CYBB,SOD1,MPO |
| GO:CC | intracellular vesicle | GO:0097708 | 0.00118733 | 2.925428419 | NGF,NOS3,ALB,TNF,VEGFA,MAPK3,EDN1,EGF,CAT,GJA1,CYBB,SOD1,MPO |
| GO:CC | platelet alpha granule lumen | GO:0031093 | 0.001606175 | 2.794207037 | ALB,VEGFA,EGF |
| GO:CC | secretory granule | GO:0030141 | 0.00258606 | 2.587361355 | ALB,VEGFA,EDN1,EGF,CAT,CYBB,SOD1,MPO |
| GO:CC | anchoring junction | GO:0070161 | 0.002769952 | 2.557527796 | VEGFA,MAPK3,AKT1,CAT,CTNNB1,PIK3CA,GJA1 |
| GO:CC | cytoplasm | GO:0005737 | 0.00322968 | 2.49084051 | NGF,ADIPOQ,NOS3,CXCL12,ALB,TNF,IL6,VEGFA,MAPK3,IL10,PTGS2,EDN1,EGF,ESR1,AKT1,CAT,TP53,SIRT1,CTNNB1,PIK3CA,GJA1,EP300,CYBB,ESR2,SOD1,AGT,MPO |
| GO:CC | cell periphery | GO:0071944 | 0.0038036 | 2.419805197 | ADIPOQ,NOS3,CXCL12,TNF,IL6,VEGFA,MAPK3,PTGS2,EGF,ESR1,AKT1,CAT,CTNNB1,PIK3CA,GJA1,CYBB,MMP3,SOD1,AGT |
| GO:CC | intercalated disc | GO:0014704 | 0.003897725 | 2.409188807 | CTNNB1,PIK3CA,GJA1 |
| GO:CC | platelet alpha granule | GO:0031091 | 0.004089394 | 2.388341024 | ALB,VEGFA,EGF |
| GO:CC | endoplasmic reticulum | GO:0005783 | 0.005605682 | 2.251371575 | ADIPOQ,ALB,IL6,MAPK3,PTGS2,EDN1,CAT,TP53,GJA1,CYBB |
| GO:CC | fascia adherens | GO:0005916 | 0.00937218 | 2.02815939 | CTNNB1,GJA1 |
| GO:CC | cell-cell contact zone | GO:0044291 | 0.011473767 | 1.940293975 | CTNNB1,PIK3CA,GJA1 |
| GO:CC | secretory granule lumen | GO:0034774 | 0.012846715 | 1.891207925 | ALB,VEGFA,EGF,CAT,MPO |
| GO:CC | cytoplasmic vesicle lumen | GO:0060205 | 0.013426855 | 1.872025716 | ALB,VEGFA,EGF,CAT,MPO |
| GO:CC | vesicle lumen | GO:0031983 | 0.013824735 | 1.859343196 | ALB,VEGFA,EGF,CAT,MPO |
| GO:CC | intracellular organelle lumen | GO:0070013 | 0.020376054 | 1.69087992 | NGF,ALB,IL6,VEGFA,MAPK3,PTGS2,EDN1,EGF,ESR1,AKT1,CAT,TP53,SIRT1,CTNNB1,GJA1,EP300,ESR2,SOD1,MPO |
| GO:CC | organelle lumen | GO:0043233 | 0.020422754 | 1.689885693 | NGF,ALB,IL6,VEGFA,MAPK3,PTGS2,EDN1,EGF,ESR1,AKT1,CAT,TP53,SIRT1,CTNNB1,GJA1,EP300,ESR2,SOD1,MPO |
| GO:CC | membrane-enclosed lumen | GO:0031974 | 0.020422754 | 1.689885693 | NGF,ALB,IL6,VEGFA,MAPK3,PTGS2,EDN1,EGF,ESR1,AKT1,CAT,TP53,SIRT1,CTNNB1,GJA1,EP300,ESR2,SOD1,MPO |
| GO:CC | cell-cell junction | GO:0005911 | 0.024744215 | 1.606526314 | VEGFA,AKT1,CTNNB1,PIK3CA,GJA1 |
| GO:CC | cell surface | GO:0009986 | 0.039824524 | 1.399849411 | ADIPOQ,CXCL12,TNF,VEGFA |
| GO:CC | Golgi apparatus | GO:0005794 | 0.047667172 | 1.321780611 | NGF,NOS3,ALB,MAPK3,ESR1,CAT,GJA1,CYBB |
