# Supplementary material for: Exploring the Mechanism of Action of Berberine on Arrhythmia After Myocardial Infarction: A Network Pharmacology, Molecular Docking, and Cellular Experimental Study
Source: Cardiovasc Ther. 2025 Jun 2;2025:5632985. doi: 10.1155/cdr/5632985 (PMC12149515; doi:10.1155/cdr/5632985)
Supplement: Supporting Information 2 — Table S2: The results of pathway enrichment analysis including KEGG, REAC, and Wiki. [file 5632985.f2.docx]

| source | term_name | term_id | adjusted_p_value | negative_log10_of_adjusted_p_value | intersections |
| --- | --- | --- | --- | --- | --- |
| KEGG | HIF-1 signaling pathway | KEGG:04066 | 1.70E-11 | 10.77012827 | NOS3,IL6,VEGFA,MAPK3,EDN1,EGF,AKT1,PIK3CA,EP300,CYBB |
| KEGG | AGE-RAGE signaling pathway in diabetic complications | KEGG:04933 | 4.49E-11 | 10.34765609 | NOS3,TNF,IL6,VEGFA,MAPK3,EDN1,AKT1,PIK3CA,CYBB,AGT |
| KEGG | Pathways in cancer | KEGG:05200 | 1.08E-10 | 9.967770298 | CXCL12,IL6,VEGFA,MAPK3,PTGS2,EDN1,EGF,IL4,ESR1,AKT1,TP53,CTNNB1,PIK3CA,EP300,ESR2,AGT |
| KEGG | FoxO signaling pathway | KEGG:04068 | 3.03E-09 | 8.518270248 | IL6,MAPK3,IL10,EGF,AKT1,CAT,SIRT1,PIK3CA,EP300 |
| KEGG | Human cytomegalovirus infection | KEGG:05163 | 4.38E-09 | 8.358794543 | CXCL12,TNF,IL6,VEGFA,MAPK3,PTGS2,AKT1,TP53,CTNNB1,PIK3CA |
| KEGG | Prostate cancer | KEGG:05215 | 2.24E-08 | 7.650447988 | MAPK3,EGF,AKT1,TP53,CTNNB1,PIK3CA,EP300,MMP3 |
| KEGG | TNF signaling pathway | KEGG:04668 | 7.15E-08 | 7.145646794 | TNF,IL6,MAPK3,PTGS2,EDN1,AKT1,PIK3CA,MMP3 |
| KEGG | Fluid shear stress and atherosclerosis | KEGG:05418 | 7.57E-08 | 7.120874363 | NOS3,TNF,VEGFA,EDN1,AKT1,TP53,CTNNB1,PIK3CA |
| KEGG | Kaposi sarcoma-associated herpesvirus infection | KEGG:05167 | 1.03E-07 | 6.987024642 | IL6,VEGFA,MAPK3,PTGS2,AKT1,TP53,CTNNB1,PIK3CA,EP300 |
| KEGG | C-type lectin receptor signaling pathway | KEGG:04625 | 3.61E-07 | 6.442783931 | TNF,IL6,MAPK3,IL10,PTGS2,AKT1,PIK3CA |
| KEGG | Endometrial cancer | KEGG:05213 | 3.92E-07 | 6.406972625 | MAPK3,EGF,AKT1,TP53,CTNNB1,PIK3CA |
| KEGG | PI3K-Akt signaling pathway | KEGG:04151 | 3.95E-07 | 6.403725583 | NGF,NOS3,IL6,VEGFA,MAPK3,EGF,IL4,AKT1,TP53,PIK3CA |
| KEGG | VEGF signaling pathway | KEGG:04370 | 4.35E-07 | 6.361097065 | NOS3,VEGFA,MAPK3,PTGS2,AKT1,PIK3CA |
| KEGG | Lipid and atherosclerosis | KEGG:05417 | 5.86E-07 | 6.231988103 | NOS3,TNF,IL6,MAPK3,AKT1,TP53,PIK3CA,CYBB,MMP3 |
| KEGG | Human papillomavirus infection | KEGG:05165 | 6.37E-07 | 6.195727057 | TNF,VEGFA,MAPK3,PTGS2,EGF,AKT1,TP53,CTNNB1,PIK3CA,EP300 |
| KEGG | Breast cancer | KEGG:05224 | 8.91E-07 | 6.050007792 | MAPK3,EGF,ESR1,AKT1,TP53,CTNNB1,PIK3CA,ESR2 |
| KEGG | Proteoglycans in cancer | KEGG:05205 | 1.74E-06 | 5.75885394 | TNF,VEGFA,MAPK3,ESR1,AKT1,TP53,CTNNB1,PIK3CA |
| KEGG | Pancreatic cancer | KEGG:05212 | 2.06E-06 | 5.687094077 | VEGFA,MAPK3,EGF,AKT1,TP53,PIK3CA |
| KEGG | Thyroid hormone signaling pathway | KEGG:04919 | 2.15E-06 | 5.668041881 | MAPK3,ESR1,AKT1,TP53,CTNNB1,PIK3CA,EP300 |
| KEGG | EGFR tyrosine kinase inhibitor resistance | KEGG:01521 | 2.60E-06 | 5.58490011 | IL6,VEGFA,MAPK3,EGF,AKT1,PIK3CA |
| KEGG | Leishmaniasis | KEGG:05140 | 3.59E-06 | 5.444897346 | TNF,MAPK3,IL10,PTGS2,IL4,CYBB |
| KEGG | Colorectal cancer | KEGG:05210 | 4.35E-06 | 5.361558397 | MAPK3,EGF,AKT1,TP53,CTNNB1,PIK3CA |
| KEGG | Longevity regulating pathway | KEGG:04211 | 5.35E-06 | 5.271649755 | ADIPOQ,AKT1,CAT,TP53,SIRT1,PIK3CA |
| KEGG | Hepatitis C | KEGG:05160 | 6.37E-06 | 5.195667536 | TNF,MAPK3,EGF,AKT1,TP53,CTNNB1,PIK3CA |
| KEGG | Chagas disease | KEGG:05142 | 1.14E-05 | 4.941442769 | TNF,IL6,MAPK3,IL10,AKT1,PIK3CA |
| KEGG | T cell receptor signaling pathway | KEGG:04660 | 1.29E-05 | 4.890454081 | TNF,MAPK3,IL10,IL4,AKT1,PIK3CA |
| KEGG | Hepatitis B | KEGG:05161 | 1.54E-05 | 4.811161526 | TNF,IL6,MAPK3,AKT1,TP53,PIK3CA,EP300 |
| KEGG | JAK-STAT signaling pathway | KEGG:04630 | 1.61E-05 | 4.792747965 | IL6,IL10,EGF,IL4,AKT1,PIK3CA,EP300 |
| KEGG | IL-17 signaling pathway | KEGG:04657 | 2.05E-05 | 4.687216275 | TNF,IL6,MAPK3,PTGS2,IL4,MMP3 |
| KEGG | Sphingolipid signaling pathway | KEGG:04071 | 3.04E-05 | 4.516652089 | NOS3,TNF,MAPK3,AKT1,TP53,PIK3CA |
| KEGG | Endocrine resistance | KEGG:01522 | 3.21E-05 | 4.494147483 | MAPK3,ESR1,AKT1,TP53,PIK3CA,ESR2 |
| KEGG | Fc epsilon RI signaling pathway | KEGG:04664 | 4.59E-05 | 4.338420855 | TNF,MAPK3,IL4,AKT1,PIK3CA |
| KEGG | Rap1 signaling pathway | KEGG:04015 | 4.65E-05 | 4.332187457 | NGF,VEGFA,MAPK3,EGF,AKT1,CTNNB1,PIK3CA |
| KEGG | Relaxin signaling pathway | KEGG:04926 | 4.91E-05 | 4.309062196 | NOS3,VEGFA,MAPK3,EDN1,AKT1,PIK3CA |
| KEGG | Intestinal immune network for IgA production | KEGG:04672 | 5.94E-05 | 4.226437141 | CXCL12,IL6,IL10,IL4 |
| KEGG | Alcoholic liver disease | KEGG:04936 | 6.27E-05 | 4.203056073 | ADIPOQ,TNF,IL6,AKT1,SIRT1,CTNNB1 |
| KEGG | Apoptosis | KEGG:04210 | 6.42E-05 | 4.192515711 | NGF,TNF,MAPK3,AKT1,TP53,PIK3CA |
| KEGG | Rheumatoid arthritis | KEGG:05323 | 6.55E-05 | 4.184051981 | CXCL12,TNF,IL6,VEGFA |
| KEGG | Non-small cell lung cancer | KEGG:05223 | 6.59E-05 | 4.181058261 | MAPK3,EGF,AKT1,TP53,PIK3CA |
| KEGG | Melanoma | KEGG:05218 | 6.59E-05 | 4.181058261 | MAPK3,EGF,AKT1,TP53,PIK3CA |
| KEGG | Yersinia infection | KEGG:05135 | 6.70E-05 | 4.173624781 | TNF,IL6,MAPK3,IL10,AKT1,PIK3CA |
| KEGG | Renal cell carcinoma | KEGG:05211 | 8.07E-05 | 4.093254141 | VEGFA,MAPK3,AKT1,PIK3CA,EP300 |
| KEGG | Glioma | KEGG:05214 | 8.09E-05 | 4.092077972 | MAPK3,EGF,AKT1,TP53,PIK3CA |
| KEGG | Pertussis | KEGG:05133 | 0.000107007 | 3.97058695 | TNF,IL6,MAPK3,IL10 |
| KEGG | Insulin resistance | KEGG:04931 | 0.000109854 | 3.959182974 | NOS3,TNF,IL6,AKT1,PIK3CA,AGT |
| KEGG | Gastric cancer | KEGG:05226 | 0.000110215 | 3.957760281 | MAPK3,EGF,AKT1,TP53,CTNNB1,PIK3CA |
| KEGG | Longevity regulating pathway - multiple species | KEGG:04213 | 0.000118664 | 3.925679568 | AKT1,CAT,SIRT1,PIK3CA,SOD1 |
| KEGG | Bladder cancer | KEGG:05219 | 0.000122031 | 3.913528245 | VEGFA,MAPK3,EGF,TP53 |
| KEGG | Human T-cell leukemia virus 1 infection | KEGG:05166 | 0.000124388 | 3.905221722 | TNF,IL6,MAPK3,AKT1,TP53,PIK3CA,EP300 |
| KEGG | MAPK signaling pathway | KEGG:04010 | 0.000133306 | 3.875150208 | NGF,TNF,VEGFA,MAPK3,EGF,AKT1,TP53 |
| KEGG | Cellular senescence | KEGG:04218 | 0.000149998 | 3.823913646 | IL6,MAPK3,AKT1,TP53,SIRT1,PIK3CA |
| KEGG | Prolactin signaling pathway | KEGG:04917 | 0.000178756 | 3.74773868 | MAPK3,ESR1,AKT1,PIK3CA,ESR2 |
| KEGG | Inflammatory bowel disease | KEGG:05321 | 0.000218527 | 3.66049524 | TNF,IL6,IL10,IL4 |
| KEGG | Chemical carcinogenesis - receptor activation | KEGG:05207 | 0.000243013 | 3.614370521 | VEGFA,MAPK3,EGF,ESR1,AKT1,PIK3CA,ESR2 |
| KEGG | Estrogen signaling pathway | KEGG:04915 | 0.000276827 | 3.557790991 | NOS3,MAPK3,ESR1,AKT1,PIK3CA,ESR2 |
| KEGG | Viral protein interaction with cytokine and cytokine receptor | KEGG:04061 | 0.000297311 | 3.526788454 | CXCL12,TNF,IL6,IL10 |
| KEGG | Toll-like receptor signaling pathway | KEGG:04620 | 0.000373135 | 3.428134386 | TNF,IL6,MAPK3,AKT1,PIK3CA |
| KEGG | Type II diabetes mellitus | KEGG:04930 | 0.000377137 | 3.423500584 | ADIPOQ,TNF,MAPK3,PIK3CA |
| KEGG | Cytokine-cytokine receptor interaction | KEGG:04060 | 0.000378922 | 3.421450524 | NGF,CXCL12,TNF,IL6,IL10,IL4 |
| KEGG | Influenza A | KEGG:05164 | 0.000415171 | 3.381773298 | TNF,IL6,MAPK3,AKT1,PIK3CA,EP300 |
| KEGG | Chemical carcinogenesis - reactive oxygen species | KEGG:05208 | 0.000449039 | 3.347715513 | VEGFA,MAPK3,EGF,AKT1,CAT,PIK3CA,SOD1 |
| KEGG | Tuberculosis | KEGG:05152 | 0.000525513 | 3.279416687 | TNF,IL6,MAPK3,IL10,AKT1,EP300 |
| KEGG | Focal adhesion | KEGG:04510 | 0.00063358 | 3.198198629 | VEGFA,MAPK3,EGF,AKT1,CTNNB1,PIK3CA |
| KEGG | Alzheimer disease | KEGG:05010 | 0.000674628 | 3.170935436 | TNF,IL6,MAPK3,PTGS2,AKT1,CTNNB1,PIK3CA,CYBB |
| KEGG | African trypanosomiasis | KEGG:05143 | 0.000696425 | 3.157125605 | TNF,IL6,IL10 |
| KEGG | Neurotrophin signaling pathway | KEGG:04722 | 0.000795201 | 3.099523331 | NGF,MAPK3,AKT1,TP53,PIK3CA |
| KEGG | Asthma | KEGG:05310 | 0.000862745 | 3.064117403 | TNF,IL10,IL4 |
| KEGG | MicroRNAs in cancer | KEGG:05206 | 0.001238791 | 2.907002002 | VEGFA,MAPK3,PTGS2,TP53,SIRT1,PIK3CA,EP300 |
| KEGG | Ras signaling pathway | KEGG:04014 | 0.001444668 | 2.840231983 | NGF,VEGFA,MAPK3,EGF,AKT1,PIK3CA |
| KEGG | Allograft rejection | KEGG:05330 | 0.00175232 | 2.75638666 | TNF,IL10,IL4 |
| KEGG | Malaria | KEGG:05144 | 0.001781767 | 2.749149177 | TNF,IL6,IL10 |
| KEGG | Central carbon metabolism in cancer | KEGG:05230 | 0.002034212 | 2.691603709 | MAPK3,AKT1,TP53,PIK3CA |
| KEGG | Salmonella infection | KEGG:05132 | 0.002210963 | 2.655418564 | TNF,IL6,MAPK3,AKT1,CTNNB1,PIK3CA |
| KEGG | Platinum drug resistance | KEGG:01524 | 0.002274701 | 2.64307571 | MAPK3,AKT1,TP53,PIK3CA |
| KEGG | Chronic myeloid leukemia | KEGG:05220 | 0.002817457 | 2.550142779 | MAPK3,AKT1,TP53,PIK3CA |
| KEGG | Non-alcoholic fatty liver disease | KEGG:04932 | 0.002863001 | 2.543178554 | ADIPOQ,TNF,IL6,AKT1,PIK3CA |
| KEGG | GnRH secretion | KEGG:04929 | 0.00345229 | 2.461892704 | MAPK3,AKT1,PIK3CA,ESR2 |
| KEGG | Toxoplasmosis | KEGG:05145 | 0.003701131 | 2.431665501 | TNF,MAPK3,IL10,AKT1 |
| KEGG | Hepatocellular carcinoma | KEGG:05225 | 0.003977082 | 2.400435502 | MAPK3,AKT1,TP53,CTNNB1,PIK3CA |
| KEGG | ErbB signaling pathway | KEGG:04012 | 0.0041799 | 2.378834086 | MAPK3,EGF,AKT1,PIK3CA |
| KEGG | Coronavirus disease - COVID-19 | KEGG:05171 | 0.004279841 | 2.368572392 | TNF,IL6,MAPK3,PIK3CA,CYBB,MMP3 |
| KEGG | PD-L1 expression and PD-1 checkpoint pathway in cancer | KEGG:05235 | 0.005244457 | 2.280299494 | MAPK3,EGF,AKT1,PIK3CA |
| KEGG | Small cell lung cancer | KEGG:05222 | 0.005971066 | 2.223948127 | PTGS2,AKT1,TP53,PIK3CA |
| KEGG | Acute myeloid leukemia | KEGG:05221 | 0.006449973 | 2.19044212 | MAPK3,AKT1,PIK3CA,MPO |
| KEGG | Thyroid cancer | KEGG:05216 | 0.006969597 | 2.156792352 | MAPK3,TP53,CTNNB1 |
| KEGG | Choline metabolism in cancer | KEGG:05231 | 0.00764086 | 2.116857771 | MAPK3,EGF,AKT1,PIK3CA |
| KEGG | Pathways of neurodegeneration - multiple diseases | KEGG:05022 | 0.008330543 | 2.079326705 | TNF,IL6,MAPK3,PTGS2,CAT,CTNNB1,CYBB,SOD1 |
| KEGG | Amoebiasis | KEGG:05146 | 0.008592344 | 2.065888344 | TNF,IL6,IL10,PIK3CA |
| KEGG | Epstein-Barr virus infection | KEGG:05169 | 0.009188804 | 2.036741029 | TNF,IL6,AKT1,TP53,PIK3CA |
| KEGG | Phospholipase D signaling pathway | KEGG:04072 | 0.009660333 | 2.015007894 | MAPK3,EGF,AKT1,PIK3CA,AGT |
| KEGG | Melanogenesis | KEGG:04916 | 0.012468859 | 1.904173279 | MAPK3,EDN1,CTNNB1,EP300 |
| KEGG | Prion disease | KEGG:05020 | 0.016708092 | 1.777073154 | TNF,IL6,MAPK3,PIK3CA,CYBB,SOD1 |
| KEGG | AMPK signaling pathway | KEGG:04152 | 0.016736207 | 1.776342948 | ADIPOQ,AKT1,SIRT1,PIK3CA |
| KEGG | Hypertrophic cardiomyopathy | KEGG:05410 | 0.017651411 | 1.753220577 | TNF,IL6,EDN1,AGT |
| KEGG | Platelet activation | KEGG:04611 | 0.018983239 | 1.721629681 | NOS3,MAPK3,AKT1,PIK3CA |
| KEGG | Osteoclast differentiation | KEGG:04380 | 0.019577365 | 1.708245753 | TNF,MAPK3,AKT1,PIK3CA |
| KEGG | Antifolate resistance | KEGG:01523 | 0.020029213 | 1.69833611 | TNF,IL6 |
| KEGG | NF-kappa B signaling pathway | KEGG:04064 | 0.021845435 | 1.660639304 | CXCL12,TNF,PTGS2 |
| KEGG | Adipocytokine signaling pathway | KEGG:04920 | 0.02248751 | 1.648058623 | ADIPOQ,TNF,AKT1 |
| KEGG | Growth hormone synthesis, secretion and action | KEGG:04935 | 0.023433334 | 1.630165922 | MAPK3,AKT1,PIK3CA,EP300 |
| KEGG | Leukocyte transendothelial migration | KEGG:04670 | 0.023595966 | 1.627162236 | CXCL12,CTNNB1,PIK3CA,CYBB |
| KEGG | cAMP signaling pathway | KEGG:04024 | 0.023830468 | 1.622867432 | MAPK3,EDN1,AKT1,PIK3CA,EP300 |
| KEGG | Shigellosis | KEGG:05131 | 0.025818627 | 1.588066864 | TNF,MAPK3,AKT1,TP53,PIK3CA |
| KEGG | Regulation of lipolysis in adipocytes | KEGG:04923 | 0.028005039 | 1.552763818 | PTGS2,AKT1,PIK3CA |
| KEGG | Measles | KEGG:05162 | 0.029372358 | 1.532061186 | IL6,AKT1,TP53,PIK3CA |
| KEGG | Graft-versus-host disease | KEGG:05332 | 0.030576106 | 1.514617821 | TNF,IL6 |
| KEGG | Calcium signaling pathway | KEGG:04020 | 0.031769622 | 1.497987956 | NGF,NOS3,VEGFA,EGF |
| KEGG | Signaling pathways regulating pluripotency of stem cells | KEGG:04550 | 0.032718455 | 1.485207218 | MAPK3,AKT1,CTNNB1,PIK3CA |
| KEGG | Neutrophil extracellular trap formation | KEGG:04613 | 0.036926522 | 1.432661597 | MAPK3,AKT1,PIK3CA,CYBB,MPO |
| KEGG | Hematopoietic cell lineage | KEGG:04640 | 0.038059082 | 1.419541686 | TNF,IL6,IL4 |
| KEGG | Diabetic cardiomyopathy | KEGG:05415 | 0.043208878 | 1.36442701 | NOS3,AKT1,PIK3CA,CYBB,AGT |
| KEGG | mTOR signaling pathway | KEGG:04150 | 0.043321227 | 1.363299249 | TNF,MAPK3,AKT1,PIK3CA |
| REAC | Interleukin-4 and Interleukin-13 signaling | REAC:R-HSA-6785807 | 5.30E-10 | 9.275349009 | TNF,IL6,VEGFA,IL10,PTGS2,IL4,AKT1,TP53,MMP3 |
| REAC | Extra-nuclear estrogen signaling | REAC:R-HSA-9009391 | 1.46E-09 | 8.836095702 | NOS3,MAPK3,EGF,ESR1,AKT1,PIK3CA,MMP3,ESR2 |
| REAC | ESR-mediated signaling | REAC:R-HSA-8939211 | 1.23E-08 | 7.908880033 | NOS3,CXCL12,MAPK3,EGF,ESR1,AKT1,PIK3CA,EP300,MMP3,ESR2 |
| REAC | Signaling by Receptor Tyrosine Kinases | REAC:R-HSA-9006934 | 3.62E-08 | 7.441228157 | NGF,NOS3,CXCL12,VEGFA,MAPK3,EGF,ESR1,AKT1,CTNNB1,PIK3CA,EP300,CYBB |
| REAC | Signaling by Interleukins | REAC:R-HSA-449147 | 1.07E-07 | 6.972336605 | TNF,IL6,VEGFA,MAPK3,IL10,PTGS2,IL4,AKT1,TP53,PIK3CA,MMP3,SOD1 |
| REAC | Signaling by Nuclear Receptors | REAC:R-HSA-9006931 | 2.21E-07 | 6.655352136 | NOS3,CXCL12,MAPK3,EGF,ESR1,AKT1,PIK3CA,EP300,MMP3,ESR2 |
| REAC | Cytokine Signaling in Immune system | REAC:R-HSA-1280215 | 1.35E-05 | 4.868753933 | TNF,IL6,VEGFA,MAPK3,IL10,PTGS2,IL4,AKT1,TP53,PIK3CA,MMP3,SOD1 |
| REAC | PI5P, PP2A and IER3 Regulate PI3K/AKT Signaling | REAC:R-HSA-6811558 | 2.26E-05 | 4.645696675 | MAPK3,EGF,ESR1,AKT1,PIK3CA,ESR2 |
| REAC | Signaling by VEGF | REAC:R-HSA-194138 | 2.80E-05 | 4.552412879 | NOS3,VEGFA,AKT1,CTNNB1,PIK3CA,CYBB |
| REAC | Interleukin-10 signaling | REAC:R-HSA-6783783 | 2.93E-05 | 4.532740652 | TNF,IL6,IL10,PTGS2 |
| REAC | Signal Transduction | REAC:R-HSA-162582 | 3.19E-05 | 4.496396884 | NGF,NOS3,CXCL12,TNF,IL6,VEGFA,MAPK3,EDN1,EGF,ESR1,AKT1,TP53,CTNNB1,PIK3CA,GJA1,EP300,CYBB,MMP3,ESR2,AGT |
| REAC | Negative regulation of the PI3K/AKT network | REAC:R-HSA-199418 | 3.51E-05 | 4.454275201 | MAPK3,EGF,ESR1,AKT1,PIK3CA,ESR2 |
| REAC | Immune System | REAC:R-HSA-168256 | 0.000129667 | 3.887170798 | NOS3,TNF,IL6,VEGFA,MAPK3,IL10,PTGS2,IL4,AKT1,CAT,TP53,CTNNB1,PIK3CA,EP300,CYBB,MMP3,SOD1,MPO |
| REAC | PIP3 activates AKT signaling | REAC:R-HSA-1257604 | 0.000543766 | 3.264588282 | MAPK3,EGF,ESR1,AKT1,TP53,PIK3CA,ESR2 |
| REAC | VEGFA-VEGFR2 Pathway | REAC:R-HSA-4420097 | 0.000579521 | 3.236930649 | NOS3,AKT1,CTNNB1,PIK3CA,CYBB |
| REAC | Diseases of signal transduction by growth factor receptors and second messengers | REAC:R-HSA-5663202 | 0.000683275 | 3.165404297 | MAPK3,EGF,ESR1,AKT1,CTNNB1,PIK3CA,EP300,ESR2 |
| REAC | PI3K/AKT Signaling in Cancer | REAC:R-HSA-2219528 | 0.000795092 | 3.099582449 | EGF,ESR1,AKT1,PIK3CA,ESR2 |
| REAC | Platelet activation, signaling and aggregation | REAC:R-HSA-76002 | 0.000841646 | 3.074870675 | ALB,VEGFA,MAPK3,EGF,AKT1,PIK3CA,SOD1 |
| REAC | Signaling by ERBB4 | REAC:R-HSA-1236394 | 0.001082587 | 2.965537005 | CXCL12,EGF,ESR1,PIK3CA |
| REAC | Intracellular signaling by second messengers | REAC:R-HSA-9006925 | 0.0014489 | 2.8389615 | MAPK3,EGF,ESR1,AKT1,TP53,PIK3CA,ESR2 |
| REAC | Estrogen-dependent nuclear events downstream of ESR-membrane signaling | REAC:R-HSA-9634638 | 0.001822281 | 2.73938456 | MAPK3,EGF,AKT1 |
| REAC | Cellular responses to stress | REAC:R-HSA-2262752 | 0.002488632 | 2.604039375 | ALB,IL6,VEGFA,MAPK3,CAT,TP53,SIRT1,EP300,CYBB,SOD1 |
| REAC | Cellular responses to stimuli | REAC:R-HSA-8953897 | 0.002933691 | 2.532585634 | ALB,IL6,VEGFA,MAPK3,CAT,TP53,SIRT1,EP300,CYBB,SOD1 |
| REAC | FOXO-mediated transcription | REAC:R-HSA-9614085 | 0.003562158 | 2.448286794 | AKT1,CAT,SIRT1,EP300 |
| REAC | VEGFR2 mediated vascular permeability | REAC:R-HSA-5218920 | 0.005310778 | 2.274841884 | NOS3,AKT1,CTNNB1 |
| REAC | Constitutive Signaling by Aberrant PI3K in Cancer | REAC:R-HSA-2219530 | 0.005581069 | 2.25328258 | EGF,ESR1,PIK3CA,ESR2 |
| REAC | Hemostasis | REAC:R-HSA-109582 | 0.007416192 | 2.129819016 | NOS3,ALB,VEGFA,MAPK3,EGF,AKT1,TP53,PIK3CA |
| REAC | MAPK3 (ERK1) activation | REAC:R-HSA-110056 | 0.007592493 | 2.119615594 | IL6,MAPK3 |
| REAC | CD163 mediating an anti-inflammatory response | REAC:R-HSA-9662834 | 0.009486499 | 2.022894033 | IL6,IL10 |
| REAC | Regulation of TP53 Activity through Acetylation | REAC:R-HSA-6804758 | 0.011346824 | 1.945125676 | AKT1,TP53,EP300 |
| REAC | Frs2-mediated activation | REAC:R-HSA-170968 | 0.01158958 | 1.935932313 | NGF,MAPK3 |
| REAC | LRR FLII-interacting protein 1 (LRRFIP1) activates type I IFN production | REAC:R-HSA-3134973 | 0.014808891 | 1.829477455 | CTNNB1,EP300 |
| REAC | Transcriptional Regulation by VENTX | REAC:R-HSA-8853884 | 0.016358788 | 1.786248868 | IL6,TP53,CTNNB1 |
| REAC | Prolonged ERK activation events | REAC:R-HSA-169893 | 0.019150539 | 1.717818992 | NGF,MAPK3 |
| REAC | Generic Transcription Pathway | REAC:R-HSA-212436 | 0.020140106 | 1.695938251 | IL6,VEGFA,MAPK3,ESR1,AKT1,CAT,TP53,SIRT1,CTNNB1,EP300,ESR2 |
| REAC | Transcriptional regulation by the AP-2 (TFAP2) family of transcription factors | REAC:R-HSA-8864260 | 0.023315224 | 1.632360414 | VEGFA,ESR1,EP300 |
| REAC | Signaling by NTRK1 (TRKA) | REAC:R-HSA-187037 | 0.028760929 | 1.541197094 | NGF,MAPK3,PIK3CA,EP300 |
| REAC | Detoxification of Reactive Oxygen Species | REAC:R-HSA-3299685 | 0.029734665 | 1.526736952 | CAT,CYBB,SOD1 |
| REAC | Signaling by ERBB2 | REAC:R-HSA-1227986 | 0.031344777 | 1.50383481 | EGF,AKT1,PIK3CA |
| REAC | Intrinsic Pathway for Apoptosis | REAC:R-HSA-109606 | 0.03144282 | 1.502478509 | MAPK3,AKT1,TP53 |
| REAC | Tetrahydrobiopterin (BH4) synthesis, recycling, salvage and regulation | REAC:R-HSA-1474151 | 0.031512082 | 1.501522903 | NOS3,AKT1 |
| REAC | eNOS activation | REAC:R-HSA-203615 | 0.038481363 | 1.414749554 | NOS3,AKT1 |
| REAC | ROS and RNS production in phagocytes | REAC:R-HSA-1222556 | 0.040269019 | 1.395028953 | NOS3,CYBB,MPO |
| REAC | PI3K/AKT activation | REAC:R-HSA-198203 | 0.044059502 | 1.355960415 | NGF,PIK3CA |
| REAC | RNA Polymerase II Transcription | REAC:R-HSA-73857 | 0.048145648 | 1.31744296 | IL6,VEGFA,MAPK3,ESR1,AKT1,CAT,TP53,SIRT1,CTNNB1,EP300,ESR2 |
| REAC | RAF-independent MAPK1/3 activation | REAC:R-HSA-112409 | 0.048444423 | 1.314756216 | IL6,MAPK3 |
| WP | Orexin receptor pathway | WP:WP5094 | 5.93E-09 | 8.227135382 | ADIPOQ,NOS3,TNF,IL6,VEGFA,MAPK3,IL10,IL4 |
| WP | Folate metabolism | WP:WP176 | 1.74E-08 | 7.759170717 | ALB,TNF,IL6,IL4,CAT,TP53,SOD1,MPO |
| WP | Hepatitis C and hepatocellular carcinoma | WP:WP3646 | 1.07E-07 | 6.971973986 | IL6,VEGFA,MAPK3,PTGS2,AKT1,TP53 |
| WP | Burn wound healing | WP:WP5055 | 1.28E-07 | 6.892265536 | CXCL12,TNF,IL6,VEGFA,EGF,AKT1,TP53,MMP3 |
| WP | Sudden infant death syndrome (SIDS) susceptibility pathways | WP:WP706 | 1.59E-07 | 6.799278772 | NGF,TNF,IL6,VEGFA,IL10,CTNNB1,GJA1,EP300,ESR2 |
| WP | MicroRNAs in cardiomyocyte hypertrophy | WP:WP1544 | 1.79E-07 | 6.746973349 | TNF,MAPK3,EDN1,EGF,AKT1,CTNNB1,PIK3CA,AGT |
| WP | Malignant pleural mesothelioma | WP:WP5087 | 3.23E-07 | 6.491047629 | NGF,CXCL12,IL6,VEGFA,MAPK3,IL10,EGF,AKT1,TP53,CTNNB1,PIK3CA,MMP3 |
| WP | Netrin-UNC5B signaling pathway | WP:WP4747 | 3.92E-07 | 6.406284211 | TNF,MAPK3,IL10,AKT1,TP53,PIK3CA |
| WP | Network map of SARS-CoV-2 signaling pathway | WP:WP5115 | 4.63E-07 | 6.334270795 | CXCL12,ALB,TNF,IL6,IL10,PTGS2,IL4,AKT1 |
| WP | Relationship between inflammation, COX-2 and EGFR | WP:WP4483 | 5.21E-07 | 6.283512525 | MAPK3,PTGS2,ESR1,AKT1,PIK3CA |
| WP | PI3K-Akt signaling pathway | WP:WP4172 | 6.86E-07 | 6.163603443 | NGF,NOS3,IL6,VEGFA,MAPK3,EGF,IL4,AKT1,TP53,PIK3CA |
| WP | IL-18 signaling pathway | WP:WP4754 | 7.08E-07 | 6.149719423 | ADIPOQ,TNF,IL6,VEGFA,MAPK3,IL10,PTGS2,TP53,CTNNB1,MMP3 |
| WP | Neuroinflammation and glutamatergic signaling | WP:WP5083 | 7.52E-07 | 6.123812023 | NGF,TNF,IL6,MAPK3,IL10,IL4,AKT1 |
| WP | T-cell antigen receptor (TCR) pathway during Staphylococcus aureus infection | WP:WP3863 | 1.17E-06 | 5.933527989 | TNF,MAPK3,IL10,IL4,AKT1,PIK3CA |
| WP | Endometrial cancer | WP:WP4155 | 1.29E-06 | 5.890781872 | MAPK3,EGF,AKT1,TP53,CTNNB1,PIK3CA |
| WP | Acute viral myocarditis | WP:WP4298 | 1.42E-06 | 5.848544637 | TNF,IL6,MAPK3,IL10,EDN1,AKT1 |
| WP | Resistin as a regulator of inflammation | WP:WP4481 | 2.29E-06 | 5.639440596 | TNF,IL6,MAPK3,AKT1,PIK3CA |
| WP | Spinal cord injury | WP:WP2431 | 2.56E-06 | 5.591278825 | TNF,IL6,MAPK3,PTGS2,IL4,TP53,GJA1 |
| WP | Breast cancer pathway | WP:WP4262 | 2.94E-06 | 5.531358165 | MAPK3,EGF,ESR1,AKT1,TP53,CTNNB1,PIK3CA,ESR2 |
| WP | Selenium micronutrient network | WP:WP15 | 3.53E-06 | 5.452453187 | ALB,TNF,IL6,PTGS2,CAT,SOD1,MPO |
| WP | Overview of proinflammatory and profibrotic mediators | WP:WP5095 | 5.35E-06 | 5.27201306 | CXCL12,TNF,IL6,VEGFA,IL10,IL4 |
| WP | TNF-related weak inducer of apoptosis (TWEAK) signaling pathway | WP:WP2036 | 6.19E-06 | 5.208122896 | TNF,IL6,MAPK3,AKT1,CTNNB1 |
| WP | Lung fibrosis | WP:WP3624 | 6.63E-06 | 5.17846595 | TNF,IL6,EDN1,EGF,IL4 |
| WP | EGFR tyrosine kinase inhibitor resistance | WP:WP4806 | 7.43E-06 | 5.129025805 | IL6,VEGFA,MAPK3,EGF,AKT1,PIK3CA |
| WP | Photodynamic therapy-induced NF-kB survival signaling | WP:WP3617 | 8.06E-06 | 5.09368235 | TNF,IL6,VEGFA,PTGS2,MMP3 |
| WP | Pancreatic adenocarcinoma pathway | WP:WP4263 | 9.84E-06 | 5.007005974 | VEGFA,MAPK3,EGF,AKT1,TP53,PIK3CA |
| WP | Cytokines and inflammatory response | WP:WP530 | 1.14E-05 | 4.944025703 | TNF,IL6,IL10,IL4 |
| WP | COVID-19 adverse outcome pathway | WP:WP4891 | 2.11E-05 | 4.674706896 | TNF,IL6,IL10,AGT |
| WP | Integrated breast cancer pathway | WP:WP1984 | 2.29E-05 | 4.639358464 | VEGFA,ESR1,AKT1,TP53,SIRT1,CTNNB1,EP300 |
| WP | Aryl hydrocarbon receptor Netpath | WP:WP2586 | 2.64E-05 | 4.5779592 | TNF,VEGFA,PTGS2,ESR1,EP300 |
| WP | VEGFA-VEGFR2 signaling pathway | WP:WP3888 | 3.17E-05 | 4.49945472 | NOS3,ALB,VEGFA,MAPK3,PTGS2,AKT1,CTNNB1,PIK3CA,GJA1,CYBB |
| WP | Allograft Rejection | WP:WP2328 | 3.61E-05 | 4.442836484 | CXCL12,TNF,VEGFA,IL10,IL4 |
| WP | Gastrin signaling pathway | WP:WP4659 | 4.64E-05 | 4.333703652 | VEGFA,MAPK3,PTGS2,AKT1,CTNNB1,PIK3CA |
| WP | Angiogenesis | WP:WP1539 | 4.78E-05 | 4.321017261 | NOS3,VEGFA,AKT1,PIK3CA |
| WP | IL-4 signaling pathway | WP:WP395 | 5.30E-05 | 4.275981218 | MAPK3,IL4,AKT1,PIK3CA,EP300 |
| WP | TGF-beta receptor signaling | WP:WP560 | 6.94E-05 | 4.158854289 | TNF,MAPK3,EGF,CTNNB1,EP300 |
| WP | Transcription factor regulation in adipogenesis | WP:WP3599 | 8.12E-05 | 4.090677303 | ADIPOQ,TNF,IL6 |
| WP | Caloric restriction and aging | WP:WP4191 | 8.17E-05 | 4.087920998 | AKT1,TP53,SIRT1 |
| WP | Alzheimer's disease | WP:WP5124 | 8.75E-05 | 4.057965732 | TNF,IL6,MAPK3,PTGS2,AKT1,CTNNB1,PIK3CA,CYBB |
| WP | TGF-beta receptor signaling in skeletal dysplasias | WP:WP4816 | 9.72E-05 | 4.012302203 | TNF,MAPK3,EGF,CTNNB1,EP300 |
| WP | RAC1/PAK1/p38/MMP2 pathway | WP:WP3303 | 0.000102114 | 3.990914235 | MAPK3,AKT1,TP53,CTNNB1,PIK3CA |
| WP | Chromosomal and microsatellite instability in colorectal cancer | WP:WP4216 | 0.000111383 | 3.953181704 | MAPK3,PTGS2,AKT1,TP53,CTNNB1 |
| WP | Galanin receptor pathway | WP:WP4970 | 0.000111992 | 3.95081115 | ADIPOQ,IL6,VEGFA |
| WP | Non-small cell lung cancer | WP:WP4255 | 0.000126475 | 3.897994954 | MAPK3,EGF,AKT1,TP53,PIK3CA |
| WP | SARS-CoV-2 innate immunity evasion and cell-specific immune response | WP:WP5039 | 0.000127823 | 3.893392554 | CXCL12,TNF,IL6,IL10 |
| WP | T-cell activation SARS-CoV-2 | WP:WP5098 | 0.000140713 | 3.85166561 | TNF,MAPK3,IL4,AKT1,TP53 |
| WP | Photodynamic therapy-induced HIF-1 survival signaling | WP:WP3614 | 0.000149853 | 3.824334778 | VEGFA,PTGS2,EDN1,TP53 |
| WP | Vitamin B12 metabolism | WP:WP1533 | 0.000150107 | 3.82360036 | ALB,TNF,IL6,SOD1,MPO |
| WP | Head and neck squamous cell carcinoma | WP:WP4674 | 0.000165858 | 3.780263875 | VEGFA,AKT1,TP53,CTNNB1,PIK3CA |
| WP | Oncostatin M signaling pathway | WP:WP2374 | 0.000207615 | 3.682741885 | VEGFA,MAPK3,AKT1,TP53,MMP3 |
| WP | Overview of nanoparticle effects | WP:WP3287 | 0.000239535 | 3.620630284 | TNF,IL6,PTGS2 |
| WP | Aspirin and miRNAs | WP:WP4707 | 0.000281589 | 3.550383556 | NOS3,VEGFA,PTGS2 |
| WP | ErbB signaling pathway | WP:WP673 | 0.000385175 | 3.414342352 | MAPK3,EGF,AKT1,TP53,PIK3CA |
| WP | Glioblastoma signaling pathways | WP:WP2261 | 0.000418815 | 3.377977762 | MAPK3,AKT1,TP53,PIK3CA,EP300 |
| WP | Prostaglandin signaling | WP:WP5088 | 0.000456775 | 3.340297764 | TNF,IL6,VEGFA |
| WP | Hepatitis B infection | WP:WP4666 | 0.000473452 | 3.324723787 | TNF,IL6,MAPK3,AKT1,PIK3CA,EP300 |
| WP | Corticotropin-releasing hormone signaling pathway | WP:WP2355 | 0.000493947 | 3.306320035 | NOS3,MAPK3,AKT1,CTNNB1,GJA1 |
| WP | Alzheimer's disease and miRNA effects | WP:WP2059 | 0.000511886 | 3.290827046 | TNF,IL6,MAPK3,PTGS2,AKT1,CTNNB1,PIK3CA,CYBB |
| WP | Cardiac hypertrophic response | WP:WP2795 | 0.000523609 | 3.280993124 | TNF,MAPK3,EGF,AKT1 |
| WP | Fibrin complement receptor 3 signaling pathway | WP:WP4136 | 0.000589097 | 3.229812921 | TNF,IL6,AKT1,PIK3CA |
| WP | Thymic stromal lymphopoietin (TSLP) signaling pathway | WP:WP2203 | 0.000769977 | 3.113522505 | IL6,MAPK3,AKT1,PIK3CA |
| WP | Toll-like receptor signaling pathway | WP:WP75 | 0.00078679 | 3.104141188 | TNF,IL6,MAPK3,AKT1,PIK3CA |
| WP | Amyotrophic lateral sclerosis (ALS) | WP:WP2447 | 0.000833645 | 3.079018773 | TNF,CAT,TP53,SOD1 |
| WP | Male infertility | WP:WP4673 | 0.001108394 | 2.955305905 | NOS3,TNF,ESR1,CAT,EP300,ESR2 |
| WP | Focal adhesion: PI3K-Akt-mTOR-signaling pathway | WP:WP3932 | 0.00123194 | 2.909410488 | NGF,NOS3,VEGFA,MAPK3,EGF,AKT1,PIK3CA |
| WP | DNA damage response (only ATM dependent) | WP:WP710 | 0.001234549 | 2.908491603 | AKT1,CAT,TP53,CTNNB1,PIK3CA |
| WP | Focal adhesion | WP:WP306 | 0.001277496 | 2.893640394 | VEGFA,MAPK3,EGF,AKT1,CTNNB1,PIK3CA |
| WP | MAPK signaling pathway | WP:WP382 | 0.001566591 | 2.805044346 | NGF,TNF,MAPK3,EGF,AKT1,TP53 |
| WP | Leptin signaling pathway | WP:WP2034 | 0.001661204 | 2.779577069 | NOS3,MAPK3,ESR1,AKT1 |
| WP | IL-6 signaling pathway | WP:WP364 | 0.001784624 | 2.748453384 | IL6,MAPK3,AKT1,AGT |
| WP | EV release from cardiac cells and their functional effects | WP:WP3297 | 0.002125494 | 2.672540174 | CXCL12,TNF |
| WP | Development and heterogeneity of the ILC family | WP:WP3893 | 0.002655604 | 2.57583671 | TNF,IL6,IL4 |
| WP | NAD metabolism in oncogene-induced senescence and mitochondrial dysfunction-associated senescence | WP:WP5046 | 0.002880097 | 2.540592873 | IL6,TP53,SIRT1 |
| WP | Altered glycosylation of MUC1 in tumor microenvironment | WP:WP4480 | 0.002973912 | 2.526671939 | TNF,IL6 |
| WP | PI3K/AKT/mTOR - VitD3 signaling | WP:WP4141 | 0.003006041 | 2.522005168 | IL10,AKT1,PIK3CA |
| WP | Melanoma | WP:WP4685 | 0.003388896 | 2.469941755 | MAPK3,AKT1,TP53,PIK3CA |
| WP | AMP-activated protein kinase (AMPK) signaling | WP:WP1403 | 0.003388896 | 2.469941755 | ADIPOQ,AKT1,TP53,PIK3CA |
| WP | Estrogen signaling pathway | WP:WP712 | 0.003450967 | 2.462059156 | ESR1,AKT1,PIK3CA |
| WP | Ebola virus pathway in host | WP:WP4217 | 0.003770054 | 2.423652457 | MAPK3,IL4,AKT1,PIK3CA,EP300 |
| WP | Copper homeostasis | WP:WP3286 | 0.003834741 | 2.416263958 | AKT1,TP53,PIK3CA,SOD1 |
| WP | TNF-alpha signaling pathway | WP:WP231 | 0.003861592 | 2.413233642 | TNF,IL6,MAPK3,AKT1 |
| WP | Regulation of toll-like receptor signaling pathway | WP:WP1449 | 0.00397159 | 2.401035576 | TNF,IL6,MAPK3,AKT1,PIK3CA |
| WP | Pathways affected in adenoid cystic carcinoma | WP:WP3651 | 0.004396574 | 2.356885644 | AKT1,TP53,PIK3CA,EP300 |
| WP | Nonalcoholic fatty liver disease | WP:WP4396 | 0.006184565 | 2.20869084 | ADIPOQ,TNF,IL6,AKT1,PIK3CA |
| WP | Neovascularisation processes | WP:WP4331 | 0.006324856 | 2.198949376 | CXCL12,MAPK3,AKT1 |
| WP | Non-genomic actions of 1,25 dihydroxyvitamin D3 | WP:WP4341 | 0.006709224 | 2.173327704 | TNF,IL6,MAPK3 |
| WP | miRNAs involvement in the immune response in sepsis | WP:WP4329 | 0.006941184 | 2.158566441 | TNF,IL6,IL10 |
| WP | Epithelial to mesenchymal transition in colorectal cancer | WP:WP4239 | 0.007172034 | 2.144357647 | MAPK3,AKT1,TP53,CTNNB1,PIK3CA |
| WP | Pluripotent stem cell differentiation pathway | WP:WP2848 | 0.007184752 | 2.1435882 | IL6,VEGFA,EGF |
| WP | PI3K-AKT-mTOR signaling pathway and therapeutic opportunities | WP:WP3844 | 0.007816023 | 2.107014167 | NOS3,AKT1,PIK3CA |
| WP | AGE/RAGE pathway | WP:WP2324 | 0.00849496 | 2.070838644 | NOS3,MAPK3,AKT1,SOD1 |
| WP | IL-17 signaling pathway | WP:WP2112 | 0.00951564 | 2.021561988 | MAPK3,AKT1,PIK3CA |
| WP | Somatroph axis (GH) and its relationship to dietary restriction and aging | WP:WP4186 | 0.010061628 | 1.997331735 | AKT1,SIRT1 |
| WP | Adipogenesis | WP:WP236 | 0.010681865 | 1.971352935 | ADIPOQ,TNF,IL6,CTNNB1,AGT |
| WP | Cells and molecules involved in local acute inflammatory response | WP:WP4493 | 0.011196428 | 1.950920519 | TNF,IL6 |
| WP | IL-10 anti-inflammatory signaling pathway | WP:WP4495 | 0.011638379 | 1.934107504 | IL6,IL10 |
| WP | Extracellular vesicles in the crosstalk of cardiac cells | WP:WP4300 | 0.012277326 | 1.910896208 | IL6,EGF,SOD1 |
| WP | Bladder cancer | WP:WP2828 | 0.012489454 | 1.90345656 | VEGFA,EGF,TP53 |
| WP | Type II diabetes mellitus | WP:WP1584 | 0.013578082 | 1.867161571 | ADIPOQ,TNF |
| WP | Small cell lung cancer | WP:WP4658 | 0.014263344 | 1.845778656 | PTGS2,AKT1,TP53,PIK3CA |
| WP | Androgen receptor signaling pathway | WP:WP138 | 0.014873385 | 1.827590176 | AKT1,SIRT1,CTNNB1,EP300 |
| WP | LTF danger signal response pathway | WP:WP4478 | 0.015622019 | 1.806262828 | TNF,IL6 |
| WP | Antiviral and anti-inflammatory effects of Nrf2 on SARS-CoV-2 pathway | WP:WP5113 | 0.016273215 | 1.788526634 | TNF,IL6,MMP3 |
| WP | RAS and bradykinin pathways in COVID-19 | WP:WP4969 | 0.017055961 | 1.768123815 | NOS3,TNF,AGT |
| WP | CAMKK2 pathway | WP:WP4874 | 0.017999522 | 1.744739026 | MAPK3,SIRT1,EP300 |
| WP | Kisspeptin/kisspeptin receptor system in the ovary | WP:WP4871 | 0.018693899 | 1.728300111 | MAPK3,AKT1,PIK3CA |
| WP | Vitamin D in inflammatory diseases | WP:WP4482 | 0.018976852 | 1.721775822 | TNF,IL6 |
| WP | Genes controlling nephrogenesis | WP:WP4823 | 0.021415044 | 1.669281029 | CXCL12,VEGFA,CTNNB1 |
| WP | Photodynamic therapy-induced AP-1 survival signaling | WP:WP3611 | 0.024049383 | 1.61889606 | TNF,IL6,TP53 |
| WP | Oxidative stress response | WP:WP408 | 0.024714892 | 1.607041275 | CAT,CYBB,SOD1 |
| WP | Control of immune tolerance by vasoactive intestinal peptide | WP:WP4484 | 0.027691232 | 1.557657729 | IL10,IL4 |
| WP | Mammary gland development pathway - Puberty (Stage 2 of 4) | WP:WP2814 | 0.031921404 | 1.495918009 | EGF,ESR1 |
| WP | Translation inhibitors in chronically activated PDGFRA cells | WP:WP4566 | 0.032275725 | 1.491123996 | MAPK3,AKT1,PIK3CA |
| WP | Circadian rhythm genes | WP:WP3594 | 0.03345506 | 1.475538186 | ADIPOQ,IL6,TP53,SIRT1,EP300 |
| WP | TLR4 signaling and tolerance | WP:WP3851 | 0.035613581 | 1.448384354 | TNF,IL6 |
| WP | Synaptic signaling pathways associated with autism spectrum disorder | WP:WP4539 | 0.038659908 | 1.412739188 | MAPK3,AKT1,PIK3CA |
| WP | Signal transduction through IL1R | WP:WP4496 | 0.048608752 | 1.313285531 | TNF,IL6 |
| WP | Wnt signaling pathway | WP:WP363 | 0.049940896 | 1.30154367 | AKT1,CTNNB1,GJA1 |
